# Systemic Treatment Strategies Beyond Chemotherapy in Recurrent or Advanced Endometrial Cancer: A Systematic Review and Meta-Analysis

**DOI:** 10.3390/cancers18132091

**Published:** 2026-06-27

**Authors:** István Madár, Anett Szabó, Bianca Golzio Navarro Cavalcante, Gábor Vleskó, Péter Hegyi, Nándor Ács, Tamás Kói, Emma Kálovics, Gábor Szabó

**Affiliations:** 1 Centre for Translational Medicine, Semmelweis University, 1088 Budapest, Hungary; madar.istvan@semmelweis.hu (I.M.); szabo.anett2@semmelweis.hu (A.S.); golzio.bianca@semmelweis.hu (B.G.N.C.); vlesko.gabor@semmelweis.hu (G.V.); hegyi.peter@semmelweis.hu (P.H.); acs.nandor@semmelweis.hu (N.Á.); koi.tamas@semmelweis.hu (T.K.); kalovics.emma@stud.semmelweis.hu (E.K.); 2 Department of Obstetrics and Gynecology, Semmelweis University, 1088 Budapest, Hungary; 3 Department of Urology, Semmelweis University, 1082 Budapest, Hungary; 4 Institute for Translational Medicine, Medical School, University of Pécs, 7624 Pécs, Hungary; 5 Institute of Pancreatic Diseases, Semmelweis University, 1083 Budapest, Hungary; 6 Stochastics Department, Budapest University of Technology and Economics, 1111 Budapest, Hungary

**Keywords:** endometrial cancer, uterine cancer, chemotherapy, immune checkpoint inhibitor, pembrolizumab, lenvatinib, dostarlimab, avelumab, durvalumab, atezolizumab, immunotherapy, progestin, progesterone, aromatase inhibitor, progression-free survival, PFS, overall survival, OS, treatment-related adverse events, recurrent, advanced

## Abstract

**Introduction**: Optimal systemic treatment for recurrent or advanced endometrial cancer (EC) remains uncertain, particularly in the second-line setting. While first-line chemotherapy with paclitaxel and carboplatin is widely used, its efficacy is limited. Recent evidence suggests that adding immune checkpoint inhibitors (ICIs) to chemotherapy (ChT) can improve progression-free survival (PFS). In addition, hormonal therapies (such as progestins and aromatase inhibitors), targeted therapies and ICIs used alone or in combination are emerging as potential treatment options. **Objectives**: Our objective was to systematically evaluate the efficacy and safety of systemic therapies beyond standard ChT for patients with recurrent or advanced EC, focusing on progression-free survival (PFS), overall survival (OS), and treatment-related adverse events (TRAEs). **Methods**: We conducted a systematic review and meta-analysis of randomized controlled trials (RCTs), prospective studies, and retrospective studies up to June 2024 on PubMed, Embase, and CENTRAL. Studies evaluating systemic therapies—chemotherapy, hormonal therapy, targeted agents, and ICIs—in recurrent or advanced EC were included. Primary outcomes were PFS and OS; secondary outcomes included grade ≥ 3 treatment-related adverse events. Risk of bias was assessed using the Cochrane RoB 2 and MINORS tools, and GRADE was applied to evaluate the certainty of evidence. **Results**: Five RCTs evaluated the addition of ICI to conventional ChT. In mismatch repair-deficient (MMRd) patients, the addition of ICI significantly improved PFS and OS compared to the ChT-only group. In mismatch repair-deficient (MMRd) patients, the median PFS (mPFS) was 8.39 months in the ChT group and 22.73 months in the ICI group (HR: 0.34, *p* < 0.001). OS results were 26.48 and 41.36 months, respectively. In mismatch repair-proficient (MMRp) patients, mPFS was 9.4 months in the ChT group and 10.18 months at the ICI group (HR: 0.72, *p* = 0.002). OS was 27.37 and 27.79 months, respectively. We conducted several exploratory single-arm subgroup analyses and multiple individual patient data (IPD) meta-analyses across several clinically relevant subgroups, including patients treated with progestins, lenvatinib plus pembrolizumab, PI3K/AKT/mTOR inhibitor monotherapy, and PI3K/AKT/mTOR inhibitors in combination with aromatase inhibitors. Detailed descriptive analyses were performed for each subgroup. **Conclusions**: Combining chemotherapy with ICIs appears to show the most favorable survival outcomes, especially in MMRd tumors. Taking into account the methodological limitations inherent in the elaboration of lower-level evidence and IPD, hormonal and targeted therapies might be considered viable options, with efficacy and safety profiles dependent on tumor biology. Better stratification of the EC patient cohort is warranted.

## Simple Summary

Recurrent or advanced endometrial cancer is difficult to treat, and standard chemotherapy often stops working, leaving patients with limited options. This research reviews newer treatments, including immunotherapy, hormone-based treatments, and targeted drugs, to understand which approaches are most effective and safest. We aim to compare how long patients live without their disease worsening, overall survival, and serious side effects across different therapies. Our findings suggest that combining chemotherapy with immunotherapy appears to offer the greatest benefit, especially for patients with certain tumor characteristics (mismatch repair-deficient tumors), while other treatments may still provide meaningful alternatives depending on the biology of cancer. Yet, the data should be interpreted with caution in light of the lower level of evidence. These results can help guide treatment decisions and inform future research on improving outcomes for patients with advanced endometrial cancer.

## 1. Introduction

Endometrial cancer (EC) ranks among the most prevalent cancers in women globally, with its incidence steadily increasing [1]. The lifetime risk of developing endometrial cancer for women is around 3%, with the median age of diagnosis being 61 years. Over the past three decades, the incidence of this cancer has increased by 132%, largely due to the growing prevalence of risk factors such as obesity and an aging population [2].

Since EC typically presents with abnormal uterine bleeding, it is often diagnosed at an early stage, resulting in a better survival rate compared to other gynecological malignancies. However, stage III–IV endometrial cancer continues to pose a significant burden, as survival rates at these advanced stages are considerably worse than in early-stage disease [2]. Primary metastatic disease accounts for less than 10% of EC patients [3]. In patients with EC, the likelihood of relapse is approximately 10–15% for those with stage I-II disease [4]. This risk rises significantly up to 70% in cases with advanced EC stages [5,6].

Endometrial cancer was traditionally categorized into two subtypes: type I and type II. Recently, however, it has become evident that this classification does not adequately capture the complexity and heterogeneity of the disease. In 2013, The Cancer Genome Atlas (TCGA) introduced a molecular classification of endometrial cancer, identifying four distinct subgroups: ultramutated DNA polymerase epsilon (POLE), microsatellite instability (MSI) hypermutated, low copy number, and high copy number [7,8]. This approach is included in the latest FIGO classification system and is being increasingly adopted in recent studies and therapy decisions. However, approximately 50% of endometrial cancer patients lack specific molecular alterations and are classified under the non-specific molecular profile group (NSMP) [9,10]. Although specific molecular profiles show a better response to individualized therapies, the combination of carboplatin and paclitaxel serves as the standard systemic first-line chemotherapy for managing primary advanced or recurrent EC [11]. Despite its widespread use, the long-term prognosis remains unfavorable, with a median overall survival typically less than three years [12,13]. In addition, standard combination chemotherapy (ChT) regimens are generally not as effective as second-line treatments, and they present high toxicity. Furthermore, standard monotherapy options yield only modest outcomes [14]. Hence, other systemic therapies can be considered in specific patient groups. Emerging evidence suggests that the addition of immune checkpoint inhibitors (ICIs) to ChT may offer survival benefits. ICIs such as pembrolizumab, when combined with lenvatinib, a multi-kinase inhibitor, show consistent efficacy [15]. While molecular profiling is a newer tool for guiding therapy, estrogen receptor (ER) and progesterone receptor (PR) positivity have long influenced treatment decisions in recurrent and advanced disease [2,16].

Although progestins and megestrol acetate therapy are widely used for advanced and recurrent endometrial cancer patients because of their low toxicity, the results among studies are inconsistent [17]. With proven benefits in breast cancer, aromatase inhibitors such as letrozole and anastrozole are involved in the blockage of natural synthesis of estrogen and demonstrate antitumor effects in EC [18].

Targeted therapies (TT) for EC exploit molecular aberrations to selectively inhibit tumor growth while preserving normal cells. The PI3K/AKT/mTOR (phosphatidylinositol-3 kinase/serine-threonine kinase/mammalian target of rapamycin) pathway is frequently dysregulated in EC and can be targeted by several inhibitors. These agents, used alone or in combination with chemotherapy or hormonal therapies, demonstrate efficacy in tumors harboring PIK3CA mutations, though resistance and toxicity remain challenges [19]. Other possibilities include angiogenesis inhibition, such as with anti-VEGF agents, which disrupt angiogenesis, thus curbing tumor progression. A possible treatment of HER2-positive EC can be Trastuzumab, which, when combined with ChT, impedes HER2-mediated signaling. In tumors with MSI-H (microsatellite instability-high) and MMR deficiency, immune checkpoint inhibitors demonstrate clinical efficacy [20].

There is currently no universally agreed-upon standard of care for second-line treatment in advanced EC, as guidelines vary widely. Additionally, effective therapeutic options for managing recurrent disease remain limited [12].

Despite these advances, treatment recommendations beyond first-line chemotherapy remain unclear, and comparative data across therapeutic strategies are limited. A detailed analysis of adverse events is lacking in the literature. This systematic review and meta-analysis aim to evaluate the efficacy and safety of systemic therapies beyond conventional ChT in patients with recurrent or advanced EC, with a focus on PFS, OS and TRAEs.

## 2. Methods

### 2.1. Protocol and Registration

This work was carried out as part of the Systems Education Program at Semmelweis University and conducted within the Translational Medicine (TM) Cycle Framework by the Academia Europaea [21,22].

This systematic review and meta-analysis were performed in accordance with the Preferred Reporting Items for Systematic Reviews and Meta-Analyses (PRISMA) 2020 guidelines [22,23,24] (Supplementary Table S1). The study protocol was pre-registered on PROSPERO (registration number CRD42023400040), and the analysis was conducted in strict compliance with the registered protocol.

### 2.2. Eligibility Criteria

We included randomized controlled trials (RCTs), non-randomized interventional studies, and prospective and retrospective observational studies that investigated systemic therapeutic agents in recurrent or advanced endometrial cancer (EC). Eligible interventions included immune checkpoint inhibitors (ICIs), targeted therapies (TT), and hormonal therapies (HT), either as a single therapy or in combination with chemotherapy.

Our clinical question was formulated based on the PICO framework. Eligible patients (P) were women aged ≥ 18 years with histologically confirmed recurrent or FIGO stage III–IV EC. We pooled and compared different systemic therapy schemes (I and C) in the case of randomized controlled trials. For single-arm studies, we pooled similar agents.

The primary outcomes (O) of our study were progression-free survival (PFS) and overall survival (OS). Secondary outcomes were efficacy and safety, including grade ≥ 3 treatment-related adverse events (TRAEs).

We excluded case reports, case series, reviews, conference abstracts, and non-English publications. The included articles contained data on PFS, and most of them also contained data on OS and adverse events. When multiple studies were conducted on the same cohort, the most recent article was included in our analysis. We did not include articles on chemotherapies other than paclitaxel and carboplatine/cisplatine. Where the reduced-dose therapy scheme and usual-dose therapy scheme were both within one study, the usual dose was included in the quantitative analysis. Detailed exclusion criteria is available in Supplementary Appendix S1.

### 2.3. Information Sources

A systematic search in MEDLINE (via PubMed), Embase, and the Cochrane Central Register of Controlled Trials (CENTRAL) was performed on 3 June 2024. A manual search on the reference lists of eligible articles was performed. No filters or restrictions were applied.

### 2.4. Search Strategy

The following search key terms were used: endometrial cancer AND (recurrent OR advanced) AND systemic therapy. Full search strategies for each database are available in Supplementary Appendix S2.

### 2.5. Selection and Data Collection Process

All records were imported into EndNote 20 (Clarivate Analytics, Philadelphia, PA, USA), followed by selection using Rayyan. After removing duplicate records, the title-abstract screening and full-text review were conducted independently by two authors (I.M. and G.V.). Cohen’s kappa coefficient (κ = 0.86 for title-abstract and κ = 0.92 for full-text selection) was calculated to evaluate inter-rater agreement. Any disagreements were resolved through discussion with a third reviewer (N.Á.).

A standardized data extraction form was used. Data extraction was performed independently by two authors (I.M. and GV). In instances where multiple publications reported data from the same cohort, the most complete or expert-reviewed dataset was prioritized. Therapeutic agents with similar acting mechanisms, or combinations containing similar therapeutic agents, with Kaplan–Meyer curves with data on PFS or OS available were pooled.

Given the limited availability of randomized controlled trials in this setting, single-arm data were included to provide a comprehensive overview of available therapeutic strategies; however, these analyses were considered exploratory and not intended for direct comparative inference.

### 2.6. Data Items

The following data were extracted: first author, year of publication, study population, study period, study type, number of previous chemotherapy treatment lines, intervention chemotherapy agent, comparator chemotherapy agent (when data available), patients data (total number, histology, age, molecular classification data), data on PFS and OS for both groups, and data on efficacy and safety (duration of response, overall response rate, adverse events, grade ≥ 3 adverse events).

### 2.7. Study Risk of Bias Assessment

Risk of bias was independently assessed by two reviewers (I.M. and G.V.) using the RoB 2 tool for randomized controlled trials and the MINORS tool for non-randomized and single-arm interventional studies. Disagreements were resolved by consensus or consultation with a third reviewer (N.Á.).

### 2.8. Certainty of Evidence

Evidence quality was evaluated using the Grading of Recommendations Assessment, Development, and Evaluation (GRADE) framework [25]. Two reviewers (I.M. and G.V.) independently assessed each outcome and comparison, with disagreements resolved by a third reviewer (G.S.).

### 2.9. Synthesis Methods

Both qualitative and quantitative syntheses of the data were performed. The prespecified minimum number of clinically poolable studies for performing a meta-analysis was three. Hazard ratios from placebo-controlled RCTs were pooled in a separate analysis. The inverse variance method was used to calculate pooled HR, using the natural logarithm of HR and its SE from the available data following the methodology of Tierney et al. 2007 [26]. To estimate the heterogeneity variance measure (tau-square), the restricted maximum-likelihood estimator was used with the Q profile method for confidence intervals [27,28]. The Hartung–Knapp adjustment was applied when estimating confidence intervals for the pooled effect [29]. Heterogeneity was assessed by Higgins and Thompson I^2^ statistics [30]. Results were considered statistically significant if the CI did not contain 1 for HR. Also, individual patient data (IPD) meta-analysis was performed for different treatment modalities separately. IPD was retrieved from published Kaplan–Meier (KM) curves using plot digitalization tools (WebPlotDigitizer version 4, Ankit Rohatgi, Pacifica, CA, USA, accessed on 12 December 2024) [31] and following the methodology of Liu et al. [32] (2021). Pooled survival probabilities for predefined time intervals and pooled median survival times (with 95% confidence intervals) were estimated for each treatment modality. To this end, the multivariate method described in Combescure et al. was implemented [33]. The Greenwood formula was used to estimate confidence intervals for pooled survival probabilities. We summarized our findings in forest plots and tables. The statistical analysis was conducted in the R statistical environment (R Core Team) using the ‘meta’ [31], ‘dmetar’ [34], ‘IPDfromKM’ [32] and ‘metaSurvival’ packages. Due to limited access to original patient-level data, formal external validation of reconstructed datasets was not feasible; however, visual inspection and comparison with published survival summaries were performed to ensure consistency.

## 3. Results

### 3.1. Search and Selection

**Figure 1:**
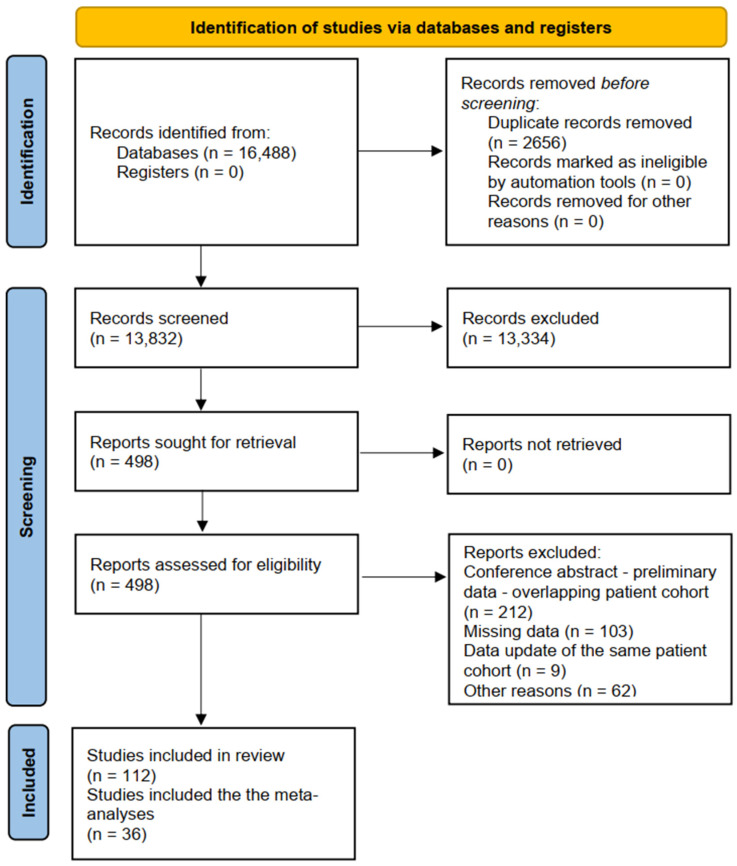
PRISMA 2020 flowchart representing the study selection process.

A total of 16,488 records were identified. After the removal of 2656 duplicates, 13,832 titles and abstracts were screened. Of these, 498 articles were retrieved for full-text review; a total of 112 studies met the eligibility criteria and were included in the final analysis. The selection process is illustrated in the PRISMA flow diagram (Figure 1).

### 3.2. Basic Characteristics of Included Studies

The baseline characteristics of the 116 included studies are summarized in Table 1 and Supplementary Table S2, whilst Supplementary Table S3 includes data on treatment-related adverse events (TRAEs). Studies included a range of systemic therapies for recurrent or advanced endometrial cancer, which were categorized into three main groups: hormonal therapy, immune checkpoint inhibitors and other targeted therapies. These agents were evaluated either as monotherapies or in combination with chemotherapy or one another. Study designs included both randomized controlled trials and prospective or retrospective cohort studies. The number of patients included in the studies ranged from 6 to 927, and the median age of the patients ranged between 53 and 71 years.

**RCT-based data**

### 3.3. Immune Checkpoint Inhibitors (ICIs)

#### 3.3.1. ICI Combination with Chemotherapy (Paclitaxel and Carboplatin)

Five RCTs (phase II and III trials) compared ChT (1149 patients) groups with ChT+ICIs (including pembrolizumab, dostarlimab, avelumab, durvalumab and atezolizumab) (1311 patients) [117,118,119,120,121]. In three studies, these therapies were applied as first-line treatment [119,120,121]; in the other two studies [117,118], prior systemic and local treatments were permitted.

**Figure 2:**
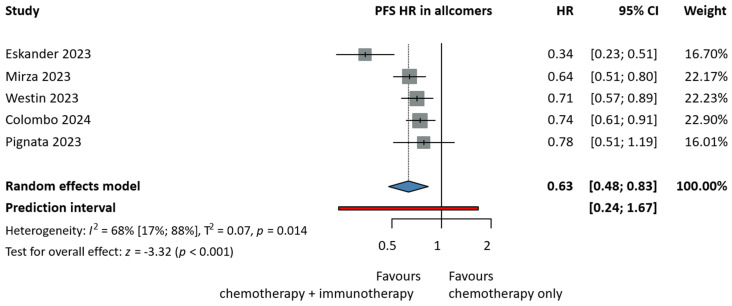
Forest plot of progression-free survival (PFS) hazard ratios (HR) comparing chemotherapy plus immunotherapy versus chemotherapy alone in all comers. Articles cited: Eskander et al. [117], Mirza et al. [118], Westin et al. [120], Colombo et al. [121], Pignata et al. [119]. Abbreviations: PFS: progression-free survival. HR: hazard ratio. CI: confidence interval.

**Figure 3:**
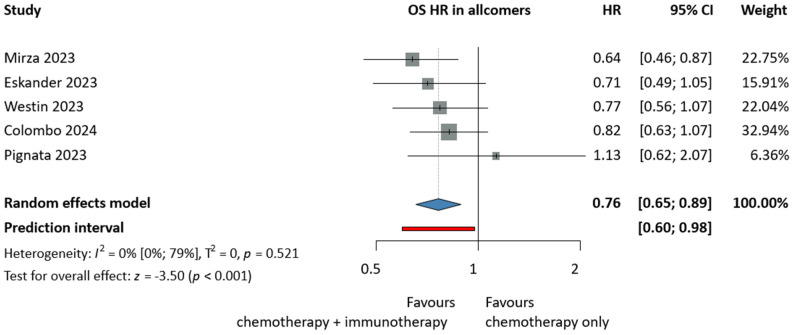
Forest plot of overall survival (OS) hazard ratios (HR) comparing chemotherapy plus immunotherapy versus chemotherapy alone in all comers. Articles cited: Eskander et al. [117], Mirza et al. [118], Westin et al. [120], Colombo et al. [121], Pignata et al. [119]. Abbreviations: OS: overall survival. HR: hazard ratio. CI: confidence interval.

In the all-comer populations, ICIs combined with ChT resulted in a pooled mPFS of 12.98 months (9.72–20.57) and an mOS of 33.98 months (29.18–39.07). ChT alone resulted in an mPFS of 9.37 months (7.96–9.87) and an mOS of 28.57 months (24.53–31.17). The combination therapy group had better PFS (HR: 0.63, CI 0.48–0.83); however, heterogeneity was high (I^2^ = 68%), primarily reflecting variability in the magnitude of treatment effect rather than inconsistency in direction, as all studies favored the intervention. OS proved to be better as well (HR: 0.76, CI 0.65–0.89). Heterogeneity was low, as visible in Figure 2 and Figure 3. Notably, the addition of avelumab to ChT resulted in worse OS rates in the all-comer population; mPFS was better when avelumab was added to the ChT regime.

**Figure 4:**
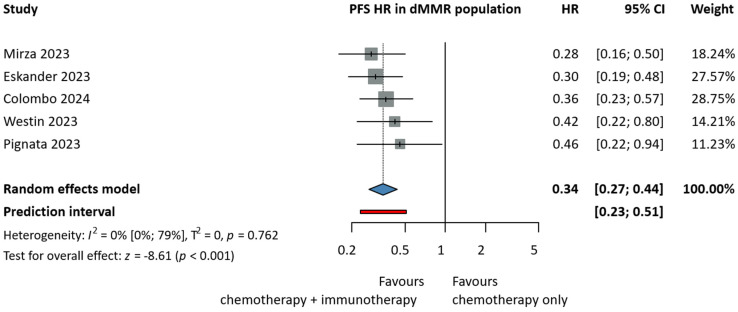
Forest plot of progression-free survival (PFS) hazard ratios (HR) comparing chemotherapy plus immunotherapy versus chemotherapy alone in mismatch repair-deficient patients (dMMR). Articles cited: Eskander et al. [117], Mirza et al. [118], Westin et al. [120], Colombo et al. [121], Pignata et al. [119]. Abbreviations: PFS: progression-free survival. HR: hazard ratio. CI: confidence interval. dMMR: mismatch repair-deficient.

**Figure 5:**
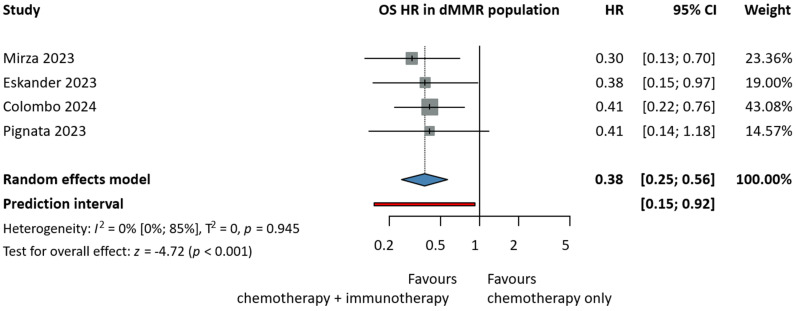
Forest plot of overall survival (OS) hazard ratios (HR) comparing chemotherapy plus immunotherapy versus chemotherapy alone in mismatch repair-deficient patients (dMMR). Articles cited: Eskander et al. [117], Mirza et al. [118], Colombo et al. [121], Pignata et al. [119]. Abbreviations: OS: overall survival. HR: hazard ratio. CI: confidence interval. dMMR: mismatch repair-deficient.

In the MMRd population, a significantly better mPFS (22.73 months, 15.03–28.03) was obtained in therapy regimes involving ICIs than in ChT groups (8.39 months, 6.92–9.79), HR: 0.34 (0.27–0.44), *p* < 0.001 with low heterogeneity. With regard to OS, the addition of ICIs resulted in better OS (41.36 months vs. 26.48 months (18.45–28.76)), HR: 0.38 (0.25–0.56), *p* < 0.001. Figure 4 and Figure 5 contain the forest plots of this subgroup.

**Figure 6:**
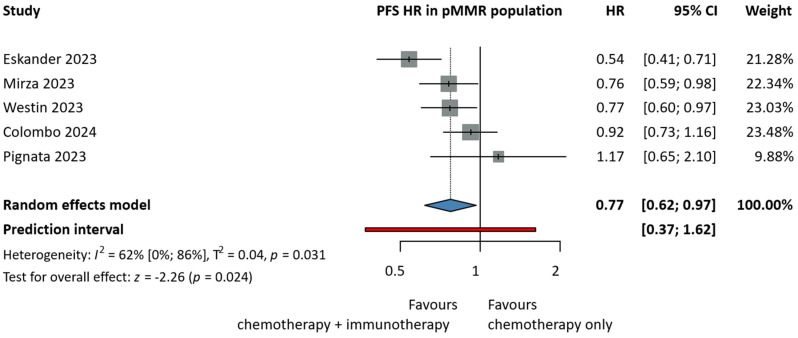
Forest plot of progression-free survival (PFS) hazard ratios (HR) comparing chemotherapy plus immunotherapy versus chemotherapy alone in mismatch repair-proficient (pMMR) patients. Articles cited: Eskander et al. [117], Mirza et al. [118], Westin et al. [120], Colombo et al. [121], Pignata et al. [119]. Abbreviations: PFS: progression-free survival. HR: hazard ratio. CI: confidence interval. pMMR: mismatch repair-proficient.

**Figure 7:**
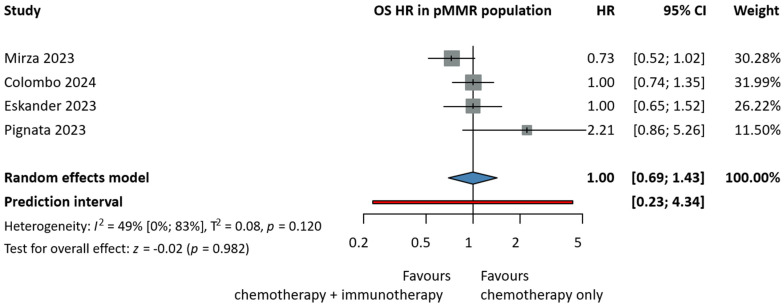
Forest plot of overall survival (OS) hazard ratios (HR) comparing chemotherapy plus immunotherapy versus chemotherapy alone in mismatch repair-proficient (pMMR) patients. Articles cited: Eskander et al. [117], Mirza et al. [118], Colombo et al. [121], Pignata et al. [119]. Abbreviations: OS: overall survival. HR: hazard ratio. CI: confidence interval. dMMR: mismatch repair-proficient.

In MMRp populations, mPFS was superior in the population receiving ICIs as well (mPFS 10.18 (9.19–11.45) vs. 9.4 months (8.27–9.87) in the ChT group), HR: 0.77 (0.62–0.97), *p* = 0.031. Heterogeneity was moderate to high (62%), warranting cautious interpretation of the pooled estimate despite its statistical significance. In the study concerning avelumab, analysis based on MMR status showed that the PFS benefit was driven by the dMMR population, whereas patients with a pMMR tumor did not benefit from the addition of avelumab. Figure 6 and Figure 7 contain the forest plot of this subgroup.

With regard to the TRAEs, these trials collectively indicate that integrating immune checkpoint inhibitors into platinum–taxane chemotherapy regimens maintain a broadly comparable safety profile to chemotherapy alone. While the addition of immunotherapy slightly expands the spectrum of severe adverse events, it does not fundamentally shift the overall toxicity landscape, which continues to be dominated by hematologic complications inherent to cytotoxic chemotherapy. (Supplementary Table S3).

Risk of bias (Supplementary Figure S1) as assessed by the ROB 2 tool was low.

With regard to the publication bias, funnel plots are available as Supplementary Figure S3.

**Non-RCT-based data**

#### 3.3.2. ICIs Combined with Angiogenesis Inhibitors

Nine studies [63,64,65,66,67,68,69,70,71,145] matched the inclusion criteria in this subgroup. Baseline characteristics of the included studies are available in Table 1 and Supplementary Table S2. In all the studies, every patient had received at least one line of ChT prior to lenvatinib and pembrolizumab treatment.

This subgroup included prospective trials and retrospective cohorts evaluating lenvatinib plus pembrolizumab in advanced or recurrent endometrial cancer. Studies included early-phase and pivotal clinical trials (Taylor et al., 2020 [63]; Makker et al., 2023 [71] KEYNOTE-146; Makker et al., 2023 KEYNOTE-775 [67]), as well as retrospective or real-world analyses (How et al., 2021 [65]; Kim et al., 2022 [64]; Zammarrelli et al., 2023 [70]; Chiba et al., 2024 [66]; Tochigi et al., 2024 [69]; Shang et al., 2024 [68]).

Overall, lenvatinib plus pembrolizumab demonstrated clinically meaningful activity across prospective and real-world datasets. In the early-phase study, lenvatinib plus pembrolizumab (Taylor et al., 2020 [63]) showed promising antitumor activity, with a median PFS of 9.7 months; median OS was not reached at the time of analysis. However, toxicity was substantial, with grade ≥ 3 treatment-related adverse events reported in 67% of patients.

Subsequent retrospective studies reported more variable outcomes. Lenvatinib plus pembrolizumab (How et al., 2021 [65]; Kim et al., 2022 [64]; Zammarrelli et al., 2023 [70]) was associated with median PFS values ranging from 4.6 to 6.0 months. In Zammarrelli et al. (2023) [70], median OS was 18.3 months, similar to the OS reported in the pivotal KEYNOTE-775 trial, although high-grade treatment-related adverse events occurred in 84% of patients.

The pivotal randomized phase III trial confirmed the efficacy of this regimen. Lenvatinib plus pembrolizumab (Makker et al., 2023; KEYNOTE-775 [71]) achieved a median PFS of 7.3 months and median OS of 18.7 months in 411 patients, outperforming chemotherapy with doxorubicin or paclitaxel. Benefits in PFS and OS were observed across molecular subgroups, regardless of mismatch repair status. However, grade ≥ 3 treatment-related adverse events were frequent, occurring in 90.1% of patients compared with 73.7% in the chemotherapy control group.

Additional evidence supported the durability and real-world relevance of these findings. Lenvatinib plus pembrolizumab (Makker et al., 2023; KEYNOTE-146 [67]) demonstrated sustained efficacy and acceptable tolerability in previously treated advanced endometrial cancer. Lenvatinib plus pembrolizumab (Tochigi et al., 2024 [69]) showed a median PFS of 8.47 months, consistent with clinical trial outcomes. In Chiba et al. (2024) [66], median PFS was 11.6 months, with grade ≥ 3 adverse events in 80% of patients. This study also suggested that the p53abn molecular subgroup had a poorer prognosis despite treatment with lenvatinib plus pembrolizumab.

The toxicity profile was clinically relevant and distinct from chemotherapy-based regimens. Across studies, grade ≥ 3 adverse events were mainly characterized by hypertension, fatigue, gastrointestinal toxicity, endocrine or metabolic abnormalities, anemia, weight loss, hypothyroidism, and thrombocytopenia. Hypertension was the most consistent high-grade toxicity, reported at high frequency in several real-world cohorts. Fatigue, diarrhea or mucositis, hepatic dysfunction, proteinuria, and immune-related events were also observed. Although hematologic adverse events occurred, they were generally less dominant than in platinum-based chemotherapy (see Supplementary Table S3).

Explorative quantitative analysis of the subgroup is available in Supplementary Appendix S3.

#### 3.3.3. ICIs Combined with Other Substances

Nine phase II trials and retrospective studies [54,55,56,57,58,59,60,61,62] evaluated immune checkpoint inhibitors (ICIs) alone or in combination with Poly (ADP-ribose) Polymerase (PARP) inhibitors in recurrent or advanced EC. The number of patients varied between 8 and 56, all of them pretreated with at least one first-line regime. Treatment efficacies were heterogeneous across cohorts, PFS varying between 1.4 and 7.4 months, OS between 8.4 and 12.5 months. Combination strategies, particularly combining ICIs with anti-angiogenic agents (other than lenvatinib) or PARP inhibitors, demonstrated modest improvements in clinical benefit compared to monotherapy. Across studies, grade ≥ 3 toxicities were frequent but variable, reflecting the heterogeneity of combinations. Hematologic toxicity was prominent in regimens involving PARP, whereas combinations with anti-angiogenic agents (e.g., anlotinib, cabozantinib) showed higher rates of hypertension and proteinuria. Some studies reported substantial overall toxicity rates (e.g., > 60% grade ≥ 3 AEs with nivolumab + cabozantinib), while others showed more moderate or manageable profiles. Data on mPFS and mOS are visible in Table 1 and Supplementary Table S1. Detailed narrative descriptions of these therapies are available in Supplementary Appendix S4.

#### 3.3.4. ICI-Only Therapy Protocols

Seven articles [48,49,50,51,52,53,146] assessed the PD-1 or PD-L1 inhibitors (durvalumab, pembrolizumab, avelumab, nivolumab, retifanlimab) as monotherapies, out of which six studies [48,49,50,51,53,146] were of single-arm, phase II design. In all studies, except for one [51], all the patients had undergone at least one first-line therapy regime.

In this group of studies evaluating immune checkpoint inhibitors mostly in mismatch repair-deficient (dMMR) or microsatellite instability-high (MSI-high) endometrial cancer, median PFS and OS varied across agents (6–23.5 and 30.2–40 months, respectively) but consistently favored immunotherapy in MMRd molecular subtypes (Table 1). Overall, ICI monotherapy was associated with a favorable and manageable safety profile, with grade ≥ 3 TRAEs under 16% in all studies. Detailed narrative descriptions of these therapies are available in Supplementary Appendix S5.

### 3.4. Targeted Therapies

#### 3.4.1. Anti-Angiogenic Agent-Only Therapies

Across 12 phase II [15,125,126,127,128,129,130,131,132,133,134,135,136] trials evaluating anti-angiogenic agents, efficacy and toxicity profiles were variable. PFS varied between 1.97 and 4.6 months, whereas OS varied between 6.3 and 20.2 months. In most studies, the anti-angiogenic therapies were applied after at least one prior therapy line; however, in two studies [134,135], a mixed cohort of patients with no or one prior chemotherapy line was included. Table 1 contains the basic characteristics of the included studies, while Appendix S6 contains a detailed description of the studies.

#### 3.4.2. Anti-Angiogenic Agents Combined with Chemotherapy

Overall, bevacizumab-containing regimens [37,44,45,46,47] consistently improved PFS (typically 8–13 months) with tolerable toxicity profiles. Emerging combinations with HER2-targeted or immune checkpoint agents offer further promise. A detailed description of the studies is provided in Supplementary Appendix S10.

#### 3.4.3. PI3K/AKT/mTOR Inhibitor Monotherapies

This subgroup included 11 studies, including four randomized phase II trials, evaluating inhibitors of the PI3K/AKT/mTOR pathway in predominantly pretreated populations. Agents included temsirolimus (Oza et al., 2011 [107]; Fleming et al., 2014 [103]; Emons et al., 2016 [99]), everolimus (Ray-Coquard et al., 2013 [100]), ridaforolimus (Oza et al., 2015 [108]), pilaralisib (Matulonis et al., 2015 [102]), gedatolisib and PF-04691502 (Del Campo et al., 2016 [110]), apitolisib (Makker et al., 2016 [101]), buparlisib (Heudel et al., 2017 [104]), LY3023414 (Rubinstein et al., 2020 [114]), and MK-2206 (Myers et al., 2020 [112]).

Overall efficacy was modest. Median progression-free survival ranged from approximately 2.0 to 7.33 months, with the longest PFS observed for temsirolimus in chemotherapy-naive patients (Oza et al., 2011 [107]). In chemotherapy-pretreated patients, PFS with temsirolimus was shorter, at 3.25 months (Oza et al., 2011 [107]). Most other agents produced median PFS values of approximately 2.5–4.5 months, while gedatolisib reached 5.9 months (Del Campo et al., 2016 [110]). Median overall survival, where reported, ranged from 8.1 months with everolimus (Ray-Coquard et al., 2013 [100]) to 21.3 months with temsirolimus (Emons et al., 2016 [99]).

Among randomized studies, ridaforolimus (Oza et al., 2015 [108]) showed a statistically significant PFS benefit over control therapy (hormonal therapy and chemotherapy), with a hazard ratio of 0.53 and a *p* = 0.008, although this was accompanied by frequent grade ≥ 3 adverse events. Several other studies showed limited activity or failed to meet predefined efficacy thresholds, including temsirolimus (Emons et al., 2016 [99]), pilaralisib (Matulonis et al., 2015 [102]), apitolisib (Makker et al., 2016 [101]), LY3023414 (Rubinstein et al., 2020 [114]), and MK-2206 (Myers et al., 2020 [112]). Available biomarker analyses did not demonstrate a clear relationship between outcomes and PTEN status or other PI3K/Akt/mTOR pathway markers (Oza et al., 2011 [107]).

Treatment-related adverse events were consistent with PI3K/AKT/mTOR pathway inhibition. The toxicity profile was mainly characterized by metabolic disturbances such as hyperglycemia and hyperlipidemia, gastrointestinal and mucosal events including diarrhea and stomatitis, and hematologic toxicity such as anemia and thrombocytopenia. Fatigue, anorexia, hepatic enzyme elevations, pneumonitis, and thromboembolic events were also reported. Some regimens had substantial grade ≥ 3 toxicity, including apitolisib (Makker et al., 2016) and ridaforolimus (Oza et al., 2015 [108]), and the temsirolimus plus megestrol acetate combination arm was closed because of excess venous thromboses (Fleming et al., 2014 [103]).

Explorative quantitative analysis is available in Supplementary Appendix S7. Supplementary Table S3 contains detailed data on TRAEs.

#### 3.4.4. PI3K/AKT/mTOR Combined with Aromatase Inhibitors

This subgroup included four phase II studies evaluating mTOR-pathway inhibition combined with endocrine therapy in advanced, recurrent, or metastatic endometrial cancer. Treatments included everolimus plus letrozole (Slomovitz et al., 2015 [109]), everolimus plus letrozole plus metformin (Soliman et al., 2020 [105]), vistusertib plus anastrozole (Heudel et al., 2022 [106]), and everolimus plus letrozole versus tamoxifen plus medroxyprogesterone acetate (Slomovitz et al., 2022 [111]).

Overall efficacy was modest to clinically meaningful, with the strongest signals seen in selected endocrine-sensitive populations. Everolimus plus letrozole (Slomovitz et al., 2015 [109]) produced a clinical benefit rate of 40% and an objective response rate of 32%, including several complete responses. Median PFS was 3.0 months, and OS was 14 months, although a subset of responders had prolonged disease control. Serous histology predicted a lack of response, while patients with endometrioid tumors and CTNNB1 mutations appeared to respond favorably.

Everolimus plus letrozole plus metformin (Soliman et al., 2020 [105]) showed a clinical benefit rate of 50% and an objective response rate of 28% in recurrent endometrioid endometrial cancer. Median PFS was 5.7 months, and median OS was 19.6 months. Positive progesterone receptor expression was associated with a higher likelihood of clinical benefit.

In the randomized VICTORIA trial, vistusertib plus anastrozole (Heudel et al., 2022 [106]) improved the 8-week progression-free rate compared with anastrozole alone: 67.3% versus 39.1%. Overall response rates were 24.5% versus 17.4%, and median PFS was 5.2 months versus 1.9 months, respectively. These findings support the activity of adding mTOR inhibition to endocrine therapy in hormone receptor-positive recurrent or metastatic disease.

In the randomized GOG Foundation study, everolimus plus letrozole (Slomovitz et al., 2022 [111]) and tamoxifen plus medroxyprogesterone acetate both demonstrated clinical activity. Response rates were 22% and 25%, respectively, while median PFS was 6 months with everolimus plus letrozole and 4 months with hormonal therapy. Notably, chemotherapy-naive patients treated with everolimus plus letrozole had a median PFS of 28 months, compared with 4 months in patients with prior chemotherapy.

Treatment-related adverse events were consistent with mTOR inhibition and endocrine therapy. Across studies, toxicity was mainly characterized by metabolic effects, particularly hyperglycemia, together with mucositis, fatigue, anemia, gastrointestinal symptoms, and laboratory abnormalities. In vistusertib plus anastrozole (Heudel et al., 2022 [106]), fatigue, lymphopenia, hyperglycemia, and diarrhea were the most common reported vistusertib-associated events. In everolimus plus letrozole (Slomovitz et al., 2022 [111]), grade ≥ 3 adverse events were more frequent than with hormonal therapy, while thromboembolic events (11% of patients) were observed in the hormonal therapy arm but not in the everolimus plus letrozole arm.

Explorative quantitative analysis is available in Supplementary Appendix S8. Supplementary Table S3 contains detailed data on TRAEs.

#### 3.4.5. Other Therapy Possibilities

A variety of other targeted therapies have been evaluated in patients with recurrent or advanced EC, with mixed outcomes. These articles include agents like HER2-targeted therapies, EGFR/ERBB pathway inhibitors, WEE1 inhibitors, microtubule-targeting agents and DRD2 antagonists. A narrative description of these articles is available in Supplementary Appendix S11.

### 3.5. Hormonal Therapies

A range of hormonal and targeted therapies has been evaluated in endometrial cancer, with variable efficacy. Twenty-nine articles [72,73,74,75,76,77,78,79,80,81,82,83,84,85,86,87,88,89,90,91,93,94,95,96,97,98,147,148,149,150] were included in this subgroup, with mPFS ranges from 1 to 17 months. However, most of the studies were published before 2005, and the inclusion criteria and the definitions of progression vary between the studies.

#### Progestins

This subgroup included studies evaluating progestins, estrogen receptor antagonists, aromatase inhibitor-based combinations, GnRH analogs, progesterone receptor antagonists, LHRH-targeted agents, CDK4/6 inhibitors, and chemotherapy–hormonal therapy combinations in recurrent or advanced endometrial cancer.

Overall, single-agent hormonal therapies showed modest but generally well-tolerated activity. Medroxyprogesterone acetate and megestrol acetate (Thigpen et al., 1999 [83]; Thigpen et al., 2001 [82]; Whitney et al., 2004 [81]) produced median PFS values of approximately 3.0–3.2 months and median OS values of 8.8–13 months, with generally low rates of grade ≥ 3 adverse events. More recent retrospective data suggested that the benefit may be greater in selected lower-grade tumors: progestin therapy (Kulkarni et al., 2023 [91]) was associated with median PFS of 15.7 months and OS of 25.9 months in grade 1–2 recurrent endometrial cancer, compared with 5 months and 12.5 months, respectively, in grade 3 disease. In patients with at least 1 prior line of chemotherapy, PFS was 6.2 months, while OS was 23 months.

Fulvestrant (Covens et al., 2011 [74]; Emons et al., 2013 [78]) demonstrated limited efficacy, with median PFS of 1.6–2.3 months and median OS of 9–13.2 months, although toxicity was minimal. Similarly, GnRH analogs, including leuprolide, goserelin, and triptorelin (Covens et al., 1996 [85]; Asbury et al., 2002 [96]; Lhommé et al., 1999 [89]), showed limited activity, with median PFS of 1.9–2.8 months and median OS of 7.2–9 months, but were associated with few severe adverse events. Mifepristone (Ramondetta et al., 2009 [97]) also showed very limited activity, with fatigue and dyspnea reported as notable toxicities.

Among investigational or targeted hormonal approaches, AEZS-108 (Emons et al., 2014 [79]), a doxorubicin-linked LHRH agonist, achieved a median PFS of 7 months and median OS of 15 months in LHRH receptor-positive patients. Severe toxicity was mainly hematologic, particularly neutropenia and leukopenia. CDK4/6 inhibitor-based approaches appeared more promising. Ribociclib (Colon-Otero et al., 2020 [73]) was reported as safe and active in estrogen receptor-positive endometrial cancer, with a median PFS of 5.4 months and OS of 18.9 months. Letrozole plus abemaciclib (Konstantinopoulos et al., 2023 [98]) showed encouraging activity in estrogen receptor-positive disease, with a median PFS of 9.1 months and manageable hematologic toxicity.

Combination strategies using chemotherapy with hormonal therapy showed variable efficacy. Regimens such as chemotherapy plus hormonal therapy (Ayoub et al., 1988 [86]; Hoffman et al., 1989 [93]; Bafaloukos et al., 1999 [72]; Karagol et al., 2006 [75]) produced median PFS values of approximately 4–6 months in most studies and were commonly associated with hematologic and gastrointestinal toxicity. In hormonal therapy versus chemotherapy [75], median PFS was 5 months and 4 months, and median OS was 16 months and 11 months, respectively, although the difference was not statistically significant.

The toxicity profile of hormonal therapy was generally milder than that of chemotherapy or modern targeted combinations, but thromboembolic and metabolic events were recurrent safety signals. Severe events were uncommon with several hormonal agents, but pulmonary embolism, thrombophlebitis, arterial thrombosis, hyperglycemia, hypertension, edema, hepatic toxicity, and anemia were reported across progestin-containing regimens [76,81,83,87,88]. Targeted combinations had a broader toxicity profile, including metabolic, hematologic, hepatic, and fatigue-related adverse events [103,109].

Explorative quantitative analysis is available in Supplementary Appendix S9. Supplementary Table S3 contains detailed data on TRAEs.

### 3.6. Risk of Bias and Grade Assessment

Most of the single-arm studies had a low risk of bias (see Supplementary Table S4).

GRADE-pro was performed for the RCT subgroup, indicating high certainty for the MMRd subgroup. For the non-RCT subgroups, GRADE indicated a very low level of certainty, suggesting careful interpretation of the results (Supplementary Figure S2).

## 4. Discussion

Our study provided a broad overview of systemic therapeutic possibilities, concentrating on the role of targeted therapies, immune checkpoint inhibitors, hormonal therapies and their combinations in recurrent or advanced EC. EC is a heterogeneous disease, with several histological and molecular subtypes; therefore, numerous therapeutic choices are available. Based on our meta-analyses, currently, the best PFS and OS in recurrent or advanced EC appears to be provided by ChT combined with ICIs in MMRp patients. However, we must point out that our study is based on significantly heterogeneous data and levels of evidence, making the clinical interpretability limited.

The addition of ICIs slightly increases the prevalence of TRAEs; these are manageable. MMRd/MSI-H patients clearly benefited from the addition of ICIs. In combination therapy, the PFS and OS of MMRd patients surpassed those of MMRp patients, except when avelumab was used. Notably, in one study, two different protocols of follow-up therapy were compared: follow-up therapy with a PARP inhibitor yielded the best PFS and OS (Durva-Olap-CP) in the overall population [120]. Also of note, in the included RCTs, the analysis was based mostly on patients receiving therapy as first-line treatment or more than 6 months after the first-line treatment. This could have led to better PFS and OS because of the patients’ better general condition. ICI combinations showed slightly higher rates of grade 3–4 TRAEs versus chemotherapy alone. Yet, the overall toxicity burden remains primarily driven by chemotherapy and is not substantially exacerbated by the addition of checkpoint inhibitors. Hematologic toxicities consistently represent the principal grade ≥ 3 adverse events across all regimens. While immunotherapy introduces additional adverse events, these are generally low in frequency and include immune-related and systemic complications such as hypertension, thromboembolism, and infections. Importantly, differences between intervention and comparator arms are modest and inconsistent across trials, with no clear indication of a markedly increased risk of severe toxicity associated with the incorporation of checkpoint inhibitors. Heterogeneity varied substantially across endpoints and subgroups, with direct implications for the strength of evidence. In the overall population, PFS showed moderate-to-high heterogeneity (I^2^ = 68%) with a wide prediction interval crossing unity, limiting confidence in the generalizability of the effect despite consistent direction. In contrast, OS demonstrated no detectable heterogeneity (I^2^ = 0%), supporting a more robust and consistent survival benefit. In subgroup analyses, findings diverged markedly. The dMMR population showed no heterogeneity (I^2^ = 0%) for both PFS and OS, with consistent effect sizes and prediction intervals entirely below unity, indicating a stable and reproducible benefit. Conversely, the pMMR subgroup exhibited moderate heterogeneity for both PFS (I^2^ = 62%) and OS (I^2^ = 49%), with wide prediction intervals crossing unity and, for OS, a null pooled effect, reflecting substantial uncertainty and inconsistency across studies. Overall, these results indicate that the observed treatment benefit is driven primarily by the dMMR population, while evidence in pMMR remains heterogeneous and less conclusive, warranting cautious interpretation. The wide prediction intervals crossing the null value indicate substantial uncertainty regarding the effect expected in an individual future study.

We also reviewed other systemic therapy possibilities and performed exploratory pooling of the single-arm studies. The best pooled second-line therapies in our study were the lenvatinib and pembrolizumab combination and mTOR inhibitors combined with aromatase inhibitors. However, findings derived from single-arm pooled estimates should be interpreted with caution, as they do not allow for direct comparison between treatment modalities. As these estimates are derived from lower-level evidence, their generalizability may be limited. The exploratory nature of the results, particularly those based on single-arm data from the four subgroup analyses, should be emphasized.

The lenvatinib and pembrolizumab combination was superior to the physicians’ choice chemotherapy protocol in a randomized controlled study including 827 patients (411 vs. 416 patients) [71] (PFS in intention to treat population: 7.2 vs. 3.8 months, *p* < 00.001, OS 18.3 vs. 11.4 months, *p* < 0.001) with an increased ≥grade 3 TRAE rate. Of note, the benefits of this treatment were observed regardless of MMR status, and the majority of patients exhibited tumor regression, irrespective of histological subtype. To reduce side effects, dose reduction was investigated in a study by How et al. [65], concluding that a reduced dose results in similar PFS and OS. This study provided the worst PFS in our cohort (3.2 in the recommended dose vs. 5.5 months in the reduced dose cohort), which can be attributed to a lower proportion of endometrioid tumors, more patients with ≥ 2 prior ChT lines and the inclusion of patients with worse performance status. Most real-world studies [64,66,69,70] showed similar PFS and OS rates to those in our analysis. Of note, a study conducted by Kim et al. [64] showed lower treatment response rates but concluded that this protocol is feasible as it offers acceptable treatment response and manageable TRAEs. Zammarelli et al. [70] reported significantly better 1-year PFS in patients with reduced lenvatinib doses and similar OS, with no difference in PFS and OS in black patients compared to other racial groups. Across trials, the toxicity profile differed from chemotherapy-based regimens and was dominated by hypertension, fatigue, and gastrointestinal adverse events, along with endocrine and hepatic effects. Hypertension was the most consistent and prominent grade ≥ 3 adverse event, occurring in roughly 25–50% of patients in several studies. Other frequent toxicities included fatigue, diarrhea/colitis, liver dysfunction, and proteinuria, with occasional serious immune-related or organ-specific events. The role of lenvatinib and pembrolizumab following first-line chemoimmunotherapy remains unclear, as no studies included in our analysis have specifically evaluated lenvatinib–pembrolizumab in this setting.

ICIs containing only protocols were associated with a more favorable and manageable safety profile than many combination strategies. The best results were obtained in patients with a MMRd molecular subtype or Lynch syndrome [51].

PI3K/AKT/mTOR inhibitors combined with aromatase inhibitors provided an exploratory pooled median PFS of 5.33 months and OS of 15.89 months in the four included studies. In addition to substantial heterogeneity, the single-arm design of the included studies warrants careful consideration when interpreting these results. In the randomized phase II trial of Slomowitz et al. [111], chemotherapy-naïve patients treated with letrozole and everolimus showed better PFS than the ChT group of the pivotal GOG-209 trial [13] (28 vs. 14 months). This finding suggests a need for more clinical trials involving everolimus combined with letrozole as a first-line treatment. Patients with endometrial histology showed better response rates. In the other included study, the addition of metformin to letrozole and everolimus resulted in a 50% clinical benefit rate and a 28% overall response in women with recurrent EC [105]. Studies highlight the need for novel biomarkers for predicting response.

In contrast, PI3K/AKT/mTOR inhibitors demonstrated limited activity as monotherapy, with PFS generally ranging from 2 to 7.3 months and rarely surpassing 5 months. The most favorable outcome was observed in the study by Oza et al. [107], but this was confined to the chemotherapy-naïve subgroup and did not meet the predefined primary efficacy endpoints in previously treated patients.

MMRd/MSI-H tumor patients provided outstanding response rates, PFS and OS across the included trials. The best PFS was obtained by pembrolizumab, durvalumab and retifanlimab monotherapy protocols. Real-world data by Colomba et al. [50] confirmed that the efficacy of immunotherapy remained consistent across both first-line and later-line settings, suggesting that patients may derive benefit irrespective of the line of systemic therapy. The combination of immunotherapy and systemic therapies offers modest results, with PFS ranging from 1.4 to 7.4 months. Real-world data from Cui et al. [59] concerning amlotinib combined with pembrolizumab demonstrated a similar effect.

Progestin therapy represents another option, particularly for patients with low-grade, hormone receptor-positive disease. These have been utilized in the treatment of recurrent or advanced EC for several decades. In our exploratory pooled analysis, limited by the low-level evidence studies and methodological shortcomings, progestin therapy resulted in an mPFS of 3.6 months in a heterogeneous study population. By contrast, data from Kulkarni et al. [91] (retrospective study, with the inherent limitations of selection bias but reflecting real-world practice) demonstrated a substantially longer median PFS of 14.3 months. Notably, chemotherapy-naïve patients in this cohort achieved a PFS of 17.9 months, whereas those previously treated with chemotherapy exhibited a significantly shorter PFS of 6.2 months. Most of the patients received megestrol acetate. The superior outcomes reported in this study may be explained by the relatively homogeneous composition of the patient population, which was characterized by favorable prognostic features such as endometrioid histology, low tumor grade, and hormone receptor positivity. Comparable results have been reported in the GOG 209 trial [13], where chemotherapy-naïve patients achieved a PFS of 13–14 months, although overall survival (OS) was higher, ranging between 37 and 41 months. Similarly, Pautier et al. [76] reported consistent findings with hormonal therapy, further supporting its clinical relevance in selected patient groups. Other medications targeting the hormonal cascade, such as fulvestrant, irosustat, anastrozole, leuprolide, exametastane, triptorelin, tamoxiphen, danazole, goserelin or combined with other substances resulted in an mPFS between 1 and 9.1 months. Of note, patients were usually not selected based on their hormonal status. A combination of abemaciclib and letrozole [98] resulted in the best PFS (9.1 months).

The guidelines of the major gynecologic oncologic societies (NCCN-National Comprehensive Cancer Network, BGSC-British Gynaecological Cancer Society, ESGO/ESTRO/ESP-European Society of Gynaecological Oncology/European Society for Radiotherapy and Oncology/ European Society of Pathology) [12,151,152] issued until our data extraction recommend the combination of paclitaxel and carboplatine as first-line chemotherapy in recurrent or advanced EC. Our results showed that both the MMRd and MMRp groups profited from the ChT and ICI combinations, especially from the addition of pembrolizumab and dostarlimab. This justifies the addition of ICIs to first-line treatment.

In the management of recurrent and advanced endometrial carcinoma, international guidelines show broad agreement on the principles of systemic treatment, while differing in scope and emphasis. All three major societies—NCCN, BGCS, and ESGO—acknowledge the role of hormonal therapy in carefully selected patients. Low-grade, hormone receptor-positive tumors with indolent clinical behavior, long disease-free intervals, and limited metastatic burden (particularly lung-only disease) are consistently highlighted as suitable candidates. Whereas NCCN [151] provides the most extensive list of hormonal options, including progestins, tamoxifen, aromatase inhibitors, fulvestrant, and even combination approaches such as everolimus with letrozole, ESGO [12] offers specific recommended agents and doses, and BGCS [152] presents a more pragmatic framework, identifying hormonal therapy as a first-line systemic treatment in this subset.

Chemotherapy remains the cornerstone of systemic management for patients with more aggressive or symptomatic diseases. Across all three guidelines, carboplatin combined with paclitaxel is endorsed as the standard first-line regimen. BGCS and ESGO [12,152] place stronger emphasis on the timing of relapse, with platinum rechallenge considered only in patients with a long platinum-free interval, whereas NCCN [151] expands on the therapeutic landscape by also listing carboplatin/docetaxel, the addition of bevacizumab, and the incorporation of trastuzumab in HER2-positive uterine serous carcinoma or carcinosarcoma. ESGO [12] and BGCS [152], in contrast, both acknowledge the absence of a universally accepted second-line chemotherapy standard, although doxorubicin and paclitaxel are noted as the most active agents.

The integration of immunotherapy and targeted agents has introduced greater divergence between guidelines, reflecting both the rapid evolution of evidence and differing regulatory landscapes. All three recommend PD-1 blockade in tumors with mismatch repair deficiency or high microsatellite instability. NCCN [151] is the most detailed, specifying multiple agents (dostarlimab, nivolumab, avelumab) and expanding biomarker-driven therapy and the use of lenvatinib plus pembrolizumab for pMMR/MSS tumors after platinum failure. ESGO [12] also endorses pembrolizumab monotherapy for MMRd/MSI-H disease and pembrolizumab with lenvatinib in MSS/pMMR settings but explicitly notes that access may be constrained by reimbursement. BGCS [152] recommends checkpoint inhibitors more generally in MMRd, POLE-mutated, or high tumor mutational burden cancers.

ESGO [12] provides a distinctive focus on the management of locoregional recurrences, underscoring the central role of radiotherapy—particularly modern image-guided external beam radiotherapy and brachytherapy—in previously unirradiated patients. This contrasts with NCCN [151] and BGCS [152], which predominantly address systemic approaches.

The randomized NRG Oncology/GOG trial by Klopp et al. [153] provides the most relevant prospective evidence regarding the addition of concurrent chemotherapy to definitive radiotherapy for locoregional recurrence. Among 165 patients, adding weekly cisplatin did not improve progression-free survival and increased acute toxicity. Three-year PFS was 73% with radiotherapy alone and 62% with chemoradiation. However, most participants had low-grade endometrioid tumors and isolated vaginal recurrences. Therefore, radiotherapy alone appears appropriate for many radiation-naïve patients with low-grade vaginal recurrence, whereas the relevance of combined systemic treatment for high-risk histologies and pelvic or para-aortic nodal recurrences remains less certain.

Adjuvant evidence provides context but should not be directly extrapolated to recurrent disease. PORTEC-3 [154] showed a long-term survival benefit with chemoradiotherapy, greatest in p53-abnormal tumors, but little benefit from chemotherapy intensification in dMMR disease. GOG-258 [155] found improved locoregional control but no survival benefit. Together, these findings suggest that the value of combined local and systemic therapy depends on disease distribution, recurrence risk, and molecular subtype.

The combination of radiotherapy and immune checkpoint inhibition also has a strong biological rationale. Radiation may promote immunogenic cell death, tumor-antigen release, dendritic cell activation, and lymphocyte infiltration, potentially enhancing immune checkpoint blockade. This concept may be particularly relevant to immunogenic molecular subtypes such as dMMR/MSI-high and POLE-mutated tumors. Nevertheless, prospective evidence in recurrent or oligometastatic endometrial cancer remains limited, and no completed randomized trial has demonstrated a clinical synergistic effect in this specific setting. Relevant ongoing studies, including NRG-GY020 and the MMRd-GREEN component of the RAINBO program, are evaluating combinations of radiotherapy and immunotherapy in earlier disease settings [156].

### 4.1. Strengths and Limitations

This systematic review followed a pre-registered protocol and adhered to PRISMA guidelines, enhancing methodological transparency and reproducibility. Strengths of our study include following a rigorous methodology and the inclusion of both univariate and multivariate analyses to improve robustness. Several updated meta-analyses were performed, providing a comprehensive synthesis of the evolving literature on systemic therapy for recurrent or advanced endometrial cancer. Most included studies confirmed progression using Response Evaluation Criteria in Solid Tumors (RECIST) 1.1 criteria [157], offering consistency in outcome measurement.

However, this review also has several limitations. The included studies exhibited significant heterogeneity in patient populations, treatment protocols, and study designs, which may limit the comparability of results. A substantial proportion of the analyses was based on single-arm studies, which inherently limit comparative interpretation due to the absence of control groups. Such designs are susceptible to selection bias, confounding, and heterogeneity in patient populations, prior treatments, and outcome definitions.

Therefore, pooled estimates from single-arm analyses should be regarded as descriptive and hypothesis-generating rather than as definitive evidence of comparative efficacy. In many cases, direct comparisons between therapies were lacking, which reduces the strength of conclusions drawn from pooled estimates. We acknowledge that the available evidence did not provide a sufficiently connected and homogeneous network of studies with common comparators to permit a robust network meta-analysis.

Reconstruction of individual patient data from Kaplan–Meier curves is subject to potential inaccuracies, including digitization errors and variability in curve resolution. The method relies on assumptions regarding the distribution of censoring and events over time, which may not fully reflect the original data. Consequently, survival estimates derived from reconstructed IPD should be interpreted as approximations rather than exact representations of original patient-level data. These limitations may introduce estimation bias, particularly in studies with sparse reporting or complex survival patterns.

We acknowledge that several other subgroup analyses could have been performed based on tumor and patient characteristics. However, the available studies frequently lacked sufficiently detailed molecular, histopathological, and treatment-sequencing data, preventing robust interpretation of these subgroups.

Publication bias could not be reliably assessed, as most analyses included a small number of studies, rendering funnel plot interpretation and statistical tests unreliable. Therefore, the potential influence of publication bias on the pooled estimates cannot be excluded.

Follow-up durations were generally short, limiting the ability to evaluate long-term outcomes. Several studies also presented a moderate to high risk of bias, particularly in allocation concealment and outcome reporting. In analyses of ICI+ChT combinations, differences in trial design—such as the inclusion of carcinosarcoma cases, treatment duration, and varying proportions of recurrent or heavily pretreated cases and different distributions of high-grade cases across studies—hinder direct cross-trial comparisons. Furthermore, molecular subgroup data beyond MMR status were often incomplete, limiting stratified analyses by TCGA classification.

### 4.2. Implication for Practice and Research

**Figure 8:**
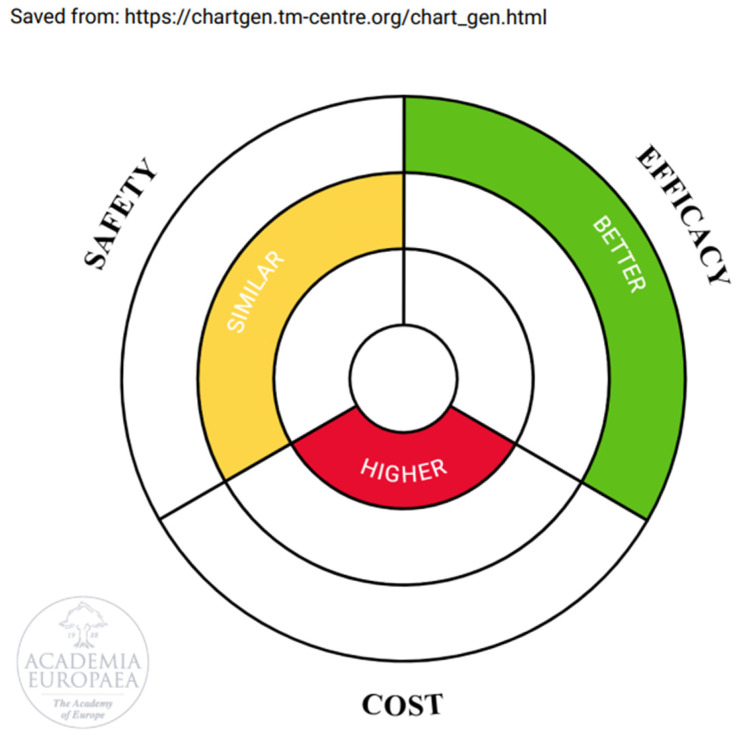
Ring diagram. Saved from: https://chartgen.tm-centre.org/chart_gen.html accessed on 10 January 2026.

On the basis of previous evidence, there are clear benefits of rapidly integrating results into clinical practice, as suggested in the Academia Europaea framework [24,158]. Adding ICIs to ChT appears to be more effective and safer, especially in MMRd populations. Yet, this elevates the financial burdens, so careful patient selection might be needed (see Figure 8).

This review supports the growing clinical role of immunotherapy, particularly in combination with chemotherapy for MMRd tumors. Incorporating molecular profiling into treatment decision-making can be essential to advancing personalized care in endometrial cancer. However, standard treatment strategies for NSMP and p53-abnormal subtypes remain undefined and warrant focused investigation. The growing integration of molecular classification into prognostic assessment and therapeutic decision-making represents a fundamental shift toward individual-based care. However, the clinical value of this approach will depend on equitable access to molecular testing and guideline-concordant treatment, particularly because substantial disparities in diagnosis, treatment delivery, and mortality persist.

Future research should prioritize prospective, multicenter, randomized trials with extended follow-up and predefined molecular and histological subgroup analyses. Inclusion of novel biomarkers beyond the TCGA classification could also result in a better stratification of the patients. Greater inclusion of racially and ethnically diverse populations is also critical to ensure the generalizability of findings. Additionally, comparative studies evaluating ICI monotherapy versus combination regimens—particularly in newly diagnosed or recurrent MMRd disease—are needed to optimize treatment algorithms. Finally, ongoing real-world data collection will be essential to validate trial findings and guide clinical implementation.

## 5. Conclusions

Chemotherapy combined with immune checkpoint inhibitors appears to provide the best PFS and OS, with manageable TRAEs for recurrent or advanced endometrial cancer, with the greatest benefit in MMRd tumors. Further randomized studies should be directed toward the application of ICIs, hormonal therapies, aromatase inhibitors and their combinations based on careful patient selection. Treatment options for NSMP and p53-abnormal subtypes remain limited, and optimal treatment sequencing is still unclear. Further prospective studies are needed to optimize subtype-specific, personalized therapy.

## Figures and Tables

**Table 1:** Basic characteristics of included studies.

Author	Year	Study Type	Nr of Previous ChT Lines	Intervention Therapy			Comparator Therapy			Intervention Group		Comparator Group	
				Component 1	Component 2	Component 3	Component 1	Component 2	Component 3	mPFS (mths)	mOS (mths)	mPFS (mths)	mOS (mths)
Lorusso [35]	2019	phase II multicenter, open label, randomized	0–1	Paclitaxel	Carboplatin	NA	Paclitaxel	Carboplatine	Bevacizu-mab	10.5	29.7	13.7	40
Simpkins [36]	2015	phase II trial	≥1	Paclitaxel	Carboplatin	Bevacizumab	NA	NA	NA	18	58	NA	NA
Aghajanian1 [37]	2018	phase II randomized	0	Paclitaxel	Carboplatin	Bevacizumab	Paclitaxel	Carboplatin	NA	ND	ND	ND	ND
Liao [38]	2022	single-center, randomized	ND	Nivolumab	Bevacizumab	NA	Paclitaxel	Bevacizumab	NA	ND	33.2	ND	28.1
Rose [39]	2017	retrospective	0/1	Paclitaxel	Carboplatin	Bevacizumab	NA	NA	NA	20	56	NA	NA
Kelkar [40]	2022	retrospective	>=1	ChT	Bevacizumab	NA	Hormonal	NA	NA	5	10	5.5	9
Kelkar [41]	2023	retrospective	>=1	ChT	Bevacizumab	NA	Immunotherapy	NA	NA	4	7	29	ND
Wright [42]	2007	retrospective	>=1	Bevacizumab	ChT	NA	NA	NA	NA	5.4	ND	NA	NA
Fader [43]	2020	phase II	0–2	Carboplatin	Paclitaxel	Trastuzu-mab	Carboplatin	Paclitaxel	NA	12.9	29.6	8	24.4
Aghajanian1 [37]	2018	phase II randomized	0	Carboplatin	Ixabepilone	Bevacizumab	NA	NA	NA	ND	ND	ND	ND
Aghajanian [44]	2011	phase II trial	>=1	Bevacizumab	NA	NA	NA	NA	NA	4.17	10.55	NA	NA
Alvarez [45]	2013	phase II trial	>=1	Bevacizumab	Temsirolimus	NA	NA	NA	NA	5.6	16.9	NA	NA
Rubinstein [46]	2021	retrospective	>=1	Bevacizumab	NA	NA	NA	NA	NA	4.9	4.5	NA	NA
Roque [47]	2015	retrospective review	>=1	Bevacizumab	Ixabepilone	NA	Ixabepilone	NA	NA	6.5	9.6	3	4.2
O’Malley [48]	2022	phase II	>=1	Pembrolizumab	NA	NA	NA	NA	NA	13.1	ND	NA	NA
Konstantinopoulos [49]	2019	phase II trial	>=1	Avelumab	NA	NA	NA	NA	NA	40% at 6 mths	ND	NA	NA
Colomba [50]	2023	multicenter, retrospective	>=1	ICI	NA	NA	NA	NA	NA	10.7	31.9	NA	NA
Bellone [51]	2022	phase II trial	0	Pembrolizumab	NA	NA	NA	NA	NA	23.5	40	NA	NA
Antill [52]	2021	phase II	1 to 3	Durvalumab (MMRd)	NA	NA	Durvalumab (MMRp)	NA	NA	8.3	ND	1.8	ND
Berton	2024	phase I	>=1	Retifanlimab	NA	NA	NA	NA	NA	12.2	30.2	NA	NA
André [53]	2023	phase 1	>=1	Dostarlimab	NA	NA	NA	NA	NA	6	NR	NA	NA
Rubinstein [54]	2023	phase II,	>=1	Durvalumab	NA	NA	Durvalumab	Tremelimumab	NA	7.4	ND	7.9	ND
Post [55]	2022	phase II multicenter	>=1	Durvalumab	Olaparib	NA	NA	NA	NA	3.4	8.4	NA	NA
Konstantinopoulos [56]	2022	phase II single-center	>=1	Avelumab	Talazoparib	NA	NA	NA	NA	3.6	ND	NA	NA
Wei [57]	2022	phase II trial	>=1	Sintilimab	Anlotinib	NA	NA	NA	NA	ND	ND	NA	NA
Lheureux [58]	2022	phase II trial	>=1	Nivolumab	Cabozantinib	NA	Nivolumab	NA	NA	5.3	13	1.9	7.9
Cui [59]	2022	retrospective	2 or 3	Anlotinib	Pembrolizumab	NA	NA	NA	NA	6	13.3	NA	NA
How [60]	2022	retrospective	3	Immunotherapy monotherapy	NA	NA	Immunotherapy	immuno + non-immunotherapy	NA	1.4	3	3.2	11
De Jaeghere [61]	2023	phase II	>=1	Pembrolizumab	Immunomodulators	radiotherapy	NA	NA	NA	3.6 weeks	37.4 weeks	NA	NA
Madariaga [62]	2023	phase II non-randomized	>=1	Niraparib	Dostarlimab	NA	NA	NA	NA	2.4	ND	NA	NA
Madariaga [62]	2023	phase II non-randomized	>=1	Niraparib	NA	NA	NA	NA	NA	2.5	12.5	NA	NA
Taylor [63]	2020	phase IB/II	>=1	Lenvatinib	Pembrolizumab	NA	NA	NA	NA	9.7	ND	NA	NA
Kim [64]	2022	multicenter, retrospective, cohort	>=1	Lenvatinib	Pembrolizumab	NA	NA	NA	NA	5.3	ND	NA	NA
How [65]	2021	retrospective	1–9	Pembrolizumab	Lenvatinib (recommended dose)	NA	Pembrolizumab	Lenvatinib (reduced dose)	NA	3.2	8.6	5.5	9.4
Chiba [66]	2024	retrospective	>=1	Lenvatinib	Pembrolizumab	NA	NA	NA	NA	11.6	NA	NA	NA
Makker [67]	2023	phase II	>=1	Lenvatinib	Pembrolizumab	NA	NA	NA	NA	7.4	17.7	NA	NA
Shang [68]	2024	single arm	>=1	Lenvatinib	PD-1 inhibitor	NA	NA	NA	NA	6.6	14.2	NA	NA
Tochigi [69]	2024	retrospective	>=1	Lenvatinib	Pembrolizumab	NA	NA	NA	NA	8.47	NA	NA	NA
Zammarrelli [70]	2023	retrospective	>=1	Lenvatinib	Pembrolizumab	NA	NA	NA	NA	6	18.3	NA	NA
Makker [71]	2023	phase III multicenter, open-label	>=1	Lenvatinib	Pembrolizumab	NA	Paclitaxel or	Doxorubicin	NA	7.3	18.7	3.8	11.9
Bafaloukos [72]	1999	prospective	0	Carboplatin	MTX+5-FU	Medroxyprogesterone acetate	NA	NA	NA	ND	16	NA	16
Colon-Ottero [73]	2020	phase II	0/1	Ribociclib	Letrozole	NA	NA	NA	NA	5.4	15.7	NA	NA
Covens [74]	2011	phase II	0/1	Fulvestrant (estrogen rec negative)	NA	NA	Fulvestrant (estrogen rec positive)	NA	NA	2	3	10	26
Karagol [75]	2006	retrospective	ND	ChT	NA	NA	Hormonal	NA	NA	4	11	5	16
Pautier [76]	2017	phase II randomized	0/1	Irosustat	NA	NA	Megestrol acetate	NA	NA	16.1 weeks	NR	40.1 weeks	63.4 weeks
Rose [77]	2000	phase II	0/1	Anastrozole	NA	NA	NA	NA	NA	1	6	NA	NA
Emons [78]	2013	phase II	0/1	Fulvestrant	NA	NA	NA	NA	NA	2.3	13.2	NA	NA
Emons [79]	2014	phase II	0/1	AEZS-108	NA	NA	NA	NA	NA	7	15	NA	NA
Lindemann [80]	2014	phase II	0/1	Examestan	NA	NA	NA	NA	NA	3.8	13	NA	NA
Whitney [81]	2004	phase II	0/1	Medroxyprogesteron acetate	Tamoxifen	NA	NA	NA	NA	3	13	NA	NA
Thigpen [82]	2001	phase II	0/1	Tamoxifen	NA	NA	NA	NA	NA	1.9	8.8	NA	NA
Thigpen [83]	1999	randomized	0/1	Medroxyprogesteron acetate (low dose)	NA	NA	Medroxyprogesterone acetate (high dose)	NA	NA	3.2	11.1	2.5	7
Piver [84]	1986	prospective	ND	Melphalan	5-FU	Medroxyprogesterone acetate	NA	NA	NA	5	ND	NA	NA
Covens [85]	1996	phase II	0/1	Leuprolide	NA	NA	NA	NA	NA	5	9	NA	NA
Ayoub [86]	1988	randomized	0/1	Cyclophosphamid, 5FU, Adriamycin	Tamoxifen	Medroxyprogesterone acetate	Cyclophosphamid. 5FU. Adriamycin	NA	NA	ND	14	ND	11
Pandya [87]	2001	prospective, randomized	>=1	Megestrol acetate	NA	NA	Megestrol acetate	Tamoxifen	NA	ND	12	ND	8.6
Lentz [88]	1996	prospective	0	Megestrol acetate	NA	NA	NA	NA	NA	2.5	7.6	NA	NA
Lhommé [89]	1999	phase II	ND	Triptorelin	NA	NA	NA	NA	NA	4.2	ND	NA	NA
Rendina [90]	1984	randomized, prospective	ND	Tamoxifen	NA	NA	Medroxyprogesterone acetate	NA	NA	ND	11.5	ND	16
Kulkarni [91]	2023	retrospective	>=1	Progestines	NA	NA	NA	NA	NA	6.2	23	NA	NA
Fiorica [92]	2004	phase II	0	Megestrol acetate	tamoxifen	NA	NA	NA	NA	2.7	14	NA	NA
Hoffman [93]	1989	prospective	ND	Cisplatine+Doxorubicine	Cyclophosphamid	Megestrol acetate	NA	NA	NA	17 weeks	38 weeks	NA	NA
Mileshkin [94]	2019	phase II	0/1	Anastrozole	NA	NA	NA	NA	NA	3.2	ND	NA	NA
Covens [95]	2002	phase II	0/1	Danazol	NA	NA	NA	NA	NA	1.9	14.4	NA	NA
Asbury [96]	2002	prospective	>=1	Goserelin	NA	NA	NA	NA	NA	1.9	7.3	NA	NA
Ramondetta [97]	2009	phase II	0/1	Mifepristone	NA	NA	NA	NA	NA	1.55	10.11	NA	NA
Konstantinopoulos [98]	2023	phase II	1–8	Abemaciclib	Letrozole	NA	NA	NA	NA	9.1	21.6	NA	NA
Emons [99]	2015	phase II	0/1	Temsirolimus	NA	NA	NA	NA	NA	3	21.3	NA	NA
Ray-Coquard [100]	2013	phase II	1–2	Everolimus	NA	NA	NA	NA	NA	2.8	8.1	NA	NA
Makker [101]	2016	phase II	1–2	Apitolisib	NA	NA	NA	NA	NA	3.5	15.7	NA	NA
Matulonis [102]	2015	phase II	>=1	Pilaralisib	NA	NA	NA	NA	NA	8 pts > 6 mths	ND	NA	NA
Fleming [103]	2014	phase II	0 or 1	Temsirolimus	NA	NA	Temsirolimus	Megestrol acetate/Tamoxifen	NA	5.6	13.3	4.2	9.6
Heudel [104]	2017	phase II	1	Buparlisib	NA	NA	NA	NA	NA	4.5	10.1	NA	NA
Soliman [105]	2020	phase II	0/1	Everolimus	Letrozole	Metformin	NA	NA	NA	5.7	19.6	NA	NA
Heudel [106]	2022	phase I/II randomized RCT	0/1	Vistusertib	Anastrozole	NA	Anastrozole	NA	NA	5.2	ND	1.9	ND
Oza [107]	2011	phase II	NA	Temsirolimus ChT naive	NA	NA	Temsirolimus ChT treated	NA	NA	7.33	ND	3.25	ND
Oza [108]	2015	phase II randomized	1 or 2	Ridaforolimus	NA	NA	Progrestin	ChT	NA	3.6	10	1.9	9.6
Slomovitz [109]	2015	phase II	1 or 2	Everolimus	Letrozole	NA	NA	NA	NA	3	14	NA	NA
Del Campo [110]	2016	phase II	>=1	Gedatolisib	NA	NA	PF-04691502	NA	NA	108 days	ND	ND	ND
Slomovitz [111]	2022	phase II randomized	>=1	Everolimus	Letrozole	NA	Medroxyprogesterone acetate	Tamoxifen	NA	6	31	4	17
Myers [112]	2020	phase II	>=1	MK-2206	NA	NA	NA	NA	NA	2	8.2	NA	NA
Westin [113]	2019	phase I	1 or 2	Trametinib	GSK2141795	NA	NA	NA	NA	3.1	11.5	NA	NA
Rubinstein [114]	2020	phase II	1 to 3	LY3023414	NA	NA	NA	NA	NA	2.5	9.2	NA	NA
Aghajanian [37]	2018	phase II randomized	0	Paclitaxel	Carboplatin	Temsirolimus	Paclitaxel	Carboplatin	NA	ND	ND	ND	ND
Han [115]	2023	phase II	1–2	Sapanisertib	Paclitaxel	NA	Paclitaxel	NA	NA	5.6	13.7	3.7	14.6
Santin [116]	2020	phase II	1 TO 5	Copanlisib	NA	NA	NA	NA	NA	2.8	15.2	NA	NA
Eskander [117]	2023	phase III	0–1	Pembrolizumab	Paclitaxel	Carboplatine	Paclitaxel	Carboplatine	NA	18.8	ND	8.5	ND
Mirza [118]	2023	phase III	0–1	Dostarlimab	Paclitaxel	Carboplatine	Paclitaxel	Carboplatine	NA	36.1% (at 24 mths)	71.3% (at 24 mths)	18.1% (at 24 mths)	56% (at 24 mths)
Pignata [119]	2023	phase II	0	Avelumab	Paclitaxel	Carboplatine	Paclitaxel	Carboplatine	NA	9.6	NR	9.9	27.4
Westin [120]	2024	phase III	0	Durvalumab	Paclitaxel	Carboplatine	Paclitaxel	Carboplatine	NA	10.2	NR	9.6	25.9
Colombo [121]	2024	phase III	0	Atezolizumab	Paclitaxel	Carboplatine	Paclitaxel	Carboplatine	NA	10.1	8.9	38.7	30.2
Fleming [122]	2010	phase II	>=1	Trastuzumab	NA	NA	NA	NA	NA	1.84	7.85	NA	NA
Ahn [123]	2023	phase II	>=2	Pertuzumab	Trastuzumab	NA	NA	NA	NA	3.75	14	NA	NA
Arend [124]	2023	phase II	>=1	DKN01	NA	NA	NA	NA	NA	5.5	NR	NA	NA
Arend [124]	2023	phase II	>=1	DKN01	Paclitaxel	NA	NA	NA	NA	5.4	19.1	NA	NA
Bender [125]	2015	phase II	>=1	Cediranib	NA	NA	NA	NA	NA	3.65	12.5	NA	NA
Coleman [126]	2012	phase II	1 or 2	Aflibercept	NA	NA	NA	NA	NA	2.9	14.6	NA	NA
Dizon [127]	2014	phase II	1–3	Nintedanib	NA	NA	NA	NA	NA	3.3	10.1	NA	NA
Castonguay [128]	2014	phase II	1	Sunitinib	NA	NA	NA	NA	NA	3	19.4	NA	NA
Dhani [129]	2020	phase II	1 or 2	Cabozantinib	NA	NA	NA	NA	NA	4.6	ND	NA	NA
Powell [130]	2014	phase II	1 or 2	Brivanib	NA	NA	NA	NA	NA	3.3	10.7	NA	NA
McMeekin [131]	2007	phase II	1 or 2	Thalidomid	NA	NA	NA	NA	NA	1.7	6.3	NA	NA
Ren [132]	2023	phase II	>=1	Apatinib	NA	NA	NA	NA	NA	4.4	11.7	NA	NA
Vergote [15]	2020	phase II	1	Lenvatinib	NA	NA	NA	NA	NA	5.6	10.6	NA	NA
Moore [133]	2015	phase II	1 or 2	Trebananib	NA	NA	NA	NA	NA	1.97	6.6	NA	NA
Konecny [134]	2015	phase II, non-randomized	0–1	Dovitinib (FGFR2-mut)	NA	NA	Dovitinib (FGFR2-non-mut)	NA	NA	4.1	20.2	2.7	9.3
Westermann [135]	2024	phase II	0–1	Pazopanib	NA	NA	NA	NA	NA	3.4	7.5	NA	NA
Backes [136]	2021	phase I	0–1	Paclitaxel	Lenvatinib	NA	NA	NA	NA	14	ND	NA	NA
Leslie [137]	2012	phase II	1 or 2	Lapatinib	NA	NA	NA	NA	NA	1.82	7.33	NA	NA
Leslie [138]	2013	phase II	>=1	Gefitinib	NA	NA	NA	NA	NA	1.8	7.1	NA	NA
Coleman [139]	2015	phase II	1 or 2	Selumetinib	NA	NA	NA	NA	NA	2.3	8.5	NA	NA
Dizon [140]	2009	phase II	>=1	Ixabepilone	NA	NA	NA	NA	NA	2.9	8.7	NA	NA
McMeekin [141]	2015	phase III	>=1	Ixabepilone	NA	NA	Paclitaxel	Doxorubicin	NA	3.4	10.9	4	12.3
Liu [142]	2021	phase II	>=1	Adavosertib	NA	NA	NA	NA	NA	6.1	ND	NA	NA
Atkins [143]	2023	phase II	>=1	ONC201	NA	NA	NA	NA	NA	8.9	ND	NA	NA
Rimel [144]	2024	phase II	>=1	Olaparib	Cediranib	NA	Cediranib	NA	NA	5.5	17.6	3.8	12.4
Rimel [144]	2024	phase II	>=1	Olaparib	NA	NA	NA	NA	NA	2	13.4	NA	NA

ChT: chemotherapy. ND: no data available. NA: not applicable. NR: not reached. mths: months.

## Data Availability

The datasets used or analyzed in the study are available from the corresponding author on reasonable request.

## Supplementary Materials

The following supporting information can be downloaded at: https://www.mdpi.com/article/10.3390/cancers18132091/s1.
