# Supplementary material for: Systemic Treatment Strategies Beyond Chemotherapy in Recurrent or Advanced Endometrial Cancer: A Systematic Review and Meta-Analysis
Source: Cancers (Basel). 2026 Jun 27;18(13):2091. doi: 10.3390/cancers18132091 (PMC13359539; doi:10.3390/cancers18132091)
Supplement: Supplementary file 1 [file cancers-18-02091-s001.zip › cancers-4353429-supplementary.pdf]

Title

Systemic treatment strategies beyond chemotherapy in recurrent or advanced endometrial cancer: a systematic review and meta-analysis

Authors

István Madár[1,2], Anett Szabó[1,3], Bianca Golzio Navarro Cavalcante[1], Gábor Vleskó[1,2], Péter Hegyi[1,4,5], Nándor Ács[1,2], Tamás Kói[1,6], Emma Kálovics[1], Gábor Szabó[1,2]

Affiliations:

1. Centre for Translational Medicine, Semmelweis University, 1088 Budapest, Hungary.
2. Department of Obstetrics and Gynecology, Semmelweis University, 1088 Budapest, Hungary
3. Department of Urology, Semmelweis University, 1082 Budapest, Hungary
4. Institute for Translational Medicine, Medical School, University of Pécs, 7624 Pécs, Hungary
5. Institute of Pancreatic Diseases, Semmelweis University, 1083 Budapest, Hungary
6. Stochastics Department, Budapest University of Technology and Economics, 1111 Budapest, Hungary

Corresponding author

Gábor Szabó MD, PhD

Postal address: H-1088 Budapest, Baross utca 27, Hungary

Tel.: +36 20 4554710

E-mail address: szabo.gabor6@semmelweis.hu

TABLE OF CONTENTS

Supplementary Table S1. Prisma checklist

Supplementary Table S2. Baseline characteristics part 2

Supplementary Table S3. Treatment-related adverse events (TRAE) table and narrative review

Supplementary Table S4. Risk of bias for single-arm studies assessed by MINORS tool

Supplementary Appendix S1. Detailed inclusion and exclusion criteria

Supplementary Appendix S2. Search key

Supplementary Appendix S3. Lenvatinib and Pembrolizumab qualitative analysis

Supplementary Appendix S4. Immune checkpoint inhibitors combined with other systemic therapies

Supplementary Appendix S5. Immune checkpoint inhibitor-only therapy protocols

Supplementary Appendix S6. Anti-angiogenic-agent-only therapies

Supplementary Appendix S7. PI3K/AKT/mTOR inhibitor monotherapies

Supplementary Appendix S8. PI3K/AKT/mTOR inhibitor with aromatase inhibitors

Supplementary Appendix S9. Hormonal therapies

Supplementary Appendix S10. Anti-angiogenic agents combined with chemotherapy

Supplementary Appendix S11. Other therapy protocols

Supplementary Figure S1. Risk of bias in ICI combined with chemotherapy (paclitaxel and carboplatin)

Supplementary Figure S2. GRADE

Supplementary Figure S3. Funnel plots on the RCT studies

Supplementary Table S1. Prisma checklist.

Section and topic	Item #	Checklist item	Location where item is reported
		**Title**	
Title	1	Identify the report as a systematic review.	3
		**Abstract**	
Abstract	2	See the PRISMA 2020 for Abstracts checklist.	8
		**Introduction**	
Rationale	3	Describe the rationale for the review in the context of existing knowledge.	6-7
Objectives	4	Provide an explicit statement of the objective(s) or question(s) the review addresses.	6-7
		**Methods**	
Eligibility criteria	5	Specify the inclusion and exclusion criteria for the review and how studies were grouped for the syntheses.	8-9
Information sources	6	Specify all databases, registers, websites, organizations, reference lists and other sources searched or consulted to identify studies. Specify the date when each source was last searched or consulted.	8-9
Search strategy	7	Present the full search strategies for all databases, registers, and websites, including any filters and limits used.	8-9
Selection process	8	Specify the methods used to decide whether a study met the inclusion criteria of the review, including how many reviewers screened each record and each report retrieved, whether they worked independently, and if applicable, details of automation tools used in the process.	8-9
Data collection process	9	Specify the methods used to collect data from reports, including how many reviewers collected data from each report, whether they worked independently, any processes for obtaining or confirming data from study investigators, and if applicable, details of automation tools used in the process.	8-9
Data items	10a	List and define all outcomes for which data were sought. Specify whether all results that were compatible with each outcome domain in each study were sought (e.g., for all measures, time points, analyses), and if not, the methods used to decide which results to collect.	8-9
	10b	List and define all other variables for which data were sought (e.g., participant and intervention characteristics, funding sources). Describe any assumptions made about any missing or unclear information.	8-9
Study risk of bias assessment	11	Specify the methods used to assess risk of bias in the included studies, including details of the tool(s) used, how many reviewers assessed each study and whether they worked independently, and if applicable, details of automation tools used in the process.	13
Effect measures	12	Specify for each outcome the effect measure(s) (e.g., risk ratio, mean difference) used in the synthesis or presentation of results.	8-9
Synthesis methods	13a	Describe the processes used to decide which studies were eligible for each synthesis (e.g., tabulating the study intervention characteristics and comparing against the planned groups for each synthesis).	8-9
	13b	Describe any methods required to prepare the data for presentation or synthesis, such as handling of missing summary statistics or data conversions.	8-9
	13c	Describe any methods used to tabulate or visually display results of individual studies and syntheses.	8-9
	13d	Describe any methods used to synthesize results and provide a rationale for the choice(s). If meta-analysis was performed, describe the model(s), method(s) to identify the presence and extent of statistical heterogeneity, and software package(s) used.	8-9
	13e	Describe any methods used to explore possible causes of heterogeneity among study results (e.g., subgroup analysis, meta-regression).	8-9
	13f	Describe any sensitivity analyses conducted to assess robustness of the synthesized results.	8-9
Reporting bias assessment	14	Describe any methods used to assess risk of bias due to missing results in a synthesis (arising from reporting biases).	8-9
Certainty assessment	15	Describe any methods used to assess certainty (or confidence) in the body of evidence for an outcome.	8-9
		**Results**	
Study selection	16a	Describe the results of the search and selection process, from the number of records identified in the search to the number of studies included in the review, ideally using a flow diagram.	18
	16b	Cite studies that might appear to meet the inclusion criteria, but which were excluded, and explain why they were excluded.	-

Study characteristics	17	Cite each included study and present its characteristics.	Table 1
Risk of bias in studies	18	Present assessments of risk of bias for each included study.	SM 24-27, 37
Results of individual studies	19	For all outcomes, present, for each study: (a) summary statistics for each group (where appropriate) and (b) an effect estimate and its precision (e.g., confidence/credible interval), ideally using structured tables or plots.	10-13
Results of syntheses	20a	For each synthesis, briefly summarize the characteristics and risk of bias among contributing studies.	10-13
	20b	Present results of all statistical syntheses conducted. If meta-analysis was performed, present for each the summary estimate and its precision (e.g. confidence/credible interval) and measures of statistical heterogeneity. If comparing groups, describe the direction of the effect.	10-13
	20c	Present results of all investigations of possible causes of heterogeneity among study results.	10-13
	20d	Present results of all sensitivity analyses conducted to assess the robustness of the synthesized results.	10-13
Reporting biases	21	Present assessments of risk of bias due to missing results (arising from reporting biases) for each synthesis assessed.	10-13
Certainty of evidence	22	Present assessments of certainty (or confidence) in the body of evidence for each outcome assessed.	9
**Discussion**			
Discussion	23a	Provide a general interpretation of the results in the context of other evidence.	14-17
	23b	Discuss any limitations of the evidence included in the review.	14-17
	23c	Discuss any limitations of the review processes used.	14-17
	23d	Discuss implications of the results for practice, policy, and future research.	14-17
**Other information**			
Registration and protocol	24a	Provide registration information for the review, including register name and registration number, or state that the review was not registered.	8-9
	24b	Indicate where the review protocol can be accessed, or state that a protocol was not prepared.	8-9
	24c	Describe and explain any amendments to information provided at registration or in the protocol.	8-9
Support	25	Describe sources of financial or non-financial support for the review, and the role of the funders or sponsors in the review.	2
Competing interests	26	Declare any competing interests of review authors.	2
Availability of data, code, and other materials	27	Report which of the following are publicly available and where they can be found: template data collection forms; data extracted from included studies; data used for all analyses; analytic code; any other materials used in the review.	SM

Supplementary Table S2. Baseline characteristics part 2.

Author	Year	Measurable disease defined by	Number of patients		Age	
			Intervention group	Control group	Intervention group	Control group
Lorusso [1]	2019	RECIST 1.1	53	53	65	63
Simpkins [2]	2015	RECIST 1.0	15	0	63	NA
Aghajanian1 [3]	2018	RECIST 1.1	116	462	62	61
Liao [4]	2022	ND	41	52	46,71	45,22
Rose [5]	2017	RECIST 1.1	34	0	62	NA
Kelkar [6]	2022	ND	140	25	65	69

Kelkar [7]	2023	RECIST / clinical assessment	21	103	60	60
Wright [8]	2007	RECIST	11	0	57	NA
Fader [9]	2020	RECIST 1.1	32	29	66	73
Aghajanian1 [3]	2018	RECIST 1.1	118	462	65	61
Aghajanian [10]	2011	RECIST	56	0	62	NA
Alvarez [11]	2013	RECIST	53	0	63	NA
Rubinstein 2 [12]	2021	RECIST 1.1	30	0	65	NA
Roque [13]	2015	RECIST 1.1	15	6	61	ND
O’Malley [14]	2022	RECIST 1.1	90	0	64	NA
Konstantinopoulos2 [15]	2019	RECIST 1.1	31	0	ND	NA
Colomba [16]	2023	ND	48	0	61	NA
Bellone [17]	2022	RECIST 1.1	24	0	69	NA
Antill [18]	2021	RECIST 1.1	35	36	66	68
Berton [19]	2024	RECIST 1.1	76	NA	67	NA
André [20]	2023	RECIST 1.1	141	0	63	NA
Rubinstein [21]	2023	RECIST 1.1	38	39	64	67
Post [22]	2022	RECIST 1.1	55	0	69	NA
Konstantinopoulos1 [23]	2022	RECIST 1.1	35	0	67,9	NA
Wei [24]	2022	RECIST	23	0	56	NA
Lheureux [25]	2022	RECIST 1.1	36	18	57	66
Cui [26]	2022	RECIST 1.1	56	0	62	NA
How [27]	2022	RECIST 1.1.	8	10	65	56
De Jaeghere[28]	2023	RECIST 1.1	25	NA	67	NA
Madariaga [29]	2023	RECIST 1.1	22	NA	64,5	NA
Madariaga [29]	2023	RECIST 1.1	25	NA	69	NA
Taylor [30]	2020	RECIST	23	NA	64	NA
Kim [31]	2022	RECIST 1.1	48	NA	62,5	NA
How [32]	2021	ND	16	54	64,5	66
Chiba [33]	2024	RECIST 1.1	20	NA	61	NA
Makker3 [34]	2023	RECIST	108	NA	65,1	NA
Shang [35]	2024	RECIST 1.1	6	NA	53	NA
Tochigi [36]	2024	RECIST	15	NA	66	NA

Zammarrelli [37]	2023	RECIST 1.1	43	NA	67	NA
Makker 2 [38]	2023	RECIST 1.1	411	416	64	65
Bafaloukos [39]	1999	WHO criteria	23	NA	65	NA
Colon-Ottero [40]	2020	RECIST 1.1	20	NA	64,5	NA
Covens [41]	2011	RECIST	22	31	60-69	60-69
Karagol [42]	2006	clinical	19	16	60	64
Pautier [43]	2017	RECIST 1.0	36	37	68,1	67,4
Rose [44]	2000	clinical	23	0	71-80	NA
Emons [45]	2013	ND	35	0	69,5	NA
Emons [46]	2014	RECIST	44	0	68	NA
Lindemann [47]	2014	RECIST	40	0	70	NA
Whitney [48]	2004	ND	61	0	61-70	NA
Thigpen [49]	2001	ND	68	0	70-79	NA
Thigpen [50]	1999	ND	145	154	ND	ND
Piver [51]	1986	ND	50	0	63,7	NA
Covens [52]	1996	ND	25	0	62	NA
Ayoub [53]	1988	ND	23	20	63	63
Pandya [54]	2001	ND	20	46	68	65
Lentz [55]	1996	ND	63	NA	70-79	NA
Lhommé [56]	1999	ND	25	NA	62	NA
Rendina [57]	1984	IUCC	45	48	60,6	60,4
Kulkarni [58]	2023	ND	40	NA	NA	60,6
Fiorica [59]	2004	ND	56	NA	70	NA
Hoffman [60]	1989	ND	15	NA	ND	NA
Mileshkin [61]	2019	RECIST 1.1	84	NA	68	NA
Covens [62]	2002	clinical	25	NA	70	NA
Asbury [63]	2002	Standard Gynecologic Oncology Group response and adverse effects criteria	42	NA	71	NA
Ramondetta [64]	2009	ND	13	NA	59	NA
Konstantinopoulos [65]	2023	RECIST 1.1	30	66,2	66,2	NA
Emons [66]	2015	RECIST 1.1	22	NA	63	NA
Ray-Coquard [67]	2013	RECIST 1.0	44	NA	65	NA
Makker [68]	2016	RECIST 1.1	56	NA	65,5	NA

Matulonis [69]	201 5	RECIST 1.1	67	NA	62	NA
Fleming [70]	201 4	RECIST 1.1	50	21	ND	ND
Heudel [71]	201 7	RECIST 1.0	40	NA	66- 67	NA
Soliman [72]	202 0	RECIST 1.1	54	NA	62	NA
Heudel [73]	202 2	RECIST 1.1	49	24	70,8	68,7
Oza [74]	201 1	RECIST	33	27	66	60
Oza [75]	201 5	RECIST 1.0	64	66	65,5	66
Slomovitz [76]	201 5	RECIST 1.0	38	NA	62	NA
Del Campo [77]	201 6	RECIST 1.1	40	18	67,6	63,9
Slomovitz [78]	202 2	RECIST	37	37	64	67
Myers [79]	202 0	RECIST 1.1	36	NA	62,1	NA
Westin [80]	201 9	RECIST 1.1	26	NA	62	NA
Rubinstein 3 [81]	202 0	RECIST 1.1	28	NA	65	NA
Aghajanian1 [3]	201 8	RECIST 1.1	115	462	63	61
Han [82]	202 3	RECIST 1.1	90	90	64,5	62
Santin [83]	202 0	RECIST 1.1	11	NA	68	NA
Eskander [84]	202 3	RECIST 1.1	405	408	67	66
Mirza [85]	202 3	RECIST 1.1	245	249	64	65
Pignata [86]	202 3	RECIST 1.1	63	62	66	62
Westin [87]	202 4	RECIST 1.1	238	241	64	64
Colombo [88]	202 4	RECIST 1.1	360	189	67	65
Fleming [89]	201 0	ND	33	NA	ND	NA
Ahn [90]	202 3	RECIST 1.1	28	NA	69	NA
Arend [91]	202 3	RECIST 1.1	29	NA	61,3	NA
Arend [91]	202 3	RECIST 1.1	24	NA	63,3	NA
Bender [92]	201 5	RECIST 1.1	53	NA	65,5	NA
Coleman [93]	201 2	RECIST 1.0	49	NA	64	NA
Dizon [94]	201 4	RECIST 1.1	32	NA	ND	NA
Castonguay [95]	201 4	RECIST 1.1	33	NA	65	NA
Dhani [96]	202 0	RECIST 1.1	70	NA	64	NA
Powell [97]	201 4	RECIST	45	NA	64	NA
McMeekin [98]	200 7	RECIST	24	NA	70	NA
Ren [99]	202 3	RECIST 1.1	22	NA	61	NA

Vergote [100]	2020	RECIST 1.1	133	NA	62	NA
Moore [101]	2015	RECIST 1.1	32	NA	60-69	NA
Konecny [102]	2015	RECIST 1.1	22	31	64,5	65
Westermann [103]	2024	RECIST 1.1	60	NA	67	NA
Backes [104]	2021	RECIST 1.1	7	NA	ND	NA
Leslie [105]	2012	RECIST	31	NA	ND	NA
Leslie [106]	2013	ND	29	NA	ND	NA
Coleman [107]	2015	RECIST 1.0	54	NA	62	NA
Dizon [108]	2009	RECIST	52	NA	64	NA
McMeekin [109]	2015	RECIST 1.1	248	248	64	64
Liu [110]	2021	RECIST 1.1	34	NA	70,2	NA
Atkins [111]	2023	RECIST 1.1	10	NA	61	NA
Rimel [112]	2024	RECIST 1.1	40	40	65	64,5
Rimel [112]	2024	RECIST 1.1	40	NA	69	NA

Supplementary Table S3. Treatment-related adverse events (TRAE).

Author	Year	Nr of previous ChT lines	Intervention therapy			Comparator therapy			Nr pts in IG	Nr of pts CG	Side effects Intervention group (grade ≥3)	Side effects Control group (grade ≥3)
			Component 1	Component 2	Component 3	Component 1	Component 2	Component 3				
Lorusso[1]	2019	0-1	Paclitaxel	Carboplatin	NA	Paclitaxel	Carboplatine	Bevacizumab	53	53	Grade ≥3: anemia: 6 pts (11.1%), leukopenia: 15 pts (27.8%), neutropenia: 25 pts (46.3%)	Grade ≥3: anemia: 6 pts (11.3%), leukopenia: 15 pts (28.3%), neutropenia: 30 pts (56.6%).
Simpkins [2]	2015	>=1	Paclitaxel	Carboplatin	Bevacizumab	NA	NA	NA	15	0	Leukopenia: 7 pts with grade 3 (46.7%).<br>Neutropenia: 2 pts with grade 3 (13.3%), 8 pts with grade 4 (53.3%).<br>Thrombocytopenia: 2 pts with grade 3 (13.3%), 1 pt with grade 4 (6.7%).	NA
Aghajanian1[3]	2018	0	Paclitaxel	Carboplatin	Bevacizumab	NA	NA	NA	116	NA	Any adverse event (grades ≥3): 105 pts (93.7%), neutropenia (grades ≥3): 96 pts (85.7%), anemia (grades ≥3): 28 pts (25.0%), thrombocytopenia (grades ≥3): 22 pts (19.6%)	NA
Liao[4]	2022	ND	Nivolumab	Bevacizumab	NA	Paclitaxel	Bevacizumab	NA	41	52	ND	ND
Rose[5]	2017	0/1	Paclitaxel	Carboplatin	Bevacizumab	NA	NA	NA	34	0	Grade 3/4: leukopenia: 16 pts (47.0%), neutropenia: 21 pts (61.8%), thrombocytopenia: 4 pts (11.7%). Grade 3/4 nonhematologic: 2 pts (5.8%).	NA
Kelkar[6]	2022	>=1	ChT	Bevacizumab	NA	Hormonal	NA	NA	140	25	ND	ND

Kelkar[7]	2023	>=1	ChT	Bevacizumab	NA	Immunotherapy	NA	NA	21	103	ND	ND
Wright[8]	2007	>=1	Bevacizumab	ChT	NA	NA	NA	NA	11	0	Grades ≥3: Neutropenia: 2 pts (18.2%), anemia: 1 pt (9.1%), thrombocytopenia: 2 pts (18.2%), mucositis: 1 pt (9.1%), nausea: 1 pt (9.1%), bleeding: 1 pt (9.1%)	NA
Fader[9]	2020	0-2	Carboplatin	Paclitaxel	Trastuzumab	Carboplatin	Paclitaxel	NA	32	29	Grade 3: pruritus (1 pts), neutropenia (1 pts); leukopenia (1 pts)	No differences in toxicity between the control and experimental treatment arms (P ¼ 0.49, Wilcoxon rank–sum for maximum toxicity per patient)
Aghajanian1[2]	2018	0	Carboplatin	Ixabepilone	Bevacizumab	NA	NA	NA	118	0	Neutropenia (grades ≥3): 89 pts (78.1%), anemia (grades ≥3): 40 pts (35.1%), thrombocytopenia (grades ≥3): 31 pts (27.2%) Hypertension (grades ≥3): 19 pts (16.7%), hyperglycemia (grades 3–4): 4 pts (3.5%), bleeding, non-CNS (grades ≥3): 5 pts (4.4%)	NA
Aghajanian[10]	2011	>=1	Bevacizumab	NA	NA	NA	NA	NA	56	0	Grade ≥3: Pain: 4 pts (7.7%), hypertension: 4 pts (7.7%), metabolic: 3 pts (5.8%), musculoskeletal: 3 pts (5.8%)	NA
Alvarez[11]	2013	>=1	Bevacizumab	Temsirolimus	NA	NA	NA	NA	53	0	Grade ≥3: Gastrointestinal: 18 pts (36.7%), metabolic: 17 pts (34.7%), pain: 9 pts (18.4%), infection: 7 pts (14.3%).	NA
Rubinstein [12]	2021	>=1	Bevacizumab	NA	NA	NA	NA	NA	30	0	Grade ≥3: Bowel obstruction: 3 pts (3.0%), hypertension: 10 pts (10.0%)	NA

Roque[13]	2015	>=1	Bevacizumab	Ixabepilone	NA	Ixabepilone	NA	NA	15	6	Without bevacizumab: grade ≥3: Diarrhea: 1 pt (12.5%), fatigue: 1 pt (12.5%), neuropathy: 1 pt (12.5%).	With bevacizumab: grade ≥3 Neuropathy: 1 pt (6.3%)
O' Malley[14]	2022	>=1	Pembrolizumab	NA	NA	NA	NA	NA	90	0	Grade ≥3: Rash: 1 pt (1.1%), colitis: 1 pt (1.1%), severe skin reactions: 2 pts (2.2%), type 1 diabetes mellitus: 1 pt (1.1%), hepatitis: 1 pt (1.1%), adrenal insufficiency: 1 pt (1.1%)	NA
Konstantino-	2019	>=1	Avelumab	NA	NA	NA	NA	NA	31	0	Grade ≥3: anemia: 2 pts (6.5%), sinus bradycardia: 1 pt (3.2%), hypothyroidism: 1 pt (3.2%), diarrhea: 2 pts (6.5%), myositis: 1 pt (3.2%), rash acneiform: 1 pt (3.2%)	NA
Colomba[16]	2023	>=1	ICI	NA	NA	NA	NA	NA	48	0	NA	NA
Bellone[17]	2022	0	Pembro-lizumab	NA	NA	NA	NA	NA	24	0	Grade ≥3 adverse events: anemia: 1 pt (4.2%), hyperthyroidism: 1 pt (4.2%), diarrhea: 3 pts (12.5%), fatigue: 1 pt (4.2%), alkaline phosphatase increased: 1 pt (4.2%), hyperglycemia: 4 pts (16.7%), hypokalemia: 1 pt (4.2%)	NA
Antill[18]	2021	1 to 3	Durva-lumab (dMMR)	NA	NA	Durvalumab (pMMR)	NA	NA	35	36	No grade ≥3 immune-related adverse events	Grade ≥3 immune-related adverse events (pMMR cohort): viral hepatitis: 1 pt (3%)
Berton[19]	2024	>=1	Retifan-limab	NA	NA	NA	NA	NA	76	NA	Grade ≥3 adverse events: anemia: 10 pts (13.2%), urinary tract infection: 3 pts (3.9%), abdominal pain: 2 pts (2.6%), back pain: 2 pts (2.6%), diarrhea: 1 pt (1.3%), fatigue: 1 pt (1.3%), rash: 1 pt (1.3%)	NA
André[20]	2023	>=1	Dostarlimab	NA	NA	NA	NA	NA	141	0	Grade ≥3 TRAEs: anemia: 9 pts (2.5%), alanine aminotransferase increase: 7 pts (1.9%), lipase increase: 5 pts (1.4%)<br>Grade ≥3 irAEs: 40 pts (11.0% of all patients); hypothyroidism: 25 pts (6.9%), alanine aminotransferase increase: 21 pts (5.8%), arthralgia: 17 pts (4.7%)	NA

Rubinstein[21]	2023	>=1	Durva-lumab	NA	NA	Dur-valu-mab	Tre-me-li-mu-mab	NA	38	39	Grade ≥3: anemia (n = 3, 8%), hyperglycemia (n = 2, 5%), elevated lipase (n = 2, 5%), diarrhea, elevated alanine transaminase, hyponatremia, and lymphocyte decrease (each n = 1, 3%).	Grade ≥3: colitis (n = 4,10%), hyperglycemia, elevated lipase, and lymphocyte decrease (each n = 2, 5%).
Post[22]	2022	>=1	Durva-lumab	Ola-pa-rib	NA	NA	NA	NA	55	0	Grade ≥3 adverse events: anemia: 5 pts (10%), fatigue: 2 pts (4%), renal events: 1 pt (2%), nausea: 1 pt (2%), anorexia: 1 pt (2%), hepatotoxicity: 1 pt (2%), leukopenia: 1 pt (2%).	NA
Konstantinopoulos[23]	2022	>=1	Avelumab	Talazoparib	NA	NA	NA	NA	35	0	Grade ≥3 adverse events: anemia: 16 pts (46%), platelet count decreased: 10 pts (29%), fatigue: 4 pts (11%), neutrophil count decreased: 4 pts (11%)	NA
Wei[24]	2022	>=1	Sintili-mab	An-loti-nib	NA	NA	NA	NA	23	0	Grade ≥3 adverse events: palmar–plantar erythrodysesthesia syndrome /rash: 6 pts (26.1%), neutropenia: 1 pt (4.3%), proteinuria: 1 pt (4.3%), creatinine increased: 1 pt (4.3%), hypertension: 1 pt (4.3%), myocarditis: 1 pt (4.3%), prolonged ECG QT: 1 pt (4.3%), abscess: 1 pt (4.3%), peritonitis: 1 pt (4.3%)	NA
Lheureux[25]	2022	>=1	Nivo-lumab	Ca-bo-zan-tinib	NA	Nivo-lumab	NA	NA	36	18	Grade ≥3 any: 22 pts (61%), diarrhea: 3 pts (8%), aspartate aminotransferase increased: 2 pts (6%), alanine aminotransferase increased: 3 pts (8%), hypertension: 6 pts (17%), mucositis oral: 2 pts (6%), palmar–plantar erythrodysesthesia syndrome: 2 pts (6%), anemia: 2 pts (6%)	Grade ≥3 adverse events; lymphocyte count decreased: 1 pt (6%)
Cui[26]	2022	2/3	Anloti-nib	Pembrolizumab	NA	NA	NA	NA	56	0	Grade ≥3 adverse events: hypertension: 6 pts (10.7%), fatigue: 4 pts (7.1%), hand-foot syndrome: 4 pts (7.1%), proteinuria: 2 pts (3.6%), sore throat: 2 pts (3.6%), hypothyroidism: 2 pts (3.6%)	NA
How[27]	2022	3	Immuno-therapy monothe-rapy	NA	NA	Immunotherapy	immuno / Non-immunotherapy	NA	8	10	Grade 3/4 TRAEs in 1 pt (12.5%), and irAEs in 1 pt (12.5%)	Grade 3/4 TRAEs and irAEs: 3 (30.0%), and 6 (60.0%) of 10 patients, respectively

De Jaeghere[28]	2023	>=1	Pembrolizumab	Immunomodulators	radiotherapy	NA	NA	NA	25	NA	Grade ≥3: diarrhea: 3 pts (12.0%), colitis: 3 pts (12.0%), vomiting: 1 pt (4.0%), anemia: 1 pt (4.0%), lymphopenia: 3 pts (12.0%)	NA
Madariaga[29]	2023	>=1	Niraparib	NA	NA	Dostarlimab	Niraparib	NA	25	22	Grade ≥3: anemia (24%), fatigue (16%) and thrombocytopenia (16%).	Grade ≥3: anemia (27%) and neutropenia (14%).
Taylor[30]	2020	>=1	Lenvatinib	Pembrolizumab	NA	NA	NA	NA	23	0	Not available on the EC cohort	NA
Kim[31]	2022	>=1	Lenvatinib	Pembrolizumab	NA	NA	NA	NA	48	0	Fatigue: 9 pts (18.8%), hypertension: 8 pts (16.7%), hypothyroidism: 7 pts (14.6%), GI problem: 6 pts (12.5%), hoarseness: 5 pts (10.4%), skin eruption: 4 pts (8.3%), arthritic pain: 2 pts (4.2%), bowel perforation: 1 pt (2.1%), rectovaginal fistula: 1 pt (2.1%)	NA
How[32]	2021	1-9	Pembrolizumab	Lenvatinib	NA	Pembrolizumab	Lenvatinib (reduced dose)	NA	16	54	NA	NA
Chiba[33]	2024	>=1	Lenvatinib	Pembrolizumab	NA	NA	NA	NA	20	NA	Hypertension: 5 pts (25.0%), fatigue: 4 pts (20.0%), diarrhea (colitis): 3 pts (15.0%), stomatitis: 3 pts (15.0%), neutrophil count decreased: 2 pts (10.0%), rash: 2 pts (10.0%), anemia: 2 pts (10.0%), AST/ALT increased: 2 pts (10.0%), CPK increased: 1 pt (5.0%), adrenal insufficiency: 1 pt (5.0%), myocarditis: 1 pt (5.0%)	NA
Makker[34]	2023	>=1	Lenvatinib	Pembrolizumab	NA	NA	NA	NA	108	NA	Grade ≥4 treatment-related TEAEs occurred in 10 (9.3%) patients.	NA

Shang[35]	2024	>=1	Lenvatinib	PD-1 inhibitor	NA	NA	NA	NA	6	NA	Grade ≥3: hypertension: 3 pts (50.0%), oral mucositis: 1 pt (16.7%)	NA
Tochigi[36]	2024	>=1	Lenvatinib	Pembrolizumab	NA	NA	NA	NA	15	NA	Grade ≥3: hypertension: 6 pts (40.0%), liver dysfunction: 4 pts (26.6%), fatigue: 4 pts (26.6%), loss of appetite/anorexia: 4 pts (26.6%), proteinuria: 4 pts (26.6%), thrombocytopenia: 2 pts (13.3%), hand-foot syndrome: 2 pts (13.3%), electrolyte imbalance: 2 pts (13.3%)	NA
Zammarrelli [37]	2023	>=1	Lenva-tinib	Pembrolizumab	NA	NA	NA	NA	43	NA	Grade ≥3 adverse events: hypertension: 21 pts (49%), anemia: 11 pts (26%), fatigue: 6 pts (14%), thrombocytopenia: 5 pts (12%), diarrhea: 3 pts (7%), oral mucositis: 3 pts (7%), hypokalemia: 4 pts (9%), colitis: 2 pts (5%), arthralgia: 2 pts (5%), rash: 1 pt (2%), myalgia: 1 pt (2%).	NA
Makker2[38]	2023	>=1	Lenva-tinib	Pembrolizumab	NA	Paclitaxel **or**	Doxorubicin	NA	411	416	Leukopenia: 7 pts with grade 3 (46.7%). Neutropenia: 2 pts with grade 3 (13.3%), 8 pts with grade 4 (53.3%). Thrombocytopenia: 2 pts with grade 3 (13.3%), 1 pt with grade 4 (6.7%).	NA
Bafaloukos[39]	1999	0	Carbo-platin	MTX+5-FU	Medroxyprogesterone acetate	NA	NA	NA	23	0	Grade ≥3 adverse events: neutropenia: 3 pts, anemia: 3 pts, thrombocytopenia: 2 pts, alopecia: 2 pts, nausea–vomiting: 1 pt	NA
Colon-Otero[40]	2020	0/1	Ribociclib	Letrozole	NA	NA	NA	NA	20	0	Grade 3 or worse: leucopenia (23%), lymphocytopenia (23%), neutropenia (15%), fatigue (13%), dehydration (8%), abnormal liver function tests (8%) and acute kidney injury (8%). Grade 3 or higher neutropenia in this trial (6 pts- 15%)	NA

Covens[41]	2011	0 / 1	Fulve-strant	NA	NA	NA	NA	NA	22	31	Grade ≥3 adverse events: anemia: 2 pts (3.8%), fatigue: 1 pt (1.9%), gastrointestinal: 1 pt (1.9%), nausea: 3 pts (5.7%), vomiting: 1 pt (1.9%), diarrhea: 1 pt (1.9%), anorexia: 2 pts (3.8%), metabolic: 2 pts (3.8%), neurologic: 2 pts (3.8%), depression: 1 pt (1.9%), pain: 1 pt (1.9%), dyspnea: 1 pt (1.9%), thrombosis/embolism: 4 pts (7.6%)	NA
Karagol[42]	2006	ND	ChT	NA	NA	Hormonal	NA	NA	19	16	Chemotherapy-related grade ≥3 toxic events: anemia was seen in four (21%) patients, leukopenia was seen in four (21%) patients, noncompliance was seen in one (5%) patient, and alopecia was seen in one patient (5%). The median duration of hormonal therapy was 6 mo. Grade ≥3 toxic events were not seen in patients who received hormonal therapy.	NA
Pautier[43]	2017	0 / 1	Irosustat	NA	NA	Megestrol acetate	NA	NA	36	37	Grade 3 or higher TRAEs in the megestrol acetate arm included pulmonary embolism (n = 2) and hyperglycemia (n = 1).	Grade 3 or higher TRAEs in the irosustat arm included dry skin, asthenia, hyponatremia, and hypertension (all n = 1)
Rose[44]	2000	0 / 1	Anastro-zole	NA	NA	NA	NA	NA	23	0	Anastrozole was well-tolerated in this trial. Although one patient developed a venous thrombosis, no other grade 3 or 4 toxicities occurred.	NA
Emons[45]	2013	0 / 1	Fulvestrant	NA	NA	NA	NA	NA	35	0	Grade ≥3 adverse events: abdominal pain: 1 pt (2.9%), anorexia: 1 pt (2.9%), ascites: 1 pt (2.9%), aspiration: 1 pt (2.9%), bone pain: 1 pt (2.9%), dyspnea: 1 pt (2.9%), gastroenteritis: 1 pt (2.9%), general physical health deterioration: 1 pt (2.9%), hydronephrosis: 1 pt (2.9%), hypertension: 1 pt (2.9%), ileus: 1 pt (2.9%), nausea: 1 pt (2.9%), edema peripheral: 1 pt (2.9%), pain in extremity: 1 pt (2.9%), paresis: 1 pt (2.9%), pulmonary embolism: 1 pt (2.9%), renal failure: 1 pt (2.9%), tachycardia: 1 pt (2.9%), vomiting: 1 pt (2.9%), vulvovaginal mycotic infection: 1 pt (2.9%)	NA
Emons[46]	2014	0 / 1	AEZS-108	NA	NA	NA	NA	NA	44	0	Grade ≥3 adverse events: diarrhea: 1 pt (2.3%), fatigue: 1 pt (2.3%), leukopenia: 4 pts (9.3%), lymphopenia: 2 pts (4.7%), nausea: 1 pt (2.3%), neutropenia: 5 pts (11.7%)	NA
Lindemann[47]	2014	0 / 1	Exame-stan	NA	NA	NA	NA	NA	40	0	Grade ≥3 adverse events: anorexia: 2 pts (3.8%), nausea: 1 pt (1.9%), vomiting: 1 pt (1.9%), abdominal pain: 1 pt (1.9%), fatigue: 2 pts (3.8%), dizziness: 1 pt (1.9%), venous thrombosis: 3 pts (5.8%)	NA

Whitney[48]	2004	0 / 1	Medro-xypro-gesteron acetate	Tamoxifen	NA	NA	NA	NA	61	0	Grade ≥3 adverse events: thromboembolic episode: 1 pt, hepatic: 2 pts (1 grade 3, 1 grade 4), genitourinary (GU): 1 pt, anemia: 2 pts, hypertension: 2 pts, weight gain or loss: 2 pts	NA
Thigpen[49]	2001	0 / 1	Tamoxi-fen	NA	NA	NA	NA	NA	68	0	The most frequently reported adverse event was gastrointestinal toxicity (6%), primarily including nausea or vomiting. Two patients (3%) developed anemia, one patient experienced grade 2 thrombophlebitis, one patient had grade 1 hot flashes, and one patient experienced grade 2 fatigue. In no patient was the study treatment reported as a contributing factor in the cause of death.	NA
Thigpen[50]	1999	0 / 1	Medroxyprogesteron acetate	NA	NA	Medroxyprogesterone acetate	NA	NA	145	154	Grade ≥3 adverse events: thrombophlebitis: 6 pts (2.0%), anemia: 2 pts (0.7%), pulmonary emboli: 3 pts (1.0%)	NA
Piver[51]	1986	ND	Melphalan	5-FU	Medroxyprogesterone acetate	NA	NA	NA	50	0	NA	NA
Covens[52]	1996	0 / 1	Leuprolide	NA	NA	NA	NA	NA	25	0	NA	NA

Ayoub[53]	1988	0 / 1	Cyclophosphamid, 5FU, Adriamycin	Tamoxifen	Medroxyprogesterone acetate	Cyclophosphamid, 5FU, Adriamycin	NA	NA	23	20	NA	NA
Pandya[54]	2001	>=1	Megestrol acetate	NA	NA	Megestrol acetate	Tamoxifen	NA	20	46	Overall, mild and moderate toxicities were experienced by 40% of the patients receiving megestrol and 57% of the patients receiving the combination, of which hematologic toxicities were the most frequent. In addition, severe to life-threatening toxicities were seen in 5% of the patients on the combination arm, including one case of pulmonary embolism.	NA
Lentz[55]	1996	0	Megestrol acetate	NA	NA	NA	NA	NA	63	0	Grade ≥3 adverse events: gastrointestinal: 1 pt (1.7%), pulmonary embolus: 3 pts (5.2%), arterial thrombosis: 1 pt (1.7%), hypertension: 1 pt (1.7%), edema: 1 pt (1.7%), dyspnea/shortness of breath: 1 pt (1.7%), weight gain: 3 pts (5.2%), hyperglycemia: 3 pts (5.2%), hypercalcemia: 1 pt (1.7%), thrombocytopenia: 1 pt (1.7%), ecchymosis: 1 pt (1.7%), rash: 1 pt (1.7%)	NA
Lhommé[56]	1999	ND	Triptorelin	NA	NA	NA	NA	NA	25	0	Four grade 1 adverse events were observed for 101 injections.<br>None of the patients requested that treatment be stopped.	NA
Rendina[57]	1984	ND	Tamoxifen	NA	NA	Medroxyprogesterone acetate	NA	NA	45	48	NA	NA
Kulkarni[58]	2023	>=1	Progestins	NA	NA	NA	NA	NA	40	NA	NA	NA

Hoffman[60]	1989	ND	Cisplatine+Doxorubicine	Cyclophosphamid	Megestrol acetate	NA	NA	NA	15	0	NA	NA
Mileshkin[61]	2019	0 / 1	Anastrozole	NA	NA	NA	NA	NA	84	0	Grade ≥3 adverse events: fatigue: 7 pts (9%)	NA
Covens[62]	2002	0/1	Danazol	NA	NA	NA	NA	NA	25	0	Grade ≥3 adverse events: gastrointestinal: 2 pts (9.1%), hepatic: 2 pts (9.1%), other hematologic: 1 pt (4.5%), infection: 1 pt (4.5%), pain: 1 pt (4.5%)	NA
Asbury[63]	2002	>=1	Goserelin	NA	NA	NA	NA	NA	42	0	Grade ≥3 adverse events: gastrointestinal: 1 pt (2.3%), lymphocytopenia: 1 pt (2.3%), anemia: 1 pt (2.3%), edema: 1 pt (2.3%), weight gain/loss: 1 pt (2.3%), neurologic: 1 pt (2.3%)	NA
Ramondetta [64]	2009	0 / 1	Mifepristone	NA	NA	NA	NA	NA	13	0	Grade ≥3 adverse events: dyspnea: 3 pts (25%), fatigue: 3 pts (25%), constipation: 1 pt (8%), headache: 1 pt (8%), mood alteration: 1 pt (8%), nausea: 1 pt (8%)	NA
Konstantip-oulos[65]	2023	1-8	Abemaciclib	Letrozole	NA	NA	NA	NA	30	0	Grade ≥3 adverse events: neutrophil count decreased: 6 pts (20%), anemia: 5 pts (17%), diarrhea: 2 pts (7%), platelet count decreased: 2 pts (7%), ALT increased: 2 pts (7%), fatigue: 1 pt (3%), nausea: 1 pt (3%), AST increased: 1 pt (3%), WBC decreased: 1 pt (3%), urinary tract infection: 1 pt (3%)	NA

Emons[66]	2015	0 / 1	Temsirolimus	NA	NA	NA	NA	NA	22	0	Grade ≥3 adverse events:<br>nausea: 4 pts (9.1%), abdominal pain: 1 pt (2.3%), diarrhea: 1 pt (2.3%), stomatitis: 2 pts (4.5%), vomiting: 2 pts (4.5%), ascites: 3 pts (6.8%), subileus: 3 pts (6.8%), ileus: 1 pt (2.3%), general physical health deterioration: 2 pts (4.5%), pain: 1 pt (2.3%), rash: 1 pt (2.3%), pruritus: 1 pt (2.3%), ASAT increased: 1 pt (2.3%), ALAT increased: 1 pt (2.3%), gamma-glutamyltransferase increased: 2 pts (4.5%), blood cholesterol increased: 1 pt (2.3%), blood creatinine increased: 1 pt (2.3%), clostridium difficile colitis: 1 pt (2.3%), urosepsis: 1 pt (2.3%), pleural effusion: 1 pt (2.3%), pulmonary embolism: 2 pts (4.5%), cough: 1 pt (2.3%), pneumonitis: 1 pt (2.3%), anemia: 1 pt (2.3%), hypertriglyceridemia: 1 pt (2.3%), hypokalemia: 1 pt (2.3%), intervertebral disk protrusion: 1 pt (2.3%), renal failure: 1 pt (2.3%), abdominal cavity drainage: 1 pt (2.3%), metastases to bone: 1 pt (2.3%), tumor pain: 1 pt (2.3%)	NA
Ray-Coquard[67]	2013	1-2	Everolimus	NA	NA	NA	NA	NA	44	0	Grade ≥3 adverse events:<br>fatigue: 18 pts (42%), anorexia: 11 pts (26%), lymphopenia: 10 pts (23%), diarrhea: 5 pts (12%), thromboembolism: 5 pts (12%), infection: 7 points, anemia: 6 pts (14%), hyperglycemia: 4 pts (10%), mucositis: 4 pts (9%), vomiting: 4 pts (9%), nausea: 4 pts (9%), hypercholesterolemia: 3 pts (8%), cutaneous rash: 2 pts (5%), leucopenia: 2 pts (5%), thrombocytopenia: 2 pts (5%), pneumonitis: 2 pts (5%), constipation: 1 pt (2%), edema: 1 pt (2%), dyspnea: 1 pt (2%), neutropenia: 1 pt (2%)	NA
Makker[68]	2016	1-2	Apitolisib	NA	NA	NA	NA	NA	56	0	Treatment-related grade ≥3 AEs reported in 79% (44/56) of patients included hyperglycemia<br>(23/56, 41%), diarrhea (11/56, 20%), and rash (11/56, 20%) (Table 2). A total of 59%<br>(33/56) of patients experienced at least one serious AE (SAE) during the study, of which<br>32% (18/56) were treatment-related	NA
Matulonis[69]	2015	>=1	Pilaralisib	NA	NA	NA	NA	NA	67	0	Grade ≥3 adverse events:<br>rash: 6 pts (9.0%), alanine aminotransferase increased: 3 pts (4.5%), diarrhea: 3 pts (4.5%), aspartate aminotransferase increased: 2 pts (3.0%), fatigue: 2 pts (3.0%), nausea: 2 pts (3.0%), hyperglycemia: 1 pt (1.5%), decreased appetite: 1 pt (1.5%), abdominal pain: 1 pt (1.5%), vomiting: 1 pt (1.5%), blood alkaline phosphatase increased: 1 pt (1.5%)	NA

Flemming[70]	2014	0 or 1	Temsirolimus	NA	NA	Temsirolimus	Megestrol acetate / Tamoxifen	NA	50	21	Hemoglobin decreased: 8 pts (16.0%), hyperglycemia: 5 pts (10.0%), nausea: 4 pts (8.0%), vomiting: 4 pts (8.0%), infection: 4 pts (8.0%), fatigue: 3 pts (6.0%), edema: 2 pts (4.0%), anorexia: 3 pts (6.0%), ulceration: 1 pt (2.0%), diarrhea: 1 pt (2.0%), hypomagnesemia: 2 pts (4.0%), hypocalcemia: 2 pts (4.0%), hypercholesterolemia: 1 pt (2.0%), hypertriglyceridemia: 1 pt (2.0%), pulmonary AE: 3 pts (6.0%) *(including 1 pt with grade 5)*, vascular event: 3 pts (6.0%), cardiac infarction: 1 pt (2.0%), LV dysfunction: 1 pt (2.0%)	Platelets decreased: 1 pt (4.8%), LV dysfunction: 1 pt (4.8%), fatigue: 2 pts (9.5%), weight loss: 1 pt (4.8%), nausea: 1 pt (4.8%), vomiting: 1 pt (4.8%), hyperglycemia: 1 pt (4.8%), blood alkaline phosphatase increased: 1 pt (4.8%), vascular events: 6 pts (28.6%) *(4 grade 3, 2 grade 4)*, sudden death: 1 pt (4.8%)
Heudel[71]	2017	1	Buparlisib	NA	NA	NA	NA	NA	40	0	Grade ≥3 adverse events (n = 24): cutaneous rash: 3 pts (13%), hypertension: 4 pts (17%), abdominal pain: 2 pts (8%), fatigue: 2 pts (8%), anorexia: 1 pt (4%), mucositis: 1 pt (4%), dyspnea: 1 pt (4%), infection without neutropenia: 1 pt (4%), intestinal perforation: 1 pt (4%), change in the general state: 2 pts (8%), lymphangitis: 1 pt (4%). hepatic cytolysis (ALT/AST): 4 pts (17%), hyperglycemia: 4 pts (17%), gamma-glutamyl transferase (GGT) increased: 3 pts (13%), anemia: 1 pt (4%), lymphopenia: 1 pt (4%), hypokalemia/hypomagnesaemia/hyponatremia: 1 pt (4%), renal toxicity: 1 pt (4%)	NA
Soliman[72]	2020	0 / 1	Evero-limus	Letrozole	Metformin	NA	NA	NA	54	0	Grade ≥3 adverse events (n = 59): anemia: 14 pts (24%), hypertriglyceridemia: 8 pts (15%), hyperglycemia: 5 pts (9%), hyponatremia: 4 pts (7%), fatigue: 3 pts (6%), thrombocytopenia: 3 pts (6%), abdominal pain: 2 pts (4%), mucositis oral: 2 pts (4%), infection: 2 pts (4%), elevated liver function test: 2 pts (4%), pain: 2 pts (4%)	NA
Heudel[73]	2022	0 / 1	Vistu-sertib	Anastrozole	NA	Anastrozole	NA	NA	49	24	Grade ≥3 adverse events – Vistusertib + anastrozole arm (n = 49): decrease in lymphocyte count: 10 pts (20%), hyperglycemia: 6 pts (12%), fatigue: 4 pts (8%), anemia: 2 pts (4%), nausea: 1 pt (2%), vomiting: 1 pt (2%), diarrhea: 1 pt (2%)	Grade ≥3 adverse events – Anastrozole alone arm (n = 24): decrease in lymphocyte count: 2 pts (8%).
Oza[74]	2011	NA	Temsirolimus CN	NA	NA	Temsirolimus CT	NA	NA	33	27	Grade ≥3 adverse events – Group A (n = 33): fatigue: 4 pts (12%), diarrhea: 2 pts (6%), anorexia: 1 pt (3%), nausea: 1 pt (3%), vomiting: 1 pt (3%), mucositis (oral): 0 pts (0%), pneumonitis: 2 pts (6%), neutropenia: 1 pt (3%), thrombocytopenia: 13 pts (39%), anemia: 7 pts (21%), creatinine increase: 1 pt (3%), hypokalemia: 1 pt (3%), AST increase: 3 pts (9%)	Grade ≥3 adverse events – Group B (n = 27): fatigue: 3 pts (11%), diarrhea: 3 pts (11%), anorexia: 1 pt (4%), nausea: 1 pt (4%), vomiting: 0 pts (0%), mucositis (oral): 1 pt (4%), pneumonitis: 3 pts (11%), neutropenia: 0 pts (0%), thrombocytopenia: 11 pts (41%), anemia: 10 pts (37%), creatinine increase: 0 pts (0%), hypokalemia: 1 pt (4%), AST increase: 1 pt (4%)
Oza[75]	2015	1 or 2	Ridafo-rolimus	NA	NA	Proge-stin	ChT	NA	64	66	Grade ≥3 adverse events – Ridaforolimus arm (n = 63): hyperglycemia: 12 pts (19.0%), anemia: 8 pts (12.7%), diarrhea: 7 pts (11.1%), asthenia: 5 pts (7.9%), stomatitis: 4 pts (6.3%), mucosal inflammation: 3 pts (4.8%), anorexia: 3 pts (4.8%), fatigue: 3 pts (4.8%), abdominal pain: 2 pts (3.2%), vomiting: 2 pts (3.2%), hypercholesterolemia: 2 pts (3.2%), nausea: 1 pt (1.6%)	Grade ≥3 adverse events – Comparator arm (n = 63): abdominal pain: 3 pts (4.6%), anemia: 3 pts (4.6%), nausea: 2 pts (3.1%), fatigue: 1 pt (1.5%), anorexia: 1 pt (1.5%), pyrexia: 1 pt (1.5%), diarrhea: 1 pt (1.5%)

Slomovitz[76]	2015	1 or 2	Evero-limus	Let-roz-ole	NA	NA	NA	NA	38	0	Grade ≥3 adverse events:<br>fatigue: 4 pts (11%), hemoglobin decreased: 2 pts (5%), blood glucose increased: 2 pts (5%), ALT increased: 2 pts (5%), serum triglyceride increased: 2 pts (5%), platelet count decreased: 2 pts (5%), headache: 2 pts (5%), diarrhea: 2 pts (5%), pain (other): 1 pt (3%), pain in extremity: 1 pt (3%), dry mouth: 1 pt (3%), dyspnea: 1 pt (3%), enteritis: 1 pt (3%), hypertension: 1 pt (3%), insomnia: 1 pt (3%), musculoskeletal (other): 1 pt (3%), myalgia: 1 pt (3%), mucositis oral: 1 pt (3%), paranasal sinus infection: 1 pt (3%), pharyngolaryngeal pain: 1 pt (3%), peripheral sensory neuropathy: 1 pt (3%), pneumonia: 1 pt (3%), serum cholesterol increased: 1 pt (3%), serum potassium decreased: 1 pt (3%), vomiting: 1 pt (3%)	NA
Del Campo[77]	2016	>=1	Gedato-lisib	NA	NA	PF-04691502	NA	NA	40	18	Grade ≥3 adverse events:<br>Gedatolisib (putative PI3K basal): 4 pts (19%)<br>Gedatolisib (putative PI3K activated): 9 pts (47%)<br>Gedatolisib (putative PI3K activated + basal): 13 pts (33%)	PF-502 (putative PI3K basal): 5 pts (83%)<br>PF-502 (putative PI3K activated): 11 pts (92%)<br>PF-502 (putative PI3K activated + basal): 16 pts (89%)
Slomovitz[78]	2022	>=1	Evero-limus	Letrozole	NA	Medroxyprogesterone acetate	Tamoxifen	NA	37	37	Common grade 3 adverse events were anemia (9 [24%] patients EL vs 2 [6%] MT) and mucositis (2 [5%] vs 0 [0%]).<br>Grade 3/4 thromboembolic events were observed with MT but not with EL (0 [0%] vs 4 [11%]).	Common grade 3 adverse events were anemia (9 [24%] patients Everolimus-Letrozole (EL) vs 2 [6%] Medroxyprogesteron-Tamoxifen(MT)) and mucositis (2 [5%] vs 0 [0%]). Grade 3/4 thromboembolic events were observed with MT but not with EL (0 [0%] vs 4 [11%]).
Myers[79]	2020	>=1	MK-2206	NA	NA	NA	NA	NA	36	0	Rash maculo-papular: 7 pts (19.4%), hyperglycemia: 2 pts (5.6%), erythema multiforme: 2 pts (5.6%), thromboembolic event: 2 pts (5.6%), acute kidney injury: 1 pt (2.8%), vaginal perforation: 1 pt (2.8%), hypotension: 1 pt (2.8%)	NA
Westin[80]	2019	1 or 2	Trametinib	GSK2141795	NA	NA	NA	NA	26	0	Grade ≥3 adverse events: high dose (n = 14):<br>gastrointestinal disorders: 3 pts (21.4%), skin and subcutaneous tissue disorders: 3 pts (21.4%), metabolism and nutrition disorders: 3 pts (21.4%), vascular disorders: 3 pts (21.4%), blood and lymphatic system disorders: 2 pts (14.3%), general disorders and administration site conditions: 1 pt (7.1%), immune system disorders: 1 pt (7.1%), nervous system disorders: 1 pt (7.1%), renal and urinary disorders: 1 pt (7.1%)<br><br>Low dose (n = 12): gastrointestinal disorders: 2 pts (16.7%), general disorders and administration site conditions: 2 pts (16.7%), skin and subcutaneous tissue disorders: 1 pt (8.3%), blood and lymphatic system disorders: 1 pt (8.3%)	NA

Rubinstein [81]	2020	1 to 3	LY3023414	NA	NA	NA	NA	NA	28	0	Grade ≥3 adverse events:<br><br>hypophosphatemia: 6 pts (21%), decreased lymphocytes: 10 pts (36%), anemia: 5 pts (18%), hypokalemia: 5 pts (18%), hyperglycemia: 4 pts (14%), hyponatremia: 4 pts (14%), decreased platelets: 3 pts (11%), decreased white blood cells: 2 pts (7%), nausea: 2 pts (7%), hypocalcemia: 2 pts (7%), increased ALT: 1 pt (4%), increased ALP: 1 pt (4%), increased AST: 1 pt (4%), hypoalbuminemia: 1 pt (4%)	NA
Aghajanian1 [3]	2018	0	Paclitaxel	Carboplatin	Temsirolimus	NA	NA	NA	115	0	Grade ≥3 adverse events (PC + temsirolimus): neutropenia: 90 pts (79.6%), anemia: 35 pts (31.0%), thrombocytopenia: 31 pts (27.4%), rash: 19 pts (16.8%), hyperglycemia: 16 pts (14.1%), venous thromboembolic event: 11 pts (9.7%), mucositis oral: 7 pts (6.2%), pneumonitis: 7 pts (6.2%), neuropathy: 4 pts (3.5%), febrile neutropenia: 4 pts (3.5%), hypertension: 3 pts (2.7%), non-CNS bleeding: 1 pt (0.9%).	NA
Han[82]	2023	1-2	Sapanisertib	Paclitaxel	NA	Paclitaxel	NA	NA	90	90	Nausea: 3 pts (3.5%), diarrhea: 8 pts (9.3%), vomiting: 2 pts (2.3%), stomatitis: 1 pt (1.2%), abdominal pain: 1 pt (1.2%), anemia: 18 pts (20.9%), neutropenia: 10 pts (11.6%), leukopenia: 8 pts (9.3%) fatigue: 10 pts (11.6%), asthenia: 1 pt (1.2%), general physical health deterioration: 4 pts (4.7%), hypophosphatemia: 10 pts (11.6%), hyperglycemia: 2 pts (2.3%), decreased appetite: 2 pts (2.3%), dehydration: 2 pts (2.3%)	Nausea: 5 pts (12.2%), diarrhea: 1 pt (2.4%), vomiting: 4 pts (9.8%), stomatitis: 6 pts (14.6%), abdominal pain: 1 pt (2.4%), anemia: 1 pt (2.4%), fatigue: 6 pts (14.6%), asthenia: 3 pts (7.3%), general physical health deterioration: 1 pt (2.4%), hypophosphatemia: 1 pt (2.4%), hyperglycemia: 6 pts (14.6%), decreased appetite: 4 pts (9.8%), dehydration: 1 pt (2.4%)
Santin[83]	2020	1 TO 5	Copanlisib	NA	NA	NA	NA	NA	11	0	Grade ≥3 adverse events: overall highest grade: 9 pts (81.8%), metabolism and nutrition disorders: 5 pts (45.5%), general disorders and administration site conditions: 2 pts (18.2%), nervous system disorders: 2 pts (18.2%), blood and lymphatic system disorders: 1 pt (9.1%), infections and infestations: 1 pt (9.1%), investigations: 1 pt (9.1%), skin and subcutaneous tissue disorders: 1 pt (9.1%).	NA
Eskander[84]	2023	0-1	Pembrolizumab	Paclitaxel	Carboplatine	Paclitaxel	Carboplatine	NA	405	408	Grade ≥3 adverse events – Pembrolizumab (dMMR + pMMR): anemia: 59 pts (13.8–19.3%), neutropenia: 64 pts (11.9–18.5%), fatigue: 6 pts (0.9–1.8%), peripheral sensory neuropathy: 7 pts (1.1–3.7%), nausea: 6 pts (1.1–2.8%), constipation: 2 pts (0.4–0.9%), diarrhea: 9 pts (1.4–4.6%), thrombocytopenia: 17 pts (4.3–4.6%), arthralgia: 3 pts (1.1%), dyspnea: 8 pts (1.8–2.8%), myalgia: 3 pts (0.7%), vomiting: 4 pts (0.7–1.8%), weight loss: 2 pts (0.4–0.9%), rash: 7 pts (0.9–2.2%)	Grade ≥3 adverse events – Placebo (dMMR + pMMR):<br>anemia: 36 pts (9.1–10.4%), neutropenia: 51 pts (12.0–17.0%), fatigue: 10 pts (2.6–2.8%), peripheral sensory neuropathy: 5 pts (1.8%), nausea: 4 pts (0.9–1.1%), constipation: 2 pts (0.4%), diarrhea: 4 pts (1.1%), thrombocytopenia: 9 pts (1.9–2.6%), arthralgia: 3 pts (0.7–0.9%), dyspnea: 1 pt (0.9%), myalgia: 5 pts (1.5%), vomiting: 4 pts (0.7–1.9%), weight loss: 3 pts (0.7–0.9%), rash: 3 pts (0.7–0.9%)

Mirza[85]	2023	0-1	Dostarlimab	Paclitaxel	Carboplatine	Paclitaxel	Carboplatine	NA	245	249	Grade ≥3 adverse events – Dostarlimab group: anemia: 36 pts (14.9%), neutropenia: 23 pts (9.5%), neutrophil count decreased: 20 pts (8.3%), hypertension: 17 pts (7.1%), white-cell count decreased: 16 pts (6.6%), sepsis: 8 pts (3.3%), pulmonary embolism: 6 pts (2.5%), pyrexia: 6 pts (2.5%), dyspnea: 5 pts (2.1%), muscular weakness: 5 pts (2.1%)	Grade ≥3 adverse events – Placebo group: anemia: 40 pts (16.3%), neutrophil count decreased: 34 pts (13.8%), neutropenia: 23 pts (9.3%), lymphocyte count decreased: 18 pts (7.3%), white cell count decreased: 13 pts (5.3%), asthenia: 6 pts (2.4%), pulmonary embolism: 5 pts (2.0%), urinary tract infection: 5 pts (2.0%)
Pignata[86]	2023	0	Avelumab	Paclitaxel	Carboplatine	Paclitaxel	Carboplatine	NA	63	62	Paresthesia: 2 pts (3%), peripheral sensory neuropathy: 1 pt (2%), stroke: 1 pt (2%), vasovagal reaction: 1 pt (2%), urinary tract obstruction: 1 pt (2%), pelvic pain: 1 pt (2%), reproductive disorder (other): 1 pt (2%), vaginal hemorrhage: 1 pt (2%), dyspnea: 1 pt (2%), erythema multiforme: 1 pt (2%), pruritus: 1 pt (2%), rash: 1 pt (2%), hot flashes: 1 pt (2%), hypertension: 10 pts (16%), thromboembolic event: 1 pt (2%)	Paresthesia: 1 pt (2%), peripheral sensory neuropathy: 2 pts (3%), stroke: 1 pt (2%), uterine hemorrhage: 1 pt (2%), hypertension: 3 pts (5%)
Westin[87]	2024	0	Durvalumab	Paclitaxel	Carboplatine	Paclitaxel	Carboplatine	NA	238	241	Neutropenia: 51 pts (21.7%), anemia: 37 pts (15.7%), thrombocytopenia: 16 pts (6.8%), leukopenia: 11 pts (4.7%), fatigue/asthenia: 8 pts (3.4%)	Neutropenia 55 pts (23.3%), anemia: 34 pts (14.4%), leukopenia: 13 pts (5.5%), thrombocytopenia: 11 pts (4.7%), fatigue/asthenia: 7 pts (3.0%)
Colombo[88]	2024	0	Atezolizumab	Paclitaxel	Carboplatine	Paclitaxel	Carboplatine	NA	360	189	Neutropenia: 97 pts (27%), anemia: 49 pts (14%), thrombocytopenia: 30 pts (8%), leukopenia: 32 pts (9%), febrile neutropenia: 22 pts (6%), diarrhea: 8 pts (2%), nausea: 4 pts (1%), vomiting: 5 pts (1%), fatigue: 10 pts (3%), pyrexia: 4 pts (1%), urinary tract infection: 6 pts (2%), COVID-19: 2 pts (1%), pneumonia: 3 pts (1%), COVID-19 pneumonia: 2 pts (1%), decreased appetite: 3 pts (1%), arthralgia: 3 pts (1%), myalgia: 3 pts (1%), peripheral sensory neuropathy: 12 pts (3%), paresthesia: 2 pts (1%), hypertension: 14 pts (4%), pulmonary embolism: 11 pts (3%), myocardial infarction: 2 pts (1%), intestinal obstruction: 3 pts (1%), general physical deterioration: 1 pt (<1%), sudden death: 2 pts (1%)	Neutropenia: 51 pts (28%), anemia: 24 pts (13%), thrombocytopenia: 14 pts (8%), leukopenia: 6 pts (3%), febrile neutropenia: 7 pts (4%), diarrhea: 0 pts, nausea: 2 pts (1%), vomiting: 1 pt (1%), fatigue: 4 pts (2%), pyrexia: 1 pt (1%), urinary tract infection: 8 pts (4%), COVID-19: 0 pts, pneumonia: 1 pt (1%), decreased appetite: 1 pt (1%), arthralgia: 3 pts (2%), myalgia: 1 pt (1%), peripheral sensory neuropathy: 5 pts (3%), paresthesia: 4 pts (2%), hypertension: 6 pts (3%), pulmonary embolism: 5 pts (3%), intestinal obstruction: 4 pts (2%), hematemesis: 1 pt (1%), colon cancer: 1 pt (1%), myelodysplastic syndrome: 1 pt (1%)
Fleming[89]	2010	>=1	Trastuzumab	NA	NA	NA	NA	NA	33	0	Anemia: 2 pts (6%), other hematologic: 2 pts (6%), gastrointestinal: 3 pts (9%), metabolic: 1 pt (3%), pain: 1 pt (3%), pulmonary: 3 pts (9%), cardiovascular: 2 pts (6%)	NA

Ahn[90]	2023	>=2	Pertuzumab	Trastuzumab	NA	NA	NA	NA	28	NA	One patient experienced a grade 3 serious adverse event (muscle weakness).	NA
Arend[91]	2023	>=1	DKN01	NA	NA	NA	NA	NA	29	NA	Anemia: 5 pts (17.2%), abdominal pain: 2 pts (6.9%), nausea: 2 pts (6.9%), small intestinal obstruction: 2 pts (6.9%), vomiting: 2 pts (6.9%), acute kidney injury: 2 pts (6.9%), urinary tract obstruction: 2 pts (6.9%), ALT increased: 2 pts (6.9%), arthralgia: 2 pts (6.9%), lymphopenia: 1 pt (3.4%)	NA
Arend[91]	2023	>=1	DKN01	Paclitaxel	NA	NA	NA	NA	24	NA	Anemia: 5 pts (20.8%), abdominal pain: 2 pts (8.3%), small intestinal obstruction: 2 pts (8.3%), lymphopenia: 2 pts (8.3%), deep vein thrombosis: 2 pts (8.3%), hypertension: 2 pts (8.3%), hyponatremia: 4 pts (16.7%), hyperglycemia: 2 pts (8.3%), neutropenia: 2 pts (8.3%), ascites: 2 pts (8.3%), nausea: 1 pt (4.2%), vomiting: 1 pt (4.2%), arthralgia: 1 pt (4.2%)	NA
Bender [92]	2015	>=1	Cediranib	NA	NA	NA	NA	NA	53	0	Other investigations: 2 pts (4.2%), vomiting: 2 pts (4.2%), other gastrointestinal: 9 pts (18.8%), general and administration site: 10 pts (20.8%), metabolism/nutrition: 4 pts (8.3%), musculoskeletal/connective tissue: 2 pts (4.2%), respiratory/thoracic/mediastinal: 1 pt (2.1%), vascular disorders: 18 pts (37.5%)	NA
Coleman[93]	2012	1 or 2	Aflibercept	NA	NA	NA	NA	NA	49	0	Thrombocytopenia: 1 pt (2.3%), neutropenia: 3 pts (6.8%), anemia: 1 pt (2.3%), cardiac: 12 pts (27.3%), coagulation: 1 pt (2.3%), constitutional: 3 pts (6.8%), nausea: 3 pts (6.8%), vomiting: 2 pts (4.5%), gastrointestinal: 5 pts (11.4%), genitourinary/renal: 1 pt (2.3%), hemorrhage: 3 pts (6.8%), infection: 2 pts (4.5%), metabolic: 4 pts (9.1%), musculoskeletal: 1 pt (2.3%), other neurological: 5 pts (11.4%), pain: 8 pts (18.2%), pulmonary: 5 pts (11.4%), vascular: 1 pt (2.3%)	NA
Dizon[94]	2014	1-3	Nintedanib	NA	NA	NA	NA	NA	32	0	Nausea: 1 pt (3.1%), diarrhea: 3 pts (9.4%), other gastrointestinal: 1 pt (3.1%), ALT increased: 2 pts (6.3%), AST increased: 2 pts (6.3%), vomiting: 1 pt (3.1%), alkaline phosphatase increased: 1 pt (3.1%), vascular disorders: 1 pt (3.1%), neutropenia: 1 pt (3.1%)	NA
Castonguay [95]	2014	1	Sunitinib	NA	NA	NA	NA	NA	33	0	Leukopenia: 15%, neutropenia: 21%, anemia: 9%, lymphopenia: 12%, thrombopenia: 9%, fatigue: 45%, diarrhea: 12%, dyspepsia: 9%, nausea: 3%, hypertension: 21%, anorexia: 6%, hand–foot syndrome: 15%, vomiting: 6%, abdominal pain: 9%, ALT increase: 3%, alkaline phosphatase increase: 3%	NA

Dhani[96]	2020	1 or 2	Cabozantinib	NA	NA	NA	NA	NA	70	0	Hypertension: 26 pts (25.5%), ALT increased: 9 pts (8.8%), lymphopenia: 8 pts (7.8%), diarrhea: 8 pts (7.8%), anemia: 5 pts (4.9%), AST increased: 5 pts (4.9%), hypophosphatemia: 5 pts (4.9%), abdominal pain: 3 pts (2.9%), hypomagnesemia: 3 pts (2.9%), ALP increased: 2 pts (2.0%), thrombocytopenia: 2 pts (2.0%), fatigue: 3 pts (2.9%), weight loss: 4 pts (3.9%), nausea: 2 pts (2.0%), anorexia: 2 pts (2.0%)	NA
Powell[97]	2014	1 or 2	Brivanib	NA	NA	NA	NA	NA	45	0	Cardiac: 9 pts (20.9%), coagulation: 4 pts (9.3%), gastrointestinal: 7 pts (16.3%), nausea: 5 pts (11.6%), vomiting: 3 pts (7.0%), pain: 3 pts (7.0%), metabolic: 6 pts (14.0%), other neurological: 4 pts (9.3%), constitutional: 2 pts (4.7%), dermatologic: 1 pt (2.3%), anemia: 1 pt (2.3%), leukopenia: 1 pt (2.3%), musculoskeletal: 1 pt (2.3%), vascular: 1 pt (2.3%), death (not CTC coded): 1 pt (2.3%)	NA
McMeekin[98]	2007	1 or 2	Thalidomid	NA	NA	NA	NA	NA	24	0	Hematologic: 3 pts (12.5%), cardiovascular: 3 pts (12.5%), coagulation: 1 pt (4.2%), constitutional: 3 pts (12.5%), gastrointestinal: 2 pts (8.3%), musculoskeletal: 1 pt (4.2%), neuropathy — motor: 1 pt (4.2%), neuropathy — sensory: 1 pt (4.2%), depressed level of consciousness: 1 pt (4.2%), other neurologic: 2 pts (8.3%), pulmonary: 1 pt (4.2%)	NA
Ren[99]	2023	>=1	Apatinib	NA	NA	NA	NA	NA	22	0	Hypertension: 5 pts (15.15%), proteinuria: 2 pts (6.06%), hand-foot syndrome: 2 pts (6.06%), decreased white blood cell count: 1 pt (3.03%), diarrhea: 1 pt (3.03%)	NA
Vergote[100]	2020	1	Lenvatinib	NA	NA	NA	NA	NA	133	0	Hypertension: 41 pts (31%), fatigue/asthenia: 21 pts (16%), proteinuria: 9 pts (7%), diarrhea: 5 pts (4%), nausea/vomiting: 5 pts (4%), dehydration: 5 pts (4%), abdominal pain/upper abdominal pain: 4 pts (3%), stomatitis: 4 pts (3%), decreased weight: 3 pts (2%), palmar–plantar erythrodysesthesia syndrome: 3 pts (2%), thrombocytopenia: 2 pts (2%), decreased appetite: 2 pts (2%), headache: 2 pts (2%), increased TSH: 1 pt (1%), hypothyroidism: 1 pt (1%)	NA
Moore[101]	2015	1 or 2	Trebananib	NA	NA	NA	NA	NA	32	0	Abdominal pain: 8 pts (25%), hypertension: 4 pts (13%), nausea: 4 pts (13%), ascites: 3 pts (9%), anemia: 0 pts (0%), vomiting: 2 pts (6%), edema limbs: 2 pts (6%), hyponatremia: 3 pts (9%), hypoalbuminemia: 2 pts (6%), dyspnea: 2 pts (6%), lymphedema: 2 pts (6%), thromboembolic event: 2 pts (6%)	NA

Konecny[102]	2015	0-1	Dovitinib	NA	NA	Dovitinib	NA	NA	22	31	Diarrhea: 5 pts (9%), hypertension: 9 pts (17%), lipase increase: 4 pts (8%), pulmonary embolism: 4 pts (8%), fatigue: 4 pts (8%), skin rash: 4 pts (8%), hypertriglyceridemia: 4 pts (8%), dehydration: 3 pts (6%), thrombocytopenia: 3 pts (6%), alanine aminotransferase increase: 2 pts (4%), lymphopenia: 2 pts (4%), pain in extremity: 2 pts (4%), aspartate aminotransferase increase: 2 pts (4%), nausea: 2 pts (4%), vomiting: 2 pts (4%), γ-glutamyltransferase increase: 1 pt (2%), hyponatremia: 1 pt (2%), hypercholesterolemia: 1 pt (2%), hypomagnesaemia: 1 pt (2%), anemia: 2 pts (4%), peripheral edema: 1 pt (2%), deep vein thrombosis: 2 pts (4%), female genital tract fistula: 2 pts (4%), cerebrovascular accident: 1 pt (2%), erythema multiforme: 1 pt (2%), gastrointestinal hemorrhage: 1 pt (2%), hypovolemia: 1 pt (2%), jaundice: 1 pt (2%), pain of skin: 1 pt (2%), peripheral ischemia: 1 pt (2%), right ventricular failure: 1 pt (2%), syncope: 1 pt (2%), transaminase increased: 1 pt (2%), white blood cell count decrease: 1 pt (2%), cardiac arrest: 1 pt (2%), amylase increased: 1 pt (2%), hypokalemia: 1 pt (2%)	NA
Westerman[103]	2024	0-1	Pazopanib	NA	NA	NA	NA	NA	60	NA	Abdominal pain: 9 pts (15%), fatigue: 8 pts (13%), hypertension: 8 pts (13%), infection: 6 pts (10%), hepatic toxicity: 4 pts (7%), mucositis: 4 pts (7%), perforation: 4 pts (7%), fistula: 2 pts (3%), anemia: 2 pts (3%), diarrhea: 2 pts (3%), dehydration: 2 pts (3%), hemorrhage: 2 pts (3%), ileus: 2 pts (3%)	NA
Backes[104]	2021	0-1	Paclitaxel	Lenvatinib	NA	NA	NA	NA	7	0	Anemia: 19 pts (19%), leukopenia: 15 pts (15%), lymphopenia: 19 pts (19%), fatigue: 8 pts (8%), diarrhea: 12 pts (12%), mucositis: 4 pts (4%), nausea: 4 pts (4%), hypertension: 27 pts (27%), neutropenia: 19 pts (19%), hoarseness: 4 pts (4%), vomiting: 4 pts (4%), thrombocytopenia: 4 pts (4%), rash: 4 pts (4%), hematuria: 4 pts (4%)	NA
Leslie[105]	2012	1 or 2	Lapatinib	NA	NA	NA	NA	NA	31	0	Gastrointestinal: 6 pts (20%), cardiovascular: 1 pt (3.3%), dermatologic: 1 pt (3.3%), genitourinary/renal: 1 pt (3.3%), hemorrhage: 1 pt (3.3%), metabolic: 3 pts (10%), pulmonary: 1 pt (3.3%)	NA
Leslie[106]	2013	>=1	Gefitinib	NA	NA	NA	NA	NA	29	0	Anemia: 2 pts (7.7%), hematologic—other: 3 pts (11.5%), cardiovascular: 1 pt (3.8%), fatigue: 5 pts (19.2%), dermatologic: 4 pts (15.4%), gastrointestinal: 5 pts (19.2%), metabolic: 1 pt (3.8%), neurologic: 3 pts (11.5%), ocular: 1 pt (3.8%), pain: 4 pts (15.4%), pulmonary: 1 pt (3.8%)	NA

Coleman[107]	2015	1 or 2	Selumetinib	NA	NA	NA	NA	NA	54	0	Anemia: 5 pts (9.6%), cardiac: 3 pts (5.8%), coagulation: 1 pt (1.9%), constitutional: 8 pts (15.4%), dermatologic: 5 pts (9.6%), gastrointestinal: 6 pts (11.5%), genitourinary/renal: 1 pt (1.9%), hemorrhage: 1 pt (1.9%), hepatobiliary: 1 pt (1.9%), infection: 1 pt (1.9%), lymphatics: 4 pts (7.7%), metabolic: 6 pts (11.5%), neuropathy: 1 pt (1.9%), other neurological: 3 pts (5.8%), ocular/visual: 1 pt (1.9%), pain: 5 pts (9.6%), pulmonary: 3 pts (5.8%)	NA
Dizon[108]	2009	>=1	Ixabepilone	NA	NA	NA	NA	NA	52	0	Leukopenia: 24 pts (48%), neutropenia: 26 pts (52%), anemia: 7 pts (14%), thrombocytopenia: 2 pts (4%), cardiac: 2 pts (4%), coagulation: 1 pt (2%), constitutional: 10 pts (20%), dermatologic: 2 pts (4%), gastrointestinal: 12 pts (24%), hemorrhage: 2 pts (4%), infection: 8 pts (16%), metabolic: 4 pts (8%), neurosensory: 4 pts (8%), other neurologic: 5 pts (10%), pain: 3 pts (6%), pulmonary: 3 pts (6%), vascular: 1 pt (2%)	NA
McMeekin[[illegible]]	2015	>=1	Ixabepilone	NA	NA	Paclitaxel	Doxorubicin	NA	248	248	Grade III/IV drug-related peripheral sensory neuropathy was reported in 19 (8%; grade III, n = 17; grade IV, n = 2) patients in the ixabepilone arm, compared with only 4 (3%; all grade III in the paclitaxel arm) patients in the control arm.	Grade III/IV drug-related peripheral sensory neuropathy was reported in 19 (8%; grade III, n = 17; grade IV, n = 2) patients in the ixabepilone arm, compared with only 4 (3%; all grade III in the paclitaxel arm) patients in the control arm.
Liu[110]	2021	>=1	Adavosertib	NA	NA	NA	NA	NA	34	0	Anemia: 8 pts (23.5%), fatigue: 8 pts (23.5%), nausea: 3 pts (8.8%), platelet count decreased: 6 pts (17.6%), neutrophil count decreased: 11 pts (32.3%), vomiting: 2 pts (5.8%), ALT increased: 3 pts (8.8%), AST increased: 4 pts (11.8%), anorexia: 1 pt (2.9%), hypokalemia: 3 pts (8.8%), hypomagnesemia: 1 pt (2.9%), hyponatremia: 1 pt (2.9%), abdominal pain: 3 pts (8.8%), back pain: 1 pt (2.9%), generalized muscle weakness: 3 pts (8.8%)	NA
Atkins[111]	2023	>=1	ONC201	NA	NA	NA	NA	NA	10	NA	Lymphocyte count decreased: 1 pt, AST elevated: 1 pt, bilirubin elevated: 1 pt, stroke: 1 pt	NA
Rimel[112]	2024	>=1	Olaparib	Cediranib	NA	Cediranib	NA	NA	40	40	Fatigue: 8 pts (20.5%), hypertension: 13 pts (33.3%), anorexia: 6 pts (15%), infection: 2 pts (5.1%), nausea: 2 pts (5.1%), diarrhea: 1 pt (2.6%), vomiting: 1 pt (2.6%), abdominal pain: 1 pt (2.6%), mucositis: 1 pt (2.6%), anemia: 1 pt (2.6%), venous thromboembolism: 2 pts (5.1%), grade 5 events: 2 pts (5.1%)	Hypertension: 14 pts (36%), diarrhea: 7 pts (17.9%), fatigue: 4 pts (10%), abdominal pain: 5 pts (12.8%), infection: 4 pts (10%), anorexia: 3 pts (7.6%), vomiting: 2 pts (5.1%), anemia: 1 pt (2.6%), mucositis: 1 pt (2.6%), nausea: 1 pt (2.6%), venous thromboembolism: 1 pt (2.6%), grade 5 event: 1 pt (2.6%)
Rimel[112]	2024	>=1	Olaparib	NA	NA	NA	NA	NA	40	NA	Anemia: 5 pts (12.5%), infection: 3 pts (7.5%), nausea: 1 pt (2.6%), vomiting: 1 pt (2.6%), febrile neutropenia: 1 pt (2.5%), abdominal pain: 1 pt (2.6%), anorexia: 1 pt (2.6%), venous thromboembolism: 4 pts (10%), grade 5 event: 1 pt (2.5%)	NA

TRAE in ChT and ICI subgroups

The addition of checkpoint inhibitors does not fundamentally alter the overall pattern of severe adverse events, although some nuanced differences can be observed across the studies. In the Eskander 2023 trial investigating Pembrolizumab, rates of anemia and thrombocytopenia are somewhat higher in the immunotherapy arm, whereas neutropenia occurs at comparable frequencies between groups. Non-hematologic adverse events, including diarrhea, rash, and dyspnea, are slightly more frequent with immunotherapy, though they remain low in absolute terms. Similarly, in Mirza 2023 with Dostarlimab, hematologic toxicity profiles are broadly comparable between arms; however, immune-related or systemic events such as hypertension, sepsis, and pulmonary embolism are more evident in the immunotherapy group, while the placebo arm exhibits slightly higher rates of certain cytopenias.

In the smaller Pignata 2023 study evaluating Avelumab, interpretation is limited by sample size, yet hypertension appears notably more frequent in the immunotherapy arm (16% vs. 5%), with other severe adverse events remaining infrequent and sporadic. The Westin 2024 trial of Durvalumab demonstrates that neutropenia remains the predominant grade ≥3 adverse event in both groups (approximately 22–23%), with nearly identical incidence rates, suggesting minimal incremental toxicity attributable to the addition of durvalumab. In Colombo 2024, which evaluates Atezolizumab and represents the largest and most detailed dataset, hematologic toxicities again predominate, with neutropenia rates nearly identical between treatment arms (approximately 27–28%). However, the immunotherapy arm displays a broader range of less common severe events, including infections, thromboembolic complications, and rare cardiovascular events, likely reflecting both immune modulation and the larger sample size.

Taken together, these findings suggest that the overall toxicity burden remains primarily driven by chemotherapy and is not substantially exacerbated by the addition of checkpoint inhibitors. Hematologic toxicities consistently represent the principal grade ≥3 adverse events across all regimens. While immunotherapy introduces additional adverse events, these are generally low in frequency and include immune-related and systemic complications such as hypertension, thromboembolism, and infections. Importantly, differences between intervention and comparator arms are modest and inconsistent across trials, with no clear indication of a markedly increased risk of severe toxicity associated with the incorporation of checkpoint inhibitors.

TRAE in Lenvatinib and Pembrolizumab subgroup

Across these studies, a distinct toxicity profile emerges that differs from the chemotherapy-based regimens described previously. Rather than predominantly hematologic toxicity, the adverse event spectrum is characterized by hypertension, fatigue, gastrointestinal toxicity, and endocrine or metabolic disturbances, consistent with the known pharmacologic effects of VEGF inhibition combined with immune checkpoint blockade.

In Taylor 2020, adverse event reporting for the endometrial cancer cohort is not available, limiting interpretability. However, subsequent studies provide more granular insights. In Kim 2022, fatigue (18.8%), hypertension (16.7%), and hypothyroidism (14.6%) are the most

frequent severe adverse events, alongside gastrointestinal complications and occasional serious events such as bowel perforation and fistula formation. Similarly, Chiba 2024 reports hypertension (25%) and fatigue (20%) as leading toxicities, with additional immune-related and organ-specific events including colitis, transaminase elevations, adrenal insufficiency, and myocarditis, albeit at lower frequencies.

The How 2021 study introduces a comparison between standard and reduced-dose lenvatinib combined with pembrolizumab, suggesting an effort to mitigate toxicity, although no detailed grade ≥3 adverse event data are reported. In Makker 2023, which represents a larger cohort, grade ≥4 treatment-related adverse events occur in approximately 9.3% of patients, indicating a non-negligible burden of severe toxicity even in optimized clinical trial settings.

More recent real-world or smaller cohort studies reinforce these patterns. Shang 2024, albeit with a very limited sample size, reports a strikingly high rate of grade ≥3 hypertension (50%), while Tochigi 2024 demonstrates a broad toxicity spectrum including hypertension (40%), liver dysfunction, fatigue, anorexia, and proteinuria (each ~26.6%), reflecting both VEGF inhibitor–related and systemic effects. Zammarrelli 2023 further corroborates these findings, with hypertension affecting nearly half of patients (49%), followed by anemia, fatigue, thrombocytopenia, and gastrointestinal toxicities.

Taken together, these data suggest that in previously treated populations, the combination of lenvatinib with PD-1 inhibition is associated with a substantial and qualitatively different toxicity burden compared to chemotherapy-based regimens. Hypertension emerges as the dominant high-grade adverse event across nearly all studies, often affecting 25–50% of patients. Additionally, fatigue, gastrointestinal toxicity (including diarrhea and mucositis), hepatic dysfunction, and endocrine disturbances are consistently observed. While hematologic toxicity is still present, it is less prominent than in platinum-based chemotherapy.

TRAE in ICI-only subgroup

Across studies, severe toxicities are generally uncommon and scattered. Reported grade ≥3 events include rash, colitis, diarrhea, hepatitis or transaminase elevation, endocrinopathies such as hypothyroidism, hyperthyroidism, adrenal insufficiency, and type 1 diabetes, as well as occasional anemia or metabolic abnormalities. These events are consistent with the expected immune-related toxicity profile of checkpoint inhibitor monotherapy.

In the pembrolizumab studies, O'Malley 2022 reports low-frequency severe immune-related events, each affecting around 1–2% of patients, including rash, colitis, hepatitis, adrenal insufficiency, and type 1 diabetes. Bellone 2022 shows somewhat higher rates of diarrhea and hyperglycemia, but the cohort is small, limiting interpretation. Avelumab monotherapy in Konstantinopoulos 2019 also shows a manageable safety profile, with grade ≥3 anemia and diarrhea each reported in 6.5% of patients, alongside rare hypothyroidism, myositis, and rash.

Durvalumab monotherapy appears particularly tolerable in Antill 2021, with no grade ≥3 immune-related adverse events in the dMMR cohort and only one case of viral hepatitis in the pMMR cohort. Retifanlimab in Berton 2024 shows anemia as the most common severe event, while other toxicities such as urinary tract infection, abdominal pain, diarrhea, fatigue, and rash are infrequent. Dostarlimab in André 2023 similarly demonstrates a relatively limited grade ≥3

treatment-related toxicity burden, with anemia, ALT increase, and lipase increase reported at low rates; immune-related adverse events occur but are mostly represented by hypothyroidism, liver enzyme elevation, and arthralgia.

Overall, checkpoint inhibitor monotherapy is associated with a more favorable and manageable safety profile than combination strategies. Severe events are less frequent, and the dominant toxicity pattern is immune-related rather than chemotherapy-, PARP inhibitor-, or anti-angiogenic-driven. However, clinically important immune-mediated complications such as colitis, hepatitis, endocrinopathies, skin reactions, and metabolic events can still occur and require active surveillance.

PI3K/AKT/mTOR inhibitor monotherapies

These earlier-phase studies investigate targeted therapies acting on the PI3K/AKT/mTOR pathway, including Temsirolimus, Everolimus, Apitolisib, Buparlisib, Ridaforolimus, and Vistusertib, mostly in pretreated populations. Across studies, a consistent, mechanism-driven toxicity profile emerges.

Metabolic toxicity is particularly prominent, with hyperglycemia reported in 41% of patients treated with apitolisib and around 10–19% across several mTOR inhibitors such as ridaforolimus (19%) and temsirolimus (10%). Hyperlipidemia is also frequent; for example, hypertriglyceridemia was reported in 15% of patients treated with everolimus-based combinations. Hematologic toxicity is common, with anemia occurring in up to 24% (everolimus combinations) and thrombocytopenia reaching 39–41% in temsirolimus-treated patients in Oza 2011.

Gastrointestinal and mucosal toxicities are consistently observed, including diarrhea in up to 20% with apitolisib, as well as stomatitis and mucositis in approximately 4–9% across multiple studies. Fatigue is another frequent adverse event, affecting 42% of patients treated with everolimus in Ray-Coquard 2013, while anorexia is reported in up to 26%. Hepatic enzyme elevations are also notable, with ALT/AST increases occurring in up to 17% with buparlisib.

Some regimens show particularly high overall toxicity rates, such as 79% grade ≥3 adverse events with apitolisib. Pulmonary toxicity, including pneumonitis, is reported in around 5–11% of patients across mTOR inhibitor studies, while thromboembolic events occur in approximately 12% with everolimus.

Overall, these therapies demonstrate a predictable toxicity pattern dominated by metabolic disturbances, gastrointestinal and mucosal effects, and moderate hematologic toxicity. Although generally manageable, the relatively high frequency of grade ≥3 adverse events often requires dose modification and close monitoring, clearly distinguishing this profile from that of chemotherapy or immunotherapy.

PI3K/AKT/mTOR combined with aromatase inhibitors

In Soliman 2020, the combination of everolimus, letrozole, and metformin shows a notable burden of grade ≥3 adverse events, with anemia reported in 24%, hypertriglyceridemia in 15%, hyperglycemia in 9%, and hyponatremia in 7% of patients. Additional toxicities such as fatigue

and thrombocytopenia occur in 6%, while abdominal pain, mucositis, infection, liver enzyme elevation, and pain are each observed in 4%. This reflects a combination of metabolic, hematologic, and gastrointestinal effects.

Heudel 2022 evaluates Vistusertib plus anastrozole and demonstrates higher toxicity compared with endocrine therapy alone. In the combination arm, grade ≥3 lymphocyte decrease occurs in 20% versus 8% with anastrozole alone, while hyperglycemia is reported in 12% and fatigue in 8%. Other events, such as anemia, occur in 4%, and nausea, vomiting, and diarrhea each in 2%, again highlighting metabolic and hematologic effects.

In Slomovitz 2015, everolimus plus letrozole is associated with a broader but generally lower-frequency toxicity spectrum, with fatigue in 11% and several events—including hyperglycemia, ALT elevation, hypertriglyceridemia, thrombocytopenia, and diarrhea—each occurring in about 5%. Numerous other grade ≥3 events are reported at approximately 3%, indicating a diffuse but relatively low-incidence toxicity pattern.

Finally, Slomovitz 2022 provides a comparative perspective, showing that everolimus plus letrozole results in higher rates of anemia (24% vs 6%) and mucositis (5% vs 0%) compared with medroxyprogesterone acetate plus tamoxifen. In contrast, grade 3/4 thromboembolic events are observed only in the comparator arm (11% vs 0%), suggesting differing toxicity trade-offs between targeted therapy and hormonal treatment.

Progestins

Overall, the toxicity profile is relatively mild compared with modern targeted or combination regimens, but a consistent signal emerges for thromboembolic and metabolic events. In Pautier 2017, grade ≥3 adverse events with irosustat are rare and limited to single cases of dry skin, asthenia, hyponatremia, and hypertension (each n = 1), while the comparator Megestrol acetate arm shows pulmonary embolism (n = 2) and hyperglycemia (n = 1). Similarly, Whitney 2004 reports isolated grade ≥3 events with medroxyprogesterone acetate plus tamoxifen, including thromboembolic events (n = 1), hepatic toxicity (n = 2), anemia (n = 2), and hypertension (n = 2).

Earlier trials reinforce the prominence of thromboembolic risk. In Thigpen 1999, thrombophlebitis occurs in 2.0% and pulmonary embolism in 1.0% of patients treated with Medroxyprogesterone acetate. In Pandya 2001, severe toxicities are reported in 5% of patients receiving megestrol acetate plus tamoxifen, including a pulmonary embolism, while mild to moderate toxicities occur in 40% with megestrol alone and 57% with the combination. Lentz 1996 similarly reports pulmonary embolism in 5.2%, arterial thrombosis in 1.7%, and hyperglycemia in 5.2%, alongside other infrequent events such as hypertension, edema, and weight gain (each ~1.7–5.2%).

In contrast, targeted therapy arms within these comparisons show a different toxicity profile. In Flemming 2014, Temsirolimus is associated with anemia (16.0%), hyperglycemia (10.0%), and pulmonary or vascular events (6.0%), while the comparator hormonal arm shows a higher rate of vascular events (28.6%) and even a sudden death (4.8%). Similarly, in Slomovitz 2015, everolimus plus letrozole demonstrates a broader but generally low-frequency toxicity

pattern, with fatigue in 11% and multiple grade ≥3 events around 3–5%, including metabolic and hepatic abnormalities.

Supplementary Table S4. Risk of bias for single-arm studies assessed by MINORS tool**.**

Author (year)	A clearly stated aim	Inclusion of consecutive patients	Prospec-tive collection of data	Endpoints appropriate to the aim of the study	Unbi-ased assess-ment of the study end-point	Follow-up period appropriate to the aim of the study	Loss to follow-up less than 5%	Prospective calculation of the study size
**Lenvatinib + Pembrolizumab**								
Taylor 2020 [30]	2	2	2	1	1	1	0	2
Kim 2022 [31]	2	1	0	1	0	1	0	2
How 2021 [32]	2	1	0	2	0	2	0	2
Chiba 2024 [33]	2	1	0	1	2	2	1	2
Makker 1 2023 [34]	2	2	2	2	2	2	2	2
Shang 2024 [35]	2	2	2	1	2	2	2	2
Tochigi 2024 [36]	2	1	0	2	2	2	2	2
Zammarrelli 2023 [37]	2	1	0	2	2	2	2	2
Makker 2 2023 [38]	2	2	2	2	2	2	2	2
**Author (year)**	**A clearly stated aim**	**Inclusion of consecutive patients**	**Prospec-tive collection of data**	**Endpoints appropriate to the aim of the study**	**Unbi-ased assess-ment of the study end-point**	**Follow-up period appropriate to the aim of the study**	**Loss to follow-up less than 5%**	**Prospective calculation of the study size**
**PI3K/AKT/mTOR inhibitor monotherapies**								
Emons 2016	2	2	2	2	2	1	2	2
Makker 2016	2	2	2	2	2	1	2	2
Ray-Coquard 2013	2	2	2	2	2	1	0	2
Fleming 2014	2	2	2	2	2	1	2	2
Heudel 2017	2	2	2	0	2	1	0	2
Oza 2015	2	2	2	2	2	1	0	2

Myers 2020	2	2	2	2	2	2	2	2
**PI3K/AKT/mTOR combined with aromatase inhibitors**								
**Author (year)**	**A clearly stated aim**	**Inclusion of consecutive patients**	**Prospective collection of data**	**Endpoints appropriate to the aim of the study**	**Unbiased assessment of the study endpoint**	**Follow-up period appropriate to the aim of the study**	**Loss to follow-up less than 5%**	**Prospective calculation of the study size**
Soliman 2020	2	2	2	2	2	2	2	2
Heudel 2022	2	2	2	2	2	1	1	2
Slomovitz 2015	2	2	2	1	2	2	2	2
Slomovitz 2022	2	2	2	2	2	2	2	2
**Progestin-containing protocols**								
**Author (year)**	**A clearly stated aim**	**Inclusion of consecutive patients**	**Prospective collection of data**	**Endpoints appropriate to the aim of the study**	**Unbiased assessment of the study endpoint**	**Follow-up period appropriate to the aim of the study**	**Loss to follow-up less than 5%**	**Prospective calculation of the study size**
Flemming 2014	2	2	2	1	2	2	2	2
Pautier 2017	2	2	2	1	2	2	1	1
Whitney 2004	2	2	2	1	1	2	2	2
Thigpen 1999	2	2	2	1	1	2	2	2
Slomovitz 2022	2	2	2	1	2	2	2	2
Lentz 1996	2	2	2	1	1	2	1	2
Kulkarni 2023	2	0	0	1	0	2	1	1
Fiorica 2004	2	2	2	1	1	2	2	2
Pandya 2001	2	1	2	2	0	2	2	2

Supplementary Appendix S1. Detailed inclusion and exclusion criteria

Detailed exclusion criteria:

- Data with patients on concurrent radiotherapy was excluded.

- In cases when systemic and non-systemic prior therapy groups were included, the data with prior chemotherapy were included in the quantitative analysis.

- If data on reduced dose and standard dose groups were available, then the standard dose group was used in the quantitative analysis.

- If updated PFS and OS data were available on the same patient group, then the article with the most recent data was included.

- We excluded case reports, case series, reviews, conference abstracts, and non-English publications.

Supplementary Appendix S2. Search key

(endometr* OR uter* OR womb) AND (canc* OR carcin* OR malign* OR neoplasm*) AND

(recurren* OR advanced OR stage III OR stage 3 OR stage IV OR relapse* OR stage 4) AND

(immune checkpoint* OR monoclonal antibod* OR mAB OR immunot* OR PD1 OR PD-1 OR PDL-1 OR PDL1 OR PD-L1 OR programmed cell death* OR programmed death-ligand-1 OR programmed death ligand-1 OR programmed death ligand 1 OR nivolumab OR opdivo OR pembrolizumab OR keytruda OR cemiplimab OR libtayo OR durvalumab OR imfinzi OR atezolizumab OR tecentriq OR avelumab OR bavencio OR PARP inhibit* OR poly ADP-ribose polymerase OR poly ADP-ribose polymerase OR poly ADP-ribose polymerase OR poly ADP-ribose polymerase OR olaparib OR lynparza OR rucaparib OR rubraca OR niraparib OR zejula OR talazoparib OR talzenna OR veliparib OR abt-888 OR vascular endothelial growth factor* OR VEGF OR bevacizumab OR avastin OR HER 2 OR HER-2 OR HER2 OR human epidermal growth factor OR trastuzumab OR herceptin OR cetuximab OR erbitux OR kinase inhibitor OR levatinib OR lenvima OR nintedanib OR ofev OR ridaforolimus OR pilaralisib OR progestin OR progestogen OR levonorgestrel OR LNG OR medroxy-progesterone acetate OR MPA OR Megestrol OR hormon* OR progesteron* OR aromatase inhibitor* OR tamoxifen OR fulvestrant OR letrozole OR everolimus OR mTOR OR mechanistic target of rapamycin OR Phosphoinositide 3-kinase OR PI3K)

Supplementary Appendix S3. Lenvatinib and Pembrolizumab exploratory quantitative analysis

Seven studies [31-34,36-38] with 684 patients were included in the quantitative analysis; of note, in one study [30], only mPFS was reported. Pooled mPFS in the seven included articles was 7.03 months (95% confidence interval (CI) 5.66–8.82). Median OS was 18.02 months (95% CI 13.99–19.73). Both MMRd and MMRp patients were included. Five studies [31,33,34,36,37] were of single-arm design. The heterogeneity of the included studies was low (I2=0).

The combination of ICI with anti-angiogenesis inhibitors yielded improved survival data compared to single-agent therapy.

Risk of bias (Supplementary Table S4) as assessed by the MINORS tool was moderate to low.

Supplementary Appendix S4. Immune checkpoint inhibitors combined with other systemic therapies

The Madariaga et al. (2023) trial reported a PFS of 2.5 months and OS of 12.5 months with niraparib monotherapy, and a similar PFS of 2.4 months (OS not reached) with niraparib-dostarlimab; grade ≥3 toxicities included anemia (24–27%), thrombocytopenia (16%) and neutropenia (14%). Both treatment lines showed modest clinical activity.

Rubinstein et al. (2023) evaluated durvalumab with and without tremelimumab in patients with persistent or recurrent endometrial cancer or carcinosarcoma. In this predominantly MMR-proficient population, both single-agent and dual immune checkpoint blockade showed limited activity, failing to meet prespecified efficacy thresholds for further investigation. Post et al. (2022) conducted the DOMEC trial, which was the first to evaluate the efficacy and safety of combined PD-L1 and PARP inhibition in advanced endometrial cancer. The primary endpoint—achieving a PFS of at least 6 months in 50% of patients—was not met, with a median PFS of 3.4 months. The objective response rate was 16%, including a 2% complete response, and a subset of patients experienced durable ongoing responses. The durvalumab–olaparib combination was generally well tolerated, with treatment-related grade 3 adverse events occurring in 16% of patients and no reported grade 4 or 5 toxicities.

Wei et al. (2022) reported a median follow-up of 15.4 months (95% CI, 12.6–18.3), during which median progression-free survival was not reached, and the probability of PFS exceeding 12 months was 57.1% (95% CI 33.6–75.0). Treatment-related grade 3–4 adverse events occurred in 50.0% of patients. Overall, the combination of sintilimab and anlotinib demonstrated meaningful clinical activity with manageable toxicity in endometrial cancer. Lheureux et al. (2022) showed improved response with nivolumab plus cabozantinib, with a median PFS of 5.3 months compared to nivolumab alone (1.9 months) and grade ≥3 AEs in 31% vs 0% of participants. Cui et al. (2022), in a retrospective study on anlotinib, reported a PFS of 6 months and OS of 13.3 months. The most frequent grade ≥3 adverse events included hypertension in 6 patients (10.7%), fatigue in 4 patients (7.1%), and hand–foot syndrome in 4 patients (7.1%).

How et al. (2022) evaluated the role of immunotherapies on microsatellite-stable EC patients. Median PFS was 1.4 months (95% CI, 0.72–2.76) with immunotheapy(IO)-monotherapy, 1.4 months (95% CI, 1.0–2.8) with combination IO-only therapy, and 3.2 months (95% CI, 1.4–8.9) with IO plus non-IO therapy. Corresponding median OS durations were 3 months (95% CI, 1–4), 8 months (95% CI, 4–12), and 11 months (95% CI, 3–not reached), respectively. In the combination IO-only group, any-grade TRAEs, grade 3/4 TRAEs, and irAEs were reported in 76.5%, 5.9%, and 29.4% of patients, respectively. Overall, patients with recurrent MSS endometrial cancer treated with monotherapy or IO-only combinations experienced inferior survival outcomes compared with those receiving IO plus non-IO therapy.

Lastly, De Jaeghere et al. (2023) (PRIMMO trial) assessed a multi-agent immunomodulatory approach with pembrolizumab and reported a median PFS of 3.6 weeks and OS of 37.4 weeks, with 36% experiencing grade ≥3 AEs, notably diarrhea and colitis. In conclusion, PRIMMO failed to meet its primary endpoint; however, the combination of pembrolizumab, radiotherapy, and an immunomodulatory five-drug cocktail demonstrated modest yet durable antitumor activity with acceptable, though not negligible, toxicity.

In a subset of studies evaluating bevacizumab-based regimens, included studies demonstrated variable clinical activity and toxicity profiles. Aghajanian et al. (2011) conducted a phase II trial evaluating the activity and tolerability of single-agent bevacizumab in recurrent or persistent endometrial cancer, reporting median progression-free and overall survival of 4.2 and 10.5 months, respectively, with exploratory associations between VEGF-A levels and clinical

outcomes. Alvarez et al. (2013) found improved outcomes with bevacizumab plus temsirolimus (PFS 5.6 months, OS 16.9 months), though high-grade adverse events and treatment-related deaths were noted. Rubinstein et al. (2021) observed limited benefit from bevacizumab in heavily pretreated patients, with a 19% clinical benefit rate, concluding that bevacizumab should be considered for incorporation into novel combination strategies with immunotherapy or PARP inhibitors to potentially enhance clinical outcomes. Roque et al. (2015) showed that adding bevacizumab to ixabepilone improved activity in platinum-resistant disease, with acceptable safety. Overall, bevacizumab combinations may enhance efficacy but are associated with increased toxicity.

Supplementary Appendix S5. Immune checkpoint inhibitor-only therapy protocols

O'Malley et al. (2022) reported a median PFS of 13.1 months with pembrolizumab in Microsatellite Instability-High (MSI-H) advanced endometrial cancer patients, though OS was not reached; grade ≥3 adverse events occurred in 12%, suggesting that pembrolizumab demonstrated robust and durable antitumor activity and encouraging survival outcomes with manageable toxicity. In a similar patient cohort (MSI-H/MMRd), Berton et al. (2024) observed a median PFS of 12.2 months and a median OS of 30.2 months for retifanlimab, with grade ≥3 toxicities in 50% of patients. Bellone et al. (2022) conducted a study on 25 patients, dividing them into two groups: six patients (25%) with Lynch/Lynch-like tumors and 18 (75%) had sporadic EC. They concluded that pembrolizumab is more efficient in Lynch-like tumors (at 1 and 3 years, 100% of Lynch-like patients remained alive, while mPFS in the other group was 14.6 months).

André et al. (2023) conducted a study on MMRd solid tumors, with 141 patients in the EC subgroup. The study demonstrated a median PFS of 6 months with dostarlimab in MMRd solid tumors, including endometrial cancer, with 16.3% grade ≥3 toxicity; mOS was not reached. Antill et al. (2021) reported a median PFS of 8.3 months and that OS was not reached for MMRd patients treated with durvalumab, compared to 1.8 and 12.1 months, respectively, in MMRp patients, indicating limited efficacy in MMRp patients.

In contrast, Konstantinopoulos et al. (2019) found a lower median PFS of 4.4 months with avelumab in dMMR EC and 1.9 months in pMMR; OS data were not reported. They concluded that avelumab showed promising activity in MMRd EC regardless of PD-L1 status. Treatment-related grade 3 toxicities were reported in 19.4% of the patients. Colomba et al. (2023) retrospectively analyzed MSI-H EC treated with first-line platinum-based chemotherapy and immune checkpoint inhibitors (ICI) given as second-line treatment. They reported a median PFS of 7.8 months for the chemotherapy group, while OS was 51.9 months. After the addition of ICI as a second-line treatment, PFS was 10.7 months, and OS was 31.9 months. In the discussion, they highlighted that some patients were given ICI as first line treatment, with similar results as the ChT group. Overall, checkpoint inhibitors in dMMR/MSI-high endometrial cancer demonstrated median PFS in the range of 4-13 months, with emerging data suggesting favorable OS beyond 12 months in selected populations. Adverse events were mostly low grade, with grade ≥3 toxicity typically reported in a minority of patients. Direct comparisons between the different ICI substances and chemotherapy are lacking.

Supplementary Appendix S6. Anti-angiogenic-agent-only therapies

Across 12 phase II trials evaluating anti-angiogenic agents, efficacy and toxicity profiles were variable. Cediranib (Bender et al., 2015) achieved a median progression-free survival (PFS) of 4.8 months and overall survival (OS) of 12.5 months, with vascular disorders being the most common grade ≥3 adverse events. Similarly, cabozantinib (Dhani et al., 2020) demonstrated promising activity with a median PFS of 4.0 months at the endometrioid histologic subgroup and 4 months at the serous subgroup, suggesting that Cabozantinib has activity in serous and endometrioid histology EC. Konecny et al. (2015) grouped patients according to FGFR2 mutation status. Dovitinib treatment resulted in a PFS of 4.1 months for the FGFR2 mutation group and 2.7 months for the non-mutation group. The study did not meet its' prespecified efficacy criteria, and the observed treatment effects appeared independent of FGFR2 mutation status.

Apatinib (Ren et al., 2023) showed a PFS of 4.4 months and an OS of 11.7 months, with 27% of EC patients experiencing grade ≥3 toxicities.

Other agents such as brivanib (Powell et al., 2014) and thalidomide (McMeekin et al., 2007) were well-tolerated but had limited activity. Further agents, such as aflibercept (Coleman et al., 2012), nintedanib (Dizon et al., 2014), and sunitinib (Castonguay et al. 2014), demonstrated limited or modest activity, with median PFS mostly ranging from 2.9 to 3.5 months and significant toxicity.

Pazopanib (Westermann et al., 2024) showed modest response rates, with a median PFS of 3.4 months and OS of 7.5 months and with gastrointestinal and hypertensive toxicities being most common.

Lenvatinib after previous chemotherapy (Vergote et al., 2020) achieved a median PFS of 5.6 months and an OS of 10.6 months, a modest antitumor activity, but the treatment was generally well tolerated. Trebananib (Moore 2015) [101] demonstrated insufficient single-agent activity in recurrent endometrial cancer to justify further investigation at the evaluated dose and schedule, with a PFS of 1.9 months and an OS of 6.6 months. Overall, multikinase inhibitors such as cabozantinib and apatinib demonstrated the most favorable risk–benefit profiles, warranting further investigation. Full efficacy and safety details are provided in Supplementary Table S2 and Table 1.

Supplementary Appendix S7. PI3K/AKT/mTOR inhibitor monotherapies

For quantitative analysis, seven studies provided enough data, and all of them were phase II trials. In the PFS analysis, seven studies [66-68,70,71,75,79] with 312 patients were included. The mPFS was 3.99 months, ranging from 2.91 to 5.27 months, while mOS was reported as 11.41 months (8.7-13.8).

Supplementary Appendix S8. PI3K/AKT/mTOR inhibitor combined with aromatase inhibitors

Combining PI3K/AKT/mTOR inhibitors with endocrine therapy may help to overcome resistance and improve outcomes in hormone receptor-positive endometrial cancer.

This meta-analysis included four studies [72,73,76,78] comprising a total of 96 patients with recurrent or advanced endometrial cancer treated with a combination of mTOR inhibitors and aromatase inhibitors.

The pooled mPFS was 5.56 months (CI 95% 4.03-8.31), indicating moderate disease control with the combination therapy. The mOS was 15.89 months (CI 3.52–22.14), although survival estimates varied significantly among the included studies, reflecting heterogeneity in patient populations and treatment protocols.

With regard to TRAEs (Supplementary Table S3), included studies generally described the combination regimen as tolerable, with toxicity profiles consistent with those expected for mTOR inhibition. Overall, these studies reinforce that everolimus-based combinations are characterized by consistent rates of metabolic toxicity (hyperglycemia ~9–12%, hyperlipidemia up to 15%), hematologic effects (anemia up to 24%), and mucosal or gastrointestinal toxicity, with combination regimens generally increasing both frequency and severity compared with endocrine therapy alone.

Supplementary Appendix S9. Hormonal therapies

A total of nine studies [43,48,50,54,55,58,59,70,78] with recurrent or advanced EC were included in the quantitative analysis of progestin-containing therapy protocols.

Eight studies [43,48,50,55,58,59,70,78] including 503 patients reported data on PFS, which yielded an mPFS of 3.58 months (95% CI 2.32–5.14).

The mOS reported by all nine included articles was 12.06 months, which ranged from 8.83 to 14.63 months across the included studies, with 523 patients included.

Adverse effects related to progestin treatment were reported in one-third of patients, with the majority classified as mild. The incidence of severe toxicity was low, with thromboembolic events (approximately 1–5% across studies) being the most frequently reported severe adverse effect. Importantly, the risk of thromboembolic complications was comparable to that seen with standard first-line chemotherapy using carboplatin and paclitaxel.

The overall quality of studies was low, with the risk of bias for patient selection being moderate. Heterogeneity was also low.

Supplementary Figure S1. Risk of bias in ICI combined with chemotherapy (paclitaxel and carboplatin).

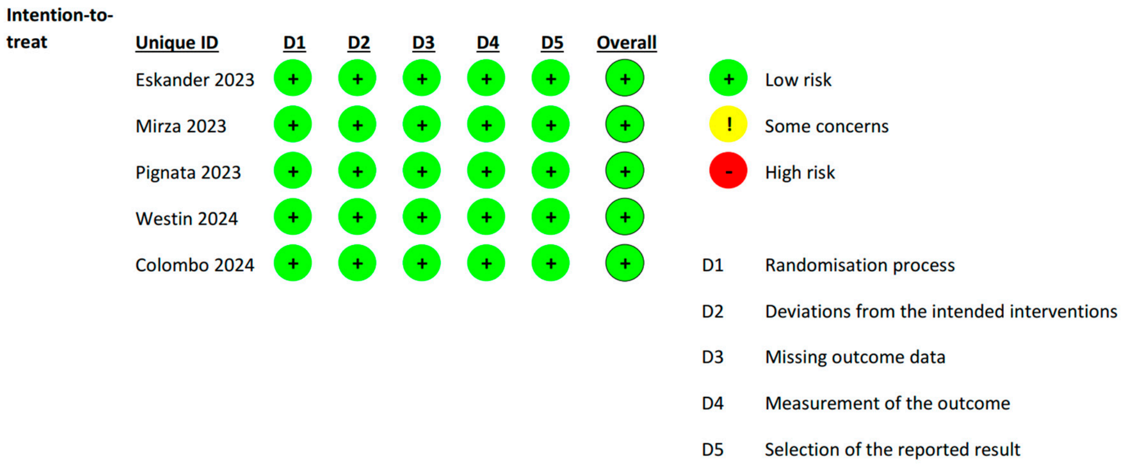

Supplementary Appendix S10. Anti-angiogenic agents combined with chemotherapy

Multiple studies evaluated the impact of bevacizumab and combination regimens in advanced or recurrent endometrial cancer. In randomized trials, the addition of bevacizumab to carboplatin–paclitaxel improved outcomes, yet not consistently. Lorusso et al. (2019) [1] reported a mPFS of 13.7 vs 10.5 months and an OS of 40 vs 29.7 months with vs without bevacizumab. Similarly, Rose et al. (2017) confirmed the additive value of the bevacizumab and ChT combination in a mixed patient cohort of first- and second-line therapy receivers: PFS was 20 months, and OS was 56 months. Simpkins et al. (2015) confirmed the activity of bevacizumab-based combinations, with a median PFS of 18 months, an OS of 58 months and manageable grade ≥3 toxicities, notably neutropenia and leukopenia.

Fader et al. (2020) showed enhanced efficacy of trastuzumab combined with chemotherapy (PFS 9.2 vs 7 months; OS 29.6 vs 24.4 months) in HER2-positive disease.

In real-world analyses, Kelkar et al. (2023) observed superior survival with immunotherapy (especially pembrolizumab) over chemotherapy ± bevacizumab (PFS 29.0 vs 4.0 months; OS 30.0 vs 7.0 months), while Kelkar et al. (2022) observed limited survival benefits in patients with non-MSI-H or pMMR treated with second-line therapy with chemotherapy ± bevacizumab or hormonal therapy.

Wright et al. (2007) provided supporting evidence of the clinical benefit of bevacizumab combined with chemotherapy, although on a small patient cohort. Liao et al. (2022) further demonstrated improved outcomes with bevacizumab combined with PD-1 inhibitors compared to paclitaxel chemotherapy (OS 33.2 vs 21.8 months) in MMRd patients.

Targeted strategies beyond anti-VEGF included lenvatinib plus weekly paclitaxel (Backes 2021), achieving a PFS of 14.0 months in endometrial cancer patients, highlighting that the combination was safe and could be suitable for patients who are not candidates for lenvatinib/pembrolizumab or other immunotherapies.

The carboplatin/paclitaxel/bevacizumab triplet reported significantly higher OS compared to the historical carboplatin–paclitaxel control arm of GOG 209 in a three-arm GOG trial in advanced/recurrent EC. No differences in PFS were reported between the three arms (carboplatin–paclitaxel–bevacizumab vs carboplatin–paclitaxel–temsirolimus vs ixabepilone/carboplatin/bevacizumab) in front of a not negligible rate of grade 3–4 toxicities, mainly hypertension (Aghajanian et al., 2018).

Supplementary Appendix S11. Other therapy possibilities.

HER2-targeted therapies were assessed in multiple trials. In an early-phase study, trastuzumab monotherapy demonstrated minimal activity, with a mPFS of 1.9 months and an OS of 6.8 months (Fleming et al., 2010). They concluded that they found no evidence of trastuzumab activity against HER2-positive endometrial cancer. More promising results emerged from a pertuzumab plus trastuzumab combination in ERBB2/3-amplified tumors, which achieved a median PFS of 3.75 months and an OS of 16 months, with manageable grade ≥3 toxicities (one patient out of 28) (Ahn eta al., 2023).

EGFR/HER2 pathway inhibitor lapatinib showed limited clinical activity in unselected patients (Leslie et at., 2012). Gefitinib, an EGFR inhibitor, yielded low clinical benefit (Leslie et al., 2013). Similarly, the MEK inhibitor selumetinib showed modest efficacy, with a median PFS of 2.3 months and OS of 8.5 months (Coleman et al. 2015).

Adavosertib, a WEE1 inhibitor, showed encouraging activity in patients with TP53-mutated serous uterine cancer tumors, achieving a median PFS of 6.1 months; however, 61.8% of patients experienced a grade 3 or higher treatment-related TRAE (Liu et al., 2021). The combination of olaparib and cediranib, targeting PARP and VEGFR, respectively, provided a median PFS of 5.5 months and an OS of 17.6 months (Rimel et al., 2024).

Microtubule-targeting agents like ixabepilone showed modest single-agent efficacy (PFS: 2.9 months (Dizon et al. 2009) and performed worse in a head-to-head comparison with paclitaxel/doxorubicin in a phase II trial (McMeekin et al., 2015), highlighting limited clinical utility.

Emerging approaches include ONC201 (inducer of tumor necrosis factor-related apoptosis-inducing ligand (TRAIL) transcription). Monotherapy did not induce objective responses in recurrent EC (Atkins et al., 2023). Additionally, DKN-01, a DKK1 inhibitor combined with paclitaxel, showed promising results, with a median PFS of 5.4 months and favorable tolerability (Arend et al., 2023).

Supplementary Figure S2. GRADE – RCTs.

Question: Chemotherapy + ICI compared to chemotherapy for recurrent or advanced endometrial cancer

Certainty assessment							№ of patients		Effect		Certainty	Importance
№ of studies	Study design	Risk of bias	Inconsistency	Indirectness	Imprecision	Other considerations	Chemotherapy + ICI	Chemotherapy	Relative (95% CI)	Absolute (95% CI)		
**Progression-free survival at allcomers**												
5	randomized trials	not serious	serious[a]	not serious	serious[b]	none	1311 participants	1149 participants	**HR 0.76** (0.65 to 0.89) [progression-free]	--	⨁⨁◯◯ Low[a,b]	IMPORTANT
							-	0.0%		--		
**Progression-free survival in MMR-deficient patients**												
5	randomized trials	not serious	not serious	not serious	not serious	none	318 participants	302 participants	**HR 0.34** (0.27 to 0.44) [progression-free]	--	⨁⨁⨁⨁ High	CRITICAL
							-	0.0%		--		
**Progression-free survival in MMR-proficient patients**												
5	randomized trials	not serious	serious[a]	not serious	serious[c]	none	978 participants	837 participants	**HR 0.77** (0.62 to 0.97) [progression-free survival]	--	⨁⨁◯◯ Low[a,c]	IMPORTANT
							-	0.0%		--		

CI: confidence interval; **HR:** hazard ratio

Explanations

a. In MMR-proficient patients treated with avelumab and chemotherapy, PFS and OS were worse than in the comparator group treated with chemotherapy. This finding is inconsistent with the findings of the other studies.

b. The prediction interval is too large for PFS in the all-comer population.

c. The prediction interval is too large for PFS in the MMR-proficient population.

GRADE – Lenvatinib and Pembrolizumab subgroup

Question: Lenvatinib and Pembrolizumab compared to none for recurrent or advanced endometrial cancer

Certainty assessment							№ of patients		Effect		Certainty	Importance
№ of studies	Study design	Risk of bias	Inconsistency	Indirectness	Imprecision	Other considerations	Lenvatinib and Pembrolizumab	none	Relative (95% CI)	Absolute (95% CI)		
**Progression-free survival**												
7	non-randomized studies	not serious	very serious[a]	serious[b]	very serious[c]	none	684 participants		not estimable	**-- per 1000** (from -- to --)		NOT IMPORTANT

Certainty assessment							№ of patients		Effect		Certainty	Importance
№ of studies	Study design	Risk of bias	Inconsistency	Indirectness	Imprecision	Other considerations	Lenvatinib and Pembrolizumab	none	Relative (95% CI)	Absolute (95% CI)		
							-	0.0%		-- per 1000 (from -- to --)	⨁◯◯◯ Very low[a,b,c]	

Overall survival

7	non-randomized studies	not serious	very serious[a]	serious[b]	very serious[c]	none	684 participants		not estimable	-- per 1000 (from -- to --)	⨁◯◯◯ Very low[a,b,c]	NOT IMPORTANT
							-	0.0%		-- per 1000 (from -- to --)		

CI: confidence interval

Explanations

a. Methodological inconsistency among the included studies

b. Inclusion of single-arm studies

c. Inclusion of individual patient data (IPD) from single-arm design studies

GRADE – PI3K/AKT/mTOR inhibitor monotherapies

Question: PI3K/AKT/mTOR inhibitor monotherapies compared to none for recurrent or advanced endometrial cancer

Certainty assessment							№ of patients		Effect		Certainty	Importance
№ of studies	Study design	Risk of bias	Inconsistency	Indirectness	Imprecision	Other considerations	PI3K/AKT/mTOR inhibitor monotherapies	none	Relative (95% CI)	Absolute (95% CI)		
**Progression-free survival**												
7	non-randomized studies	not serious	very serious[a]	serious[b]	very serious[c]		312 participants		not estimable	-- per 1000 (from -- to --)	⨁◯◯◯ Very low[a,b,c]	NOT IMPORTANT
							-	0.0%		-- per 1000 (from -- to --)		
**Overall survival**												
7	non-randomized studies	not serious	very serious[a]	serious[b]	very serious[c]		312 participants		not estimable	-- per 1000 (from -- to --)		NOT IMPORTANT

Certainty assessment							№ of patients		Effect		Certainty	Importance
№ of studies	Study design	Risk of bias	Inconsistency	Indirectness	Imprecision	Other considerations	PI3K/AKT/mTOR inhibitor monotherapies	none	Relative (95% CI)	Absolute (95% CI)		
							-	0.0%		**-- per 1000** (from -- to --)	⊕◯◯◯ Very low[a,b,c]	

CI: confidence interval

Explanations

a. Methodological inconsistency among the included studies

b. Inclusion of single-arm studies

c. inclusion of individual patient data (IPD) from single-arm design studies

GRADE – PI3K/AKT/mTOR combined with aromatase inhibitors

Question: PI3K/AKT/mTOR combined with aromatase inhibitors compared to none for recurrent or advanced endometrial cancer

Certainty assessment							№ of patients		Effect		Certainty	Importance
№ of studies	Study design	Risk of bias	Inconsistency	Indirectness	Imprecision	Other considerations	PI3K/AKT/mTOR combined with aromatase inhibitors	none	Relative (95% CI)	Absolute (95% CI)		
**Progression-free survival**												
4	non-randomized studies	not serious	very serious[a]	serious[b]	very serious[c]		96 participants		not estimable	**-- per 1000** (from -- to --)	⊕◯◯◯ Very low[a,b,c]	NOT IMPORTANT
							-	0.0%		**-- per 1000** (from -- to --)		
**Overall survival**												
4	non-randomized studies	not serious	very serious[a]	serious[b]	very serious[c]		96 participants		not estimable	**-- per 1000** (from -- to --)	⊕◯◯◯ Very low[a,b,c]	NOT IMPORTANT
							-	0.0%		**-- per 1000** (from -- to --)		

CI: confidence interval

Explanations

a. Methodological inconsistency among the included studies

b. Inclusion of single-arm studies

c. inclusion of individual patient data (IPD) from single-arm design studies

GRADE – PI3K/AKT/mTOR combined with aromatase inhibitors

Question: Progestins compared to none for recurrent or advanced endometrial cancer

Certainty assessment							№ of patients		Effect		Certainty	Importance
**№ of studies**	**Study design**	**Risk of bias**	**Inconsistency**	**Indirectness**	**Imprecision**	**Other considerations**	**Progestines**	**none**	**Relative (95% CI)**	**Absolute (95% CI)**		
**Progression-free survival**												
8	non-randomized studies	not serious	very serious[a]	serious[b]	very serious[c]		503 participants		not estimable	**-- per 1000** (from -- to --)	⊕◯◯◯ Very low[a,b,c]	NOT IMPORTANT
							-	0.0%		**-- per 1000** (from -- to --)		
**Overall survival**												
8	non-randomized studies	not serious	very serious[a]	serious[b]	very serious[c]		503 participants		not estimable	**-- per 1000** (from -- to --)	⊕◯◯◯ Very low[a,b,c]	NOT IMPORTANT
							-	0.0%		**-- per 1000** (from -- to --)		

CI: confidence interval

Explanations

a. Methodological inconsistency among the included studies

b. Inclusion of single-arm studies

c. Inclusion of individual patient data (IPD) from single-arm design studies

GRADE – Aromatase inhibitor containing therapy protocols

Question: Aromatase inhibitor containing therapy protocols compared to none for recurrent or advanced endometrial cancer

Certainty assessment							№ of patients		Effect		Certainty	Importance
**№ of studies**	**Study design**	**Risk of bias**	**Inconsistency**	**Indirectness**	**Imprecision**	**Other considerations**	**Aromatase inhibitor containing therapy protocols**	**none**	**Relative (95% CI)**	**Absolute (95% CI)**		
**Progression-free survival**												
6	non-randomized studies	not serious	very serious[a]	serious[b]	very serious[c]		268 participants		not estimable	**-- per 1000** (from -- to --)	⨁◯◯◯ Very low[a,b,c]	NOT IMPORTANT
							-	0.0%		**-- per 1000** (from -- to --)		
**Overall survival**												
4	non-randomized studies	not serious	very serious[a]	serious[b]	very serious[c]		151 participants		not estimable	**-- per 1000** (from -- to --)	⨁◯◯◯ Very low[a,b,c]	NOT IMPORTANT
							-	0.0%		**-- per 1000** (from -- to --)		

CI: confidence interval

Explanations

a. Methodological inconsistency among the included studies

b. Inclusion of single-arm studies

c. Inclusion of individual patient data (IPD) from single-arm design studies

Supplementary Figure S3. Funnel plots of the RCT studies.

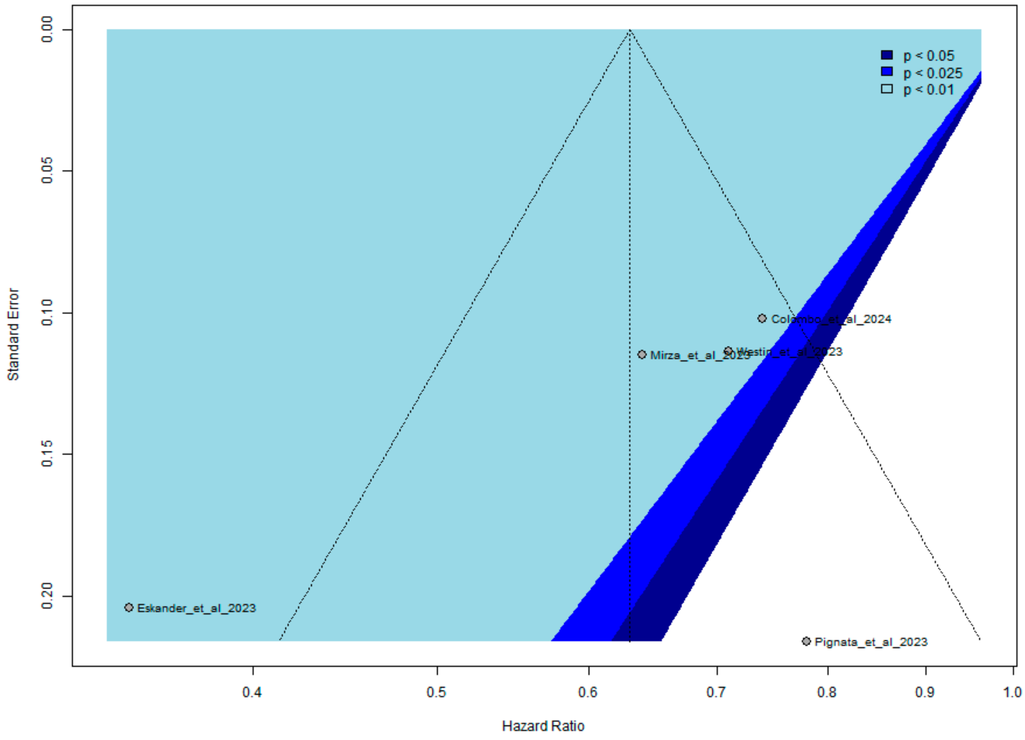

Figure S3.1. Funnel plot of PFS among allcomers.

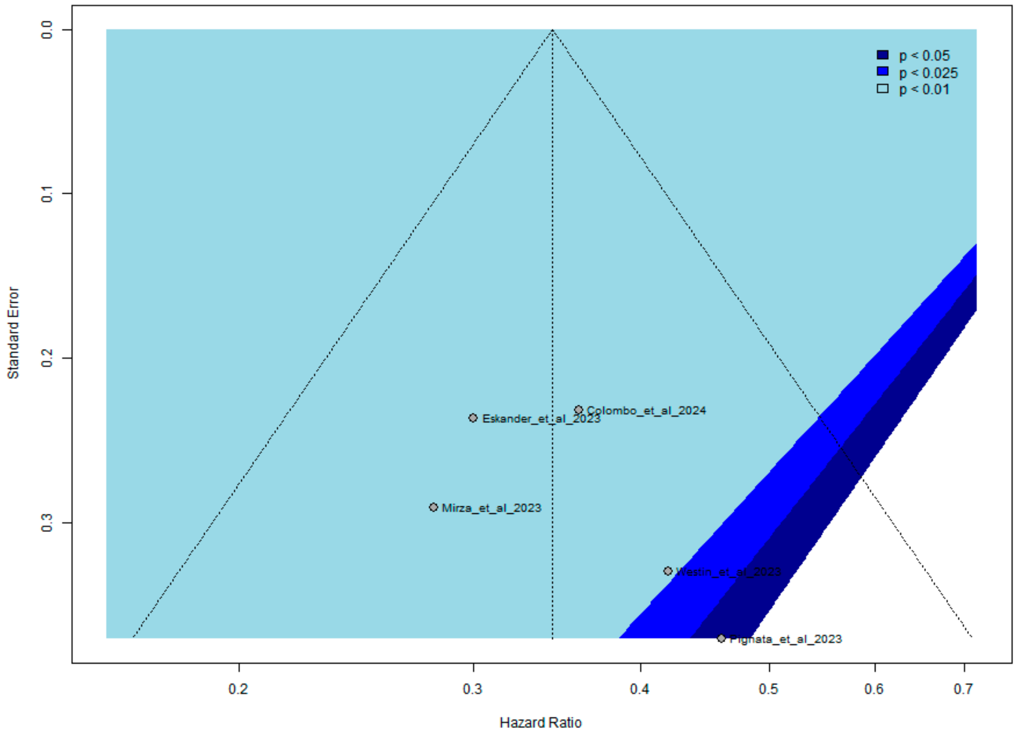

Figure S3.2. Funnel plot of PFS among dMMR group.

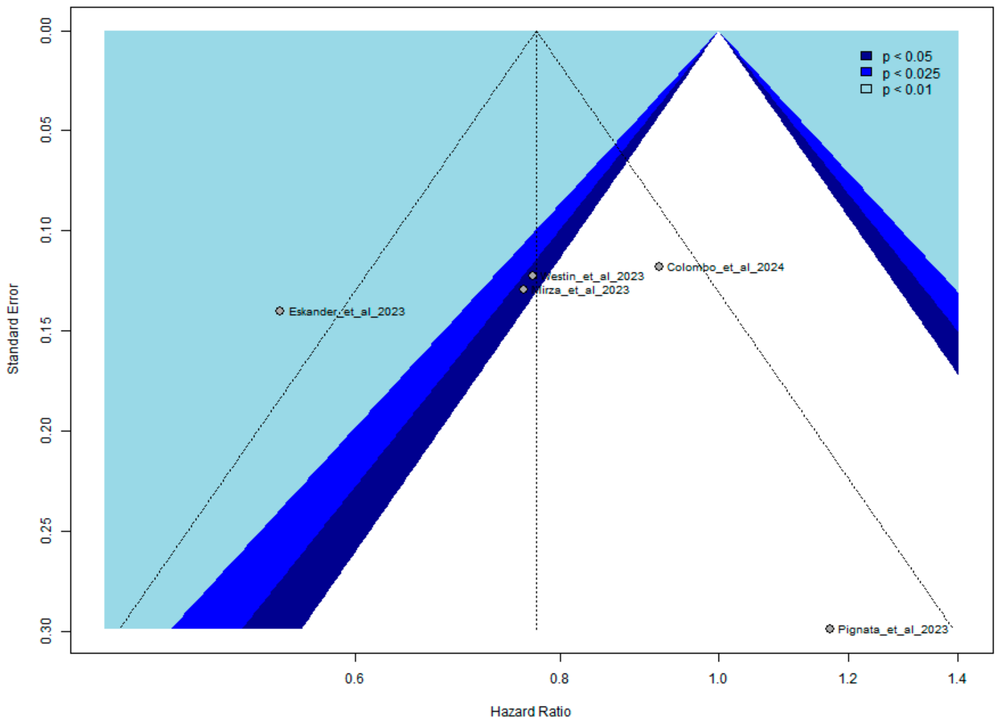

Figure S3.3. Funnel plot of PFS among pMMR group.

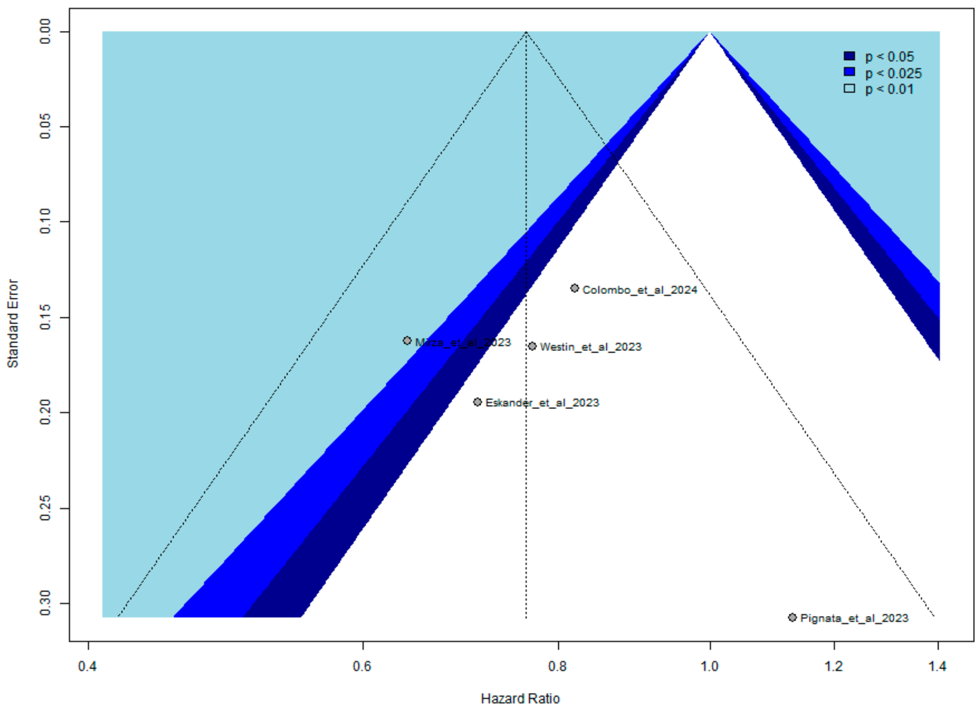

Figure S3.4. Funnel plot of OS among allcomers.

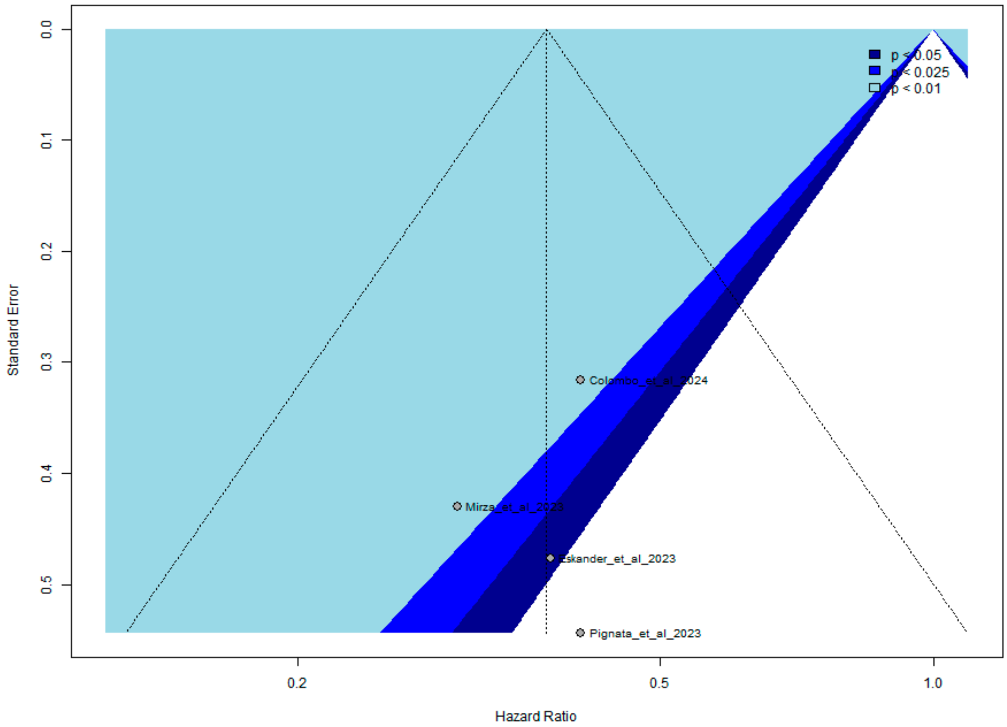

Figure S3.5. Funnel plot of OS among dMMR group.

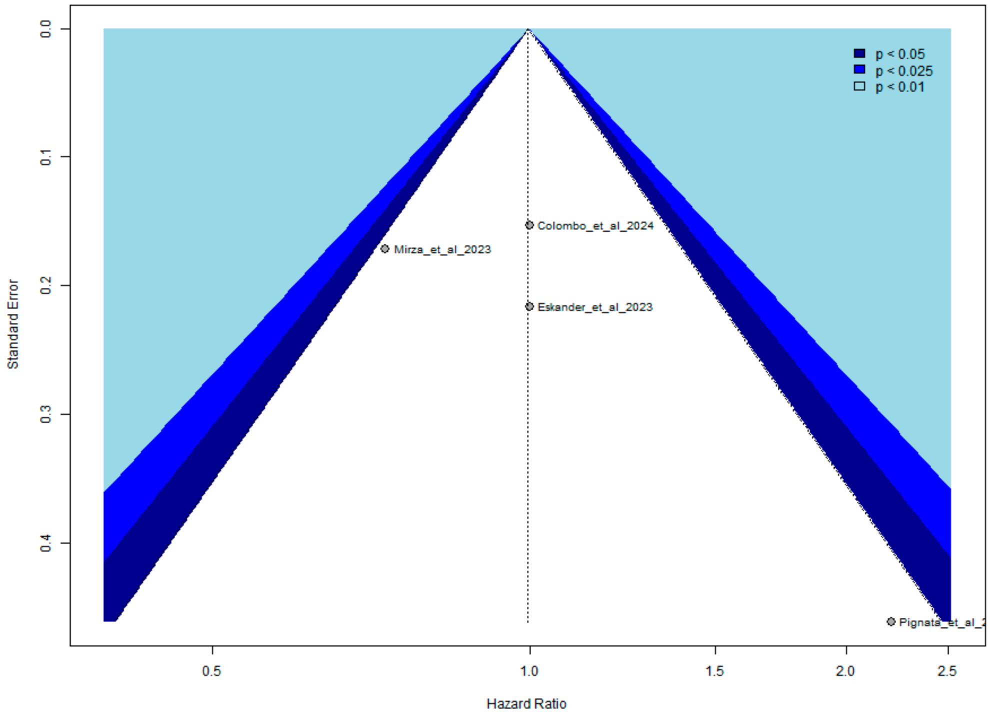

Figure S3.6. Funnel plot of OS among pMMR group.

Abbreviations: OS: overall survival, PFS: progression-free survival, pMMR: mismatch repair proficient, dMMR: mismatch repair deficient, RCT: randomized controlled trial.

References

1. Lorusso, D.; Ferrandina, G.; Colombo, N.; Pignata, S.; Pietragalla, A.; Sonetto, C.; Pisano, C.; Lapresa, M.T.; Savarese, A.; Tagliaferri, P.; et al. Carboplatin-paclitaxel compared to Carboplatin-Paclitaxel-Bevacizumab in advanced or recurrent endometrial cancer: MITO END-2 - A randomized phase II trial. *Gynecol Oncol* **2019**, *155*, 406-412, doi:10.1016/j.ygyno.2019.10.013.
2. Simpkins, F.; Drake, R.; Escobar, P.F.; Nutter, B.; Rasool, N.; Rose, P.G. A phase II trial of paclitaxel, carboplatin, and bevacizumab in advanced and recurrent endometrial carcinoma (EMCA). *Gynecol Oncol* **2015**, *136*, 240-245, doi:10.1016/j.ygyno.2014.12.004.
3. Aghajanian, C.; Filiaci, V.; Dizon, D.S.; Carlson, J.W.; Powell, M.A.; Secord, A.A.; Tewari, K.S.; Bender, D.P.; O'Malley, D.M.; Stuckey, A.; et al. A phase II study of frontline paclitaxel/carboplatin/bevacizumab, paclitaxel/carboplatin/temsirolimus, or ixabepilone/carboplatin/bevacizumab in advanced/recurrent endometrial cancer. *Gynecol Oncol* **2018**, *150*, 274-281, doi:10.1016/j.ygyno.2018.05.018.
4. Liao, Y.; Zhu, C.; Song, X.; Ruan, J.; Ding, Y.; Chen, Y.; Yang, Q. Efficacy of PD-1 Inhibitor Combined with Bevacizumab in Treatment of Advanced Endometrial Cancer Patients with Mismatch Repair Deficiency (dMMR)/High-Level Microsatellite Instability (MSI-H). *Med Sci Monit* **2022**, *28*, e934493, doi:10.12659/msm.934493.
5. Rose, P.G.; Ali, S.; Moslemi-Kebria, M.; Simpkins, F. Paclitaxel, Carboplatin, and Bevacizumab in Advanced and Recurrent Endometrial Carcinoma. *Int J Gynecol Cancer* **2017**, *27*, 452-458, doi:10.1097/igc.0000000000000891.
6. Kelkar, S.S.; Prabhu, V.S.; Zhang, J.; Corman, S.; Macahilig, C.; Rusibamayila, N.; Odak, S.; Duska, L.R. Treatment patterns and real-world clinical outcomes in patients with advanced endometrial cancer that are non-microsatellite instability high (non-MSI-high) or mismatch repair proficient (pMMR) in the United States. *Gynecol Oncol Rep* **2022**, *42*, 101026, doi:10.1016/j.gore.2022.101026.
7. Kelkar, S.S.; Prabhu, V.S.; Corman, S.; Odak, S.; Rusibamayila, N.; Macahilig, C.; Orlowski, R.; Duska, L. Treatment patterns and real-world clinical outcomes in patients with advanced endometrial cancer who are microsatellite instability (MSI)-high or are mismatch repair deficient (dMMR) in the United States. *Gynecol Oncol* **2023**, *169*, 154-163, doi:10.1016/j.ygyno.2022.10.016.
8. Wright, J.D.; Powell, M.A.; Rader, J.S.; Mutch, D.G.; Gibb, R.K. Bevacizumab therapy in patients with recurrent uterine neoplasms. *Anticancer Res* **2007**, *27*, 3525-3528.
9. Fader, A.N.; Roque, D.M.; Siegel, E.; Buza, N.; Hui, P.; Abdelghany, O.; Chambers, S.; Secord, A.A.; Havrilesky, L.; O'Malley, D.M.; et al. Randomized Phase II Trial of Carboplatin-Paclitaxel Compared with Carboplatin-Paclitaxel-Trastuzumab in Advanced (Stage III-IV) or Recurrent Uterine Serous Carcinomas that Overexpress Her2/Neu (NCT01367002): Updated Overall Survival Analysis. *Clin Cancer Res* **2020**, *26*, 3928-3935, doi:10.1158/1078-0432.Ccr-20-0953.
10. Aghajanian, C.; Sill, M.W.; Darcy, K.M.; Greer, B.; McMeekin, D.S.; Rose, P.G.; Rotmensch, J.; Barnes, M.N.; Hanjani, P.; Leslie, K.K. Phase II trial of bevacizumab in recurrent or persistent endometrial cancer: a Gynecologic Oncology Group study. *J Clin Oncol* **2011**, *29*, 2259-2265, doi:10.1200/jco.2010.32.6397.
11. Alvarez, E.A.; Brady, W.E.; Walker, J.L.; Rotmensch, J.; Zhou, X.C.; Kendrick, J.E.; Yamada, S.D.; Schilder, J.M.; Cohn, D.E.; Harrison, C.R.; et al. Phase II trial of combination bevacizumab and temsirolimus in the treatment of recurrent or persistent endometrial carcinoma: a Gynecologic Oncology Group study. *Gynecol Oncol* **2013**, *129*, 22-27, doi:10.1016/j.ygyno.2012.12.022.
12. Rubinstein, M.M.; Dickinson, S.; Narayan, P.; Zhou, Q.; Iasonos, A.; Ma, W.; Lakhman, Y.; Makker, V. Bevacizumab in advanced endometrial cancer. *Gynecol Oncol* **2021**, *161*, 720-726, doi:10.1016/j.ygyno.2021.04.016.

13. Roque, D.M.; Ratner, E.S.; Silasi, D.A.; Azodi, M.; Rutherford, T.J.; Schwartz, P.E.; Nelson, W.K.; Santin, A.D. Weekly ixabepilone with or without biweekly bevacizumab in the treatment of recurrent or persistent uterine and ovarian/primary peritoneal/fallopian tube cancers: A retrospective review. *Gynecol Oncol* **2015**, *137*, 392-400, doi:10.1016/j.ygyno.2015.03.008.
14. O'Malley, D.M.; Bariani, G.M.; Cassier, P.A.; Marabelle, A.; Hansen, A.R.; De Jesus Acosta, A.; Miller, W.H., Jr.; Safra, T.; Italiano, A.; Mileshkin, L.; et al. Pembrolizumab in Patients With Microsatellite Instability-High Advanced Endometrial Cancer: Results From the KEYNOTE-158 Study. *J Clin Oncol* **2022**, *40*, 752-761, doi:10.1200/jco.21.01874.
15. Konstantinopoulos, P.A.; Luo, W.; Liu, J.F.; Gulhan, D.C.; Krasner, C.; Ishizuka, J.J.; Gockley, A.A.; Buss, M.; Growdon, W.B.; Crowe, H.; et al. Phase II Study of Avelumab in Patients With Mismatch Repair Deficient and Mismatch Repair Proficient Recurrent/Persistent Endometrial Cancer. *J Clin Oncol* **2019**, *37*, 2786-2794, doi:10.1200/jco.19.01021.
16. Colomba, E.; Alexandre, J.; Le Teuff, G.; Genestie, C.; Coupez, D.; Coquard, I.R.; Brachet, P.E.; de Percin, S.; Sajous, C.; Fabbro, M.; et al. Response to first line platinum-based chemotherapy in mismatch repair deficient (MMRd)/ microsatellite instability high (MSI-high) endometrial carcinoma. *Gynecol Oncol* **2023**, *169*, 78-84, doi:10.1016/j.ygyno.2022.11.029.
17. Bellone, S.; Roque, D.M.; Siegel, E.R.; Buza, N.; Hui, P.; Bonazzoli, E.; Guglielmi, A.; Zammataro, L.; Nagarkatti, N.; Zaidi, S.; et al. A phase 2 evaluation of pembrolizumab for recurrent Lynch-like versus sporadic endometrial cancers with microsatellite instability. *Cancer* **2022**, *128*, 1206-1218, doi:10.1002/cncr.34025.
18. Antill, Y.; Kok, P.S.; Robledo, K.; Yip, S.; Cummins, M.; Smith, D.; Spurdle, A.; Barnes, E.; Lee, Y.C.; Friedlander, M.; et al. Clinical activity of durvalumab for patients with advanced mismatch repair-deficient and repair-proficient endometrial cancer. A nonrandomized phase 2 clinical trial. *J Immunother Cancer* **2021**, *9*, doi:10.1136/jitc-2020-002255.
19. Berton, D.; Pautier, P.; Lorusso, D.; Gennigens, C.; Gladieff, L.; Kryzhanivska, A.; Bowman, J.; Tian, C.; Cornfeld, M.; Van Gorp, T. Antitumor activity and safety of the PD-1 inhibitor retifanlimab in patients with recurrent microsatellite instability-high or deficient mismatch repair endometrial cancer: Final safety and efficacy results from cohort H of the POD1UM-101 phase I study. *Gynecol Oncol* **2024**, *186*, 191-198, doi:10.1016/j.ygyno.2024.05.025.
20. André, T.; Berton, D.; Curigliano, G.; Sabatier, R.; Tinker, A.V.; Oaknin, A.; Ellard, S.; de Braud, F.; Arkenau, H.T.; Trigo, J.; et al. Antitumor Activity and Safety of Dostarlimab Monotherapy in Patients With Mismatch Repair Deficient Solid Tumors: A Nonrandomized Controlled Trial. *JAMA Netw Open* **2023**, *6*, e2341165, doi:10.1001/jamanetworkopen.2023.41165.
21. Rubinstein, M.M.; Doria, E.R.; Konner, J.; Lichtman, S.; Zhou, Q.; Iasonos, A.; Sarasohn, D.; Troso-Sandoval, T.; Friedman, C.; O'Cearbhaill, R.; et al. Durvalumab with or without tremelimumab in patients with persistent or recurrent endometrial cancer or endometrial carcinosarcoma: A randomized open-label phase 2 study. *Gynecol Oncol* **2023**, *169*, 64-69, doi:10.1016/j.ygyno.2022.11.028.
22. Post, C.C.B.; Westermann, A.M.; Boere, I.A.; Witteveen, P.O.; Ottevanger, P.B.; Sonke, G.S.; Lalisang, R.I.; Putter, H.; Meershoek-Klein Kranenbarg, E.; Braak, J.; et al. Efficacy and safety of durvalumab with olaparib in metastatic or recurrent endometrial cancer (phase II DOMEC trial). *Gynecol Oncol* **2022**, *165*, 223-229, doi:10.1016/j.ygyno.2022.02.025.
23. Konstantinopoulos, P.A.; Gockley, A.A.; Xiong, N.; Krasner, C.; Horowitz, N.; Campos, S.; Wright, A.A.; Liu, J.F.; Shea, M.; Yeku, O.; et al. Evaluation of Treatment With Talazoparib and Avelumab in Patients With Recurrent Mismatch Repair Proficient Endometrial Cancer. *JAMA Oncol* **2022**, *8*, 1317-1322, doi:10.1001/jamaoncol.2022.2181.
24. Wei, W.; Ban, X.; Yang, F.; Li, J.; Cheng, X.; Zhang, R.; Huang, X.; Huang, Y.; Li, Q.; Qiu, Y.; et al. Phase II trial of efficacy, safety and biomarker analysis of sintilimab plus anlotinib for patients with recurrent or advanced endometrial cancer. *J Immunother Cancer* **2022**, *10*, doi:10.1136/jitc-2021-004338.

25. Lheureux, S.; Matei, D.E.; Konstantinopoulos, P.A.; Wang, B.X.; Gadalla, R.; Block, M.S.; Jewell, A.; Gaillard, S.L.; McHale, M.; McCourt, C.; et al. Translational randomized phase II trial of cabozantinib in combination with nivolumab in advanced, recurrent, or metastatic endometrial cancer. *J Immunother Cancer* **2022**, *10*, doi:10.1136/jitc-2021-004233.
26. Cui, Q.; Mao, Y.; Hu, Y.; Ma, D.; Liu, H. Anlotinib in recurrent or metastatic endometrial cancer. *Int J Gynecol Cancer* **2022**, *32*, 1147-1152, doi:10.1136/ijgc-2022-003345.
27. How, J.A.; Jazaeri, A.A.; Fu, S.; Rodon Ahnert, J.; Gong, J.; Stephen, B.; Ferreira Dalla Pria, H.; Bhosale, P.; Johnson, A.; Yuan, Y.; et al. Clinical Outcomes of Patients with Recurrent Microsatellite-Stable Endometrial Cancer in Early-Phase Immunotherapy Clinical Trials. *Cancers (Basel)* **2022**, *14*, doi:10.3390/cancers14153695.
28. De Jaeghere, E.A.; Tuyaerts, S.; Van Nuffel, A.M.T.; Belmans, A.; Bogaerts, K.; Baiden-Amissah, R.; Lippens, L.; Vuylsteke, P.; Henry, S.; Trinh, X.B.; et al. Pembrolizumab, radiotherapy, and an immunomodulatory five-drug cocktail in pretreated patients with persistent, recurrent, or metastatic cervical or endometrial carcinoma: Results of the phase II PRIMMO study. *Cancer Immunol Immunother* **2023**, *72*, 475-491, doi:10.1007/s00262-022-03253-x.
29. Madariaga, A.; Garg, S.; Tchrakian, N.; Dhani, N.C.; Jimenez, W.; Welch, S.; MacKay, H.; Ethier, J.L.; Gilbert, L.; Li, X.; et al. Clinical outcome and biomarker assessments of a multi-centre phase II trial assessing niraparib with or without dostarlimab in recurrent endometrial carcinoma. *Nat Commun* **2023**, *14*, 1452, doi:10.1038/s41467-023-37084-w.
30. Taylor, M.H.; Lee, C.H.; Makker, V.; Rasco, D.; Dutcus, C.E.; Wu, J.; Stepan, D.E.; Shumaker, R.C.; Motzer, R.J. Phase IB/II Trial of Lenvatinib Plus Pembrolizumab in Patients With Advanced Renal Cell Carcinoma, Endometrial Cancer, and Other Selected Advanced Solid Tumors. *J Clin Oncol* **2020**, *38*, 1154-1163, doi:10.1200/jco.19.01598.
31. Kim, J.; Noh, J.J.; Lee, T.K.; Kim, S.I.; Lee, J.Y.; Lee, J.W.; Kim, J.W. Real-world experience of pembrolizumab and lenvatinib in recurrent endometrial cancer: A multicenter study in Korea. *Gynecol Oncol* **2022**, *165*, 369-375, doi:10.1016/j.ygyno.2022.02.020.
32. How, J.A.; Patel, S.; Fellman, B.; Lu, K.H.; Hwu, P.; Ramondetta, L.M.; Westin, S.N.; Fleming, N.D.; Soliman, P.T.; Jazaeri, A.A. Toxicity and efficacy of the combination of pembrolizumab with recommended or reduced starting doses of lenvatinib for treatment of recurrent endometrial cancer. *Gynecol Oncol* **2021**, *162*, 24-31, doi:10.1016/j.ygyno.2021.04.034.
33. Chiba, Y.; Kagabu, M.; Osakabe, M.; Ito, R.; Sato, S.; Takatori, E.; Kaido, Y.; Nagasawa, T.; Shoji, T.; Yanagawa, N.; et al. A single-institution retrospective exploratory analysis on the effectiveness and safety of lenvatinib plus pembrolizumab for advanced endometrial cancer: insights from ProMisE molecular classification system. *Jpn J Clin Oncol* **2024**, *54*, 424-433, doi:10.1093/jjco/hyad192.
34. Makker, V.; Aghajanian, C.; Cohn, A.L.; Romeo, M.; Bratos, R.; Brose, M.S.; Messing, M.; Dutta, L.; Dutcus, C.E.; Huang, J.; et al. A Phase Ib/II Study of Lenvatinib and Pembrolizumab in Advanced Endometrial Carcinoma (Study 111/KEYNOTE-146): Long-Term Efficacy and Safety Update. *J Clin Oncol* **2023**, *41*, 974-979, doi:10.1200/jco.22.01021.
35. Shang, X.; Su, H.; Chen, X.; Wang, Y.; Wan, X.; Zhang, Y.; Jin, Y.; Feng, F. Low-dose lenvatinib and anti-programmed cell death protein-1 combination therapy in patients with heavily pre-treated recurrent ovarian and endometrial cancer: a pilot study. *Int J Gynecol Cancer* **2024**, *34*, 1203-1210, doi:10.1136/ijgc-2024-005331.
36. Tochigi, M.; Shigeta, S.; Shimada, M.; Miyahara, S.; Hasegawa-Minato, J.; Shibuya, Y.; Ishibashi, M.; Hashimoto, C.; Tokunaga, H.; Yaegashi, N. Lenvatinib plus Pembrolizumab Combination Therapy for Advanced or Recurrent Endometrial Cancer: A Single-Center, Retrospective Analysis. *Tohoku J Exp Med* **2024**, *262*, 85-95, doi:10.1620/tjem.2023.J095.
37. Zammarrelli, W.A., 3rd; Ma, W.; Espino, K.; Gordhandas, S.; Yeoshoua, E.; Ehmann, S.; Zhou, Q.; Iasonos, A.; Abu-Rustum, N.R.; Aghajanian, C.; et al. Adverse events and oncologic outcomes with combination lenvatinib and pembrolizumab for the treatment of recurrent endometrial cancer. *Gynecol Oncol* **2023**, *178*, 27-35, doi:10.1016/j.ygyno.2023.09.010.

38. Makker, V.; Colombo, N.; Casado Herráez, A.; Monk, B.J.; Mackay, H.; Santin, A.D.; Miller, D.S.; Moore, R.G.; Baron-Hay, S.; Ray-Coquard, I.; et al. Lenvatinib Plus Pembrolizumab in Previously Treated Advanced Endometrial Cancer: Updated Efficacy and Safety From the Randomized Phase III Study 309/KEYNOTE-775. *J Clin Oncol* **2023**, *41*, 2904-2910, doi:10.1200/jco.22.02152.
39. Bafaloukos, D.; Aravantinos, G.; Samonis, G.; Katsifis, G.; Bakoyiannis, C.; Skarlos, D.; Kosmidis, P. Carboplatin, methotrexate and 5-fluorouracil in combination with medroxyprogesterone acetate (JMF-M) in the treatment of advanced or recurrent endometrial carcinoma: A Hellenic cooperative oncology group study. *Oncology* **1999**, *56*, 198-201, doi:10.1159/000011965.
40. Colon-Otero, G.; Zanfagnin, V.; Hou, X.; Foster, N.R.; Asmus, E.J.; Wahner Hendrickson, A.; Jatoi, A.; Block, M.S.; Langstraat, C.L.; Glaser, G.E.; et al. Phase II trial of ribociclib and letrozole in patients with relapsed oestrogen receptor-positive ovarian or endometrial cancers. *ESMO Open* **2020**, *5*, e000926, doi:10.1136/esmoopen-2020-000926.
41. Covens, A.L.; Filiaci, V.; Gersell, D.; Lutman, C.V.; Bonebrake, A.; Lee, Y.C. Phase II study of fulvestrant in recurrent/metastatic endometrial carcinoma: a Gynecologic Oncology Group study. *Gynecol Oncol* **2011**, *120*, 185-188, doi:10.1016/j.ygyno.2010.10.015.
42. Karagol, H.; Saip, P.; Uygun, K.; Kucucuk, S.; Aydiner, A.; Topuz, E. Evaluation of prognostic factors and comparison of systemic treatment modalities in patients with recurrent or metastatic endometrial carcinoma. *Med Oncol* **2006**, *23*, 543-548, doi:10.1385/mo:23:4:543.
43. Pautier, P.; Vergote, I.; Joly, F.; Melichar, B.; Kutarska, E.; Hall, G.; Lisyanskaya, A.; Reed, N.; Oaknin, A.; Ostapenko, V.; et al. A Phase 2, Randomized, Open-Label Study of Irosustat Versus Megestrol Acetate in Advanced Endometrial Cancer. *Int J Gynecol Cancer* **2017**, *27*, 258-266, doi:10.1097/igc.0000000000000862.
44. Rose, P.G.; Brunetto, V.L.; VanLe, L.; Bell, J.; Walker, J.L.; Lee, R.B. A phase II trial of anastrozole in advanced recurrent or persistent endometrial carcinoma: a Gynecologic Oncology Group study. *Gynecol Oncol* **2000**, *78*, 212-216, doi:10.1006/gyno.2000.5865.
45. Emons, G.; Günthert, A.; Thiel, F.C.; Camara, O.; Strauss, H.G.; Breitbach, G.P.; Kölbl, H.; Reimer, T.; Finas, D.; Rensing, K. Phase II study of fulvestrant 250 mg/month in patients with recurrent or metastatic endometrial cancer: a study of the Arbeitsgemeinschaft Gynäkologische Onkologie. *Gynecol Oncol* **2013**, *129*, 495-499, doi:10.1016/j.ygyno.2013.02.039.
46. Emons, G.; Gorchev, G.; Harter, P.; Wimberger, P.; Stähle, A.; Hanker, L.; Hilpert, F.; Beckmann, M.W.; Dall, P.; Gründker, C.; et al. Efficacy and safety of AEZS-108 (LHRH agonist linked to doxorubicin) in women with advanced or recurrent endometrial cancer expressing LHRH receptors: a multicenter phase 2 trial (AGO-GYN5). *Int J Gynecol Cancer* **2014**, *24*, 260-265, doi:10.1097/igc.0000000000000044.
47. Lindemann, K.; Malander, S.; Christensen, R.D.; Mirza, M.R.; Kristensen, G.B.; Aavall-Lundqvist, E.; Vergote, I.; Rosenberg, P.; Boman, K.; Nordstrøm, B. Examestane in advanced or recurrent endometrial carcinoma: a prospective phase II study by the Nordic Society of Gynecologic Oncology (NSGO). *BMC Cancer* **2014**, *14*, 68, doi:10.1186/1471-2407-14-68.
48. Whitney, C.W.; Brunetto, V.L.; Zaino, R.J.; Lentz, S.S.; Sorosky, J.; Armstrong, D.K.; Lee, R.B. Phase II study of medroxyprogesterone acetate plus tamoxifen in advanced endometrial carcinoma: a Gynecologic Oncology Group study. *Gynecol Oncol* **2004**, *92*, 4-9, doi:10.1016/j.ygyno.2003.09.018.
49. Thigpen, T.; Brady, M.F.; Homesley, H.D.; Soper, J.T.; Bell, J. Tamoxifen in the treatment of advanced or recurrent endometrial carcinoma: a Gynecologic Oncology Group study. *J Clin Oncol* **2001**, *19*, 364-367, doi:10.1200/jco.2001.19.2.364.
50. Thigpen, J.T.; Brady, M.F.; Alvarez, R.D.; Adelson, M.D.; Homesley, H.D.; Manetta, A.; Soper, J.T.; Given, F.T. Oral medroxyprogesterone acetate in the treatment of advanced or recurrent endometrial carcinoma: a dose-response study by the Gynecologic Oncology Group. *J Clin Oncol* **1999**, *17*, 1736-1744, doi:10.1200/jco.1999.17.6.1736.

51. Piver, M.S.; Lele, S.B.; Patsner, B.; Emrich, L.J. Melphalan, 5-fluorouracil, and medroxyprogesterone acetate in metastatic endometrial carcinoma. *Obstet Gynecol* **1986**, *67*, 261-264, doi:10.1097/00006250-198602000-00019.
52. Covens, A.; Thomas, G.; Shaw, P.; Ackerman, I.; Osborne, R.; Lukka, H.; Carey, M.; Franssen, E.; Roche, K. A phase II study of leuprolide in advanced/recurrent endometrial cancer. *Gynecol Oncol* **1997**, *64*, 126-129, doi:10.1006/gyno.1996.4544.
53. Ayoub, J.; Audet-Lapointe, P.; Méthot, Y.; Hanley, J.; Beaulieu, R.; Chemaly, R.; Cormier, A.; Déry, J.P.; Drouin, P.; Gauthier, P.; et al. Efficacy of sequential cyclical hormonal therapy in endometrial cancer and its correlation with steroid hormone receptor status. *Gynecol Oncol* **1988**, *31*, 327-337, doi:10.1016/s0090-8258(88)80012-x.
54. Pandya, K.J.; Yeap, B.Y.; Weiner, L.M.; Krook, J.E.; Erban, J.K.; Schinella, R.A.; Davis, T.E. Megestrol and tamoxifen in patients with advanced endometrial cancer: an Eastern Cooperative Oncology Group Study (E4882). *Am J Clin Oncol* **2001**, *24*, 43-46, doi:10.1097/00000421-200102000-00007.
55. Lentz, S.S.; Brady, M.F.; Major, F.J.; Reid, G.C.; Soper, J.T. High-dose megestrol acetate in advanced or recurrent endometrial carcinoma: a Gynecologic Oncology Group Study. *J Clin Oncol* **1996**, *14*, 357-361, doi:10.1200/jco.1996.14.2.357.
56. Lhommé, C.; Vennin, P.; Callet, N.; Lesimple, T.; Achard, J.L.; Chauvergne, J.; Luporsi, E.; Chinet-Charrot, P.; Coudert, B.; Couette, J.E.; et al. A multicenter phase II study with triptorelin (sustained-release LHRH agonist) in advanced or recurrent endometrial carcinoma: a French anticancer federation study. *Gynecol Oncol* **1999**, *75*, 187-193, doi:10.1006/gyno.1999.5538.
57. Rendina, G.M.; Donadio, C.; Fabri, M.; Mazzoni, P.; Nazzicone, P. Tamoxifen and medroxyprogesterone therapy for advanced endometrial carcinoma. *Eur J Obstet Gynecol Reprod Biol* **1984**, *17*, 285-291, doi:10.1016/0028-2243(84)90071-6.
58. Kulkarni, A.; Wright, N.M.A.; Forget, A.N.; Ramsay, T.; Mallick, R.; Weberpals, J.I. Should we abandon hormonal therapy in endometrial cancer? Outcomes of recurrent and metastatic endometrial cancer treated with systemic progestins. *Cancer Med* **2023**, *12*, 16173-16180, doi:10.1002/cam4.6276.
59. Fiorica, J.V.; Brunetto, V.L.; Hanjani, P.; Lentz, S.S.; Mannel, R.; Andersen, W. Phase II trial of alternating courses of megestrol acetate and tamoxifen in advanced endometrial carcinoma: a Gynecologic Oncology Group study. *Gynecol Oncol* **2004**, *92*, 10-14, doi:10.1016/j.ygyno.2003.11.008.
60. Hoffman, M.S.; Roberts, W.S.; Cavanagh, D.; Praphat, H.; Solomon, P.; Lyman, G.H. Treatment of recurrent and metastatic endometrial cancer with cisplatin, doxorubicin, cyclophosphamide, and megestrol acetate. *Gynecol Oncol* **1989**, *35*, 75-77, doi:10.1016/0090-8258(89)90016-4.
61. Mileshkin, L.; Edmondson, R.; O'Connell, R.L.; Sjoquist, K.M.; Andrews, J.; Jyothirmayi, R.; Beale, P.; Bonaventura, T.; Goh, J.; Hall, M.; et al. Phase 2 study of anastrozole in recurrent estrogen (ER)/progesterone (PR) positive endometrial cancer: The PARAGON trial - ANZGOG 0903. *Gynecol Oncol* **2019**, *154*, 29-37, doi:10.1016/j.ygyno.2019.05.007.
62. Covens, A.; Brunetto, V.L.; Markman, M.; Orr, J.W.; Lentz, S.S.; Benda, J. Phase II trial of danazol in advanced, recurrent, or persistent endometrial cancer: a Gynecologic Oncology Group study. *Gynecol Oncol* **2003**, *89*, 470-474, doi:10.1016/s0090-8258(03)00149-5.
63. Asbury, R.F.; Brunetto, V.L.; Lee, R.B.; Reid, G.; Rocereto, T.F. Goserelin acetate as treatment for recurrent endometrial carcinoma: a Gynecologic Oncology Group study. *Am J Clin Oncol* **2002**, *25*, 557-560, doi:10.1097/00000421-200212000-00004.
64. Ramondetta, L.M.; Johnson, A.J.; Sun, C.C.; Atkinson, N.; Smith, J.A.; Jung, M.S.; Broaddus, R.; Iyer, R.B.; Burke, T. Phase 2 trial of mifepristone (RU-486) in advanced or recurrent endometrioid adenocarcinoma or low-grade endometrial stromal sarcoma. *Cancer* **2009**, *115*, 1867-1874, doi:10.1002/cncr.24197.

65. Konstantinopoulos, P.A.; Lee, E.K.; Xiong, N.; Krasner, C.; Campos, S.; Kolin, D.L.; Liu, J.F.; Horowitz, N.; Wright, A.A.; Bouberhan, S.; et al. A Phase II, Two-Stage Study of Letrozole and Abemaciclib in Estrogen Receptor-Positive Recurrent Endometrial Cancer. *J Clin Oncol* **2023**, *41*, 599-608, doi:10.1200/jco.22.00628.
66. Emons, G.; Kurzeder, C.; Schmalfeldt, B.; Neuser, P.; de Gregorio, N.; Pfisterer, J.; Park-Simon, T.W.; Mahner, S.; Schröder, W.; Lück, H.J.; et al. Temsirolimus in women with platinum-refractory/resistant ovarian cancer or advanced/recurrent endometrial carcinoma. A phase II study of the AGO-study group (AGO-GYN8). *Gynecol Oncol* **2016**, *140*, 450-456, doi:10.1016/j.ygyno.2015.12.025.
67. Ray-Coquard, I.; Favier, L.; Weber, B.; Roemer-Becuwe, C.; Bougnoux, P.; Fabbro, M.; Floquet, A.; Joly, F.; Plantade, A.; Paraiso, D.; et al. Everolimus as second- or third-line treatment of advanced endometrial cancer: ENDORAD, a phase II trial of GINECO. *Br J Cancer* **2013**, *108*, 1771-1777, doi:10.1038/bjc.2013.183.
68. Makker, V.; Recio, F.O.; Ma, L.; Matulonis, U.A.; Lauchle, J.O.; Parmar, H.; Gilbert, H.N.; Ware, J.A.; Zhu, R.; Lu, S.; et al. A multicenter, single-arm, open-label, phase 2 study of apitolisib (GDC-0980) for the treatment of recurrent or persistent endometrial carcinoma (MAGGIE study). *Cancer* **2016**, *122*, 3519-3528, doi:10.1002/cncr.30286.
69. Matulonis, U.; Vergote, I.; Backes, F.; Martin, L.P.; McMeekin, S.; Birrer, M.; Campana, F.; Xu, Y.; Egile, C.; Ghamande, S. Phase II study of the PI3K inhibitor pilaralisib (SAR245408; XL147) in patients with advanced or recurrent endometrial carcinoma. *Gynecol Oncol* **2015**, *136*, 246-253, doi:10.1016/j.ygyno.2014.12.019.
70. Fleming, G.F.; Filiaci, V.L.; Marzullo, B.; Zaino, R.J.; Davidson, S.A.; Pearl, M.; Makker, V.; Burke, J.J., 2nd; Zweizig, S.L.; Van Le, L.; et al. Temsirolimus with or without megestrol acetate and tamoxifen for endometrial cancer: a gynecologic oncology group study. *Gynecol Oncol* **2014**, *132*, 585-592, doi:10.1016/j.ygyno.2014.01.015.
71. Heudel, P.E.; Fabbro, M.; Roemer-Becuwe, C.; Kaminsky, M.C.; Arnaud, A.; Joly, F.; Roche-Forestier, S.; Meunier, J.; Foa, C.; You, B.; et al. Phase II study of the PI3K inhibitor BKM120 in patients with advanced or recurrent endometrial carcinoma: a stratified type I-type II study from the GINECO group. *Br J Cancer* **2017**, *116*, 303-309, doi:10.1038/bjc.2016.430.
72. Soliman, P.T.; Westin, S.N.; Iglesias, D.A.; Fellman, B.M.; Yuan, Y.; Zhang, Q.; Yates, M.S.; Broaddus, R.R.; Slomovitz, B.M.; Lu, K.H.; et al. Everolimus, Letrozole, and Metformin in Women with Advanced or Recurrent Endometrioid Endometrial Cancer: A Multi-Center, Single Arm, Phase II Study. *Clin Cancer Res* **2020**, *26*, 581-587, doi:10.1158/1078-0432.Ccr-19-0471.
73. Heudel, P.; Frenel, J.S.; Dalban, C.; Bazan, F.; Joly, F.; Arnaud, A.; Abdeddaim, C.; Chevalier-Place, A.; Augereau, P.; Pautier, P.; et al. Safety and Efficacy of the mTOR Inhibitor, Vistusertib, Combined With Anastrozole in Patients With Hormone Receptor-Positive Recurrent or Metastatic Endometrial Cancer: The VICTORIA Multicenter, Open-label, Phase 1/2 Randomized Clinical Trial. *JAMA Oncol* **2022**, *8*, 1001-1009, doi:10.1001/jamaoncol.2022.1047.
74. Oza, A.M.; Elit, L.; Tsao, M.S.; Kamel-Reid, S.; Biagi, J.; Provencher, D.M.; Gotlieb, W.H.; Hoskins, P.J.; Ghatage, P.; Tonkin, K.S.; et al. Phase II study of temsirolimus in women with recurrent or metastatic endometrial cancer: a trial of the NCIC Clinical Trials Group. *J Clin Oncol* **2011**, *29*, 3278-3285, doi:10.1200/jco.2010.34.1578.
75. Oza, A.M.; Pignata, S.; Poveda, A.; McCormack, M.; Clamp, A.; Schwartz, B.; Cheng, J.; Li, X.; Campbell, K.; Dodion, P.; et al. Randomized Phase II Trial of Ridaforolimus in Advanced Endometrial Carcinoma. *J Clin Oncol* **2015**, *33*, 3576-3582, doi:10.1200/jco.2014.58.8871.
76. Slomovitz, B.M.; Jiang, Y.; Yates, M.S.; Soliman, P.T.; Johnston, T.; Nowakowski, M.; Levenback, C.; Zhang, Q.; Ring, K.; Munsell, M.F.; et al. Phase II study of everolimus and letrozole in patients with recurrent endometrial carcinoma. *J Clin Oncol* **2015**, *33*, 930-936, doi:10.1200/jco.2014.58.3401.

77. Del Campo, J.M.; Birrer, M.; Davis, C.; Fujiwara, K.; Gollerkeri, A.; Gore, M.; Houk, B.; Lau, S.; Poveda, A.; González-Martín, A.; et al. A randomized phase II non-comparative study of PF-04691502 and gedatolisib (PF-05212384) in patients with recurrent endometrial cancer. *Gynecol Oncol* **2016**, *142*, 62-69, doi:10.1016/j.ygyno.2016.04.019.
78. Slomovitz, B.M.; Filiaci, V.L.; Walker, J.L.; Taub, M.C.; Finkelstein, K.A.; Moroney, J.W.; Fleury, A.C.; Muller, C.Y.; Holman, L.L.; Copeland, L.J.; et al. A randomized phase II trial of everolimus and letrozole or hormonal therapy in women with advanced, persistent or recurrent endometrial carcinoma: A GOG Foundation study. *Gynecol Oncol* **2022**, *164*, 481-491, doi:10.1016/j.ygyno.2021.12.031.
79. Myers, A.P.; Konstantinopoulos, P.A.; Barry, W.T.; Luo, W.; Broaddus, R.R.; Makker, V.; Drapkin, R.; Liu, J.; Doyle, A.; Horowitz, N.S.; et al. Phase II, 2-stage, 2-arm, PIK3CA mutation stratified trial of MK-2206 in recurrent endometrial cancer. *Int J Cancer* **2020**, *147*, 413-422, doi:10.1002/ijc.32783.
80. Westin, S.N.; Sill, M.W.; Coleman, R.L.; Waggoner, S.; Moore, K.N.; Mathews, C.A.; Martin, L.P.; Modesitt, S.C.; Lee, S.; Ju, Z.; et al. Safety lead-in of the MEK inhibitor trametinib in combination with GSK2141795, an AKT inhibitor, in patients with recurrent endometrial cancer: An NRG Oncology/GOG study. *Gynecol Oncol* **2019**, *155*, 420-428, doi:10.1016/j.ygyno.2019.09.024.
81. Rubinstein, M.M.; Hyman, D.M.; Caird, I.; Won, H.; Soldan, K.; Seier, K.; Iasonos, A.; Tew, W.P.; O'Cearbhaill, R.E.; Grisham, R.N.; et al. Phase 2 study of LY3023414 in patients with advanced endometrial cancer harboring activating mutations in the PI3K pathway. *Cancer* **2020**, *126*, 1274-1282, doi:10.1002/cncr.32677.
82. Han, S.N.; Oza, A.; Colombo, N.; Oaknin, A.; Raspagliesi, F.; Wenham, R.M.; Braicu, E.I.; Jewell, A.; Makker, V.; Krell, J.; et al. A randomized phase 2 study of sapanisertib in combination with paclitaxel versus paclitaxel alone in women with advanced, recurrent, or persistent endometrial cancer. *Gynecol Oncol* **2023**, *178*, 110-118, doi:10.1016/j.ygyno.2023.09.013.
83. Santin, A.D.; Filiaci, V.; Bellone, S.; Ratner, E.S.; Mathews, C.A.; Cantuaria, G.; Gunderson, C.C.; Rutledge, T.; Buttin, B.M.; Lankes, H.A.; et al. Phase II evaluation of copanlisib, a selective inhibitor of Pi3kca, in patients with persistent or recurrent endometrial carcinoma harboring PIK3CA hotspot mutations: An NRG Oncology study (NRG-GY008). *Gynecol Oncol Rep* **2020**, *31*, 100532, doi:10.1016/j.gore.2019.100532.
84. Eskander, R.N.; Sill, M.W.; Beffa, L.; Moore, R.G.; Hope, J.M.; Musa, F.B.; Mannel, R.; Shahin, M.S.; Cantuaria, G.H.; Girda, E.; et al. Pembrolizumab plus Chemotherapy in Advanced Endometrial Cancer. *N Engl J Med* **2023**, *388*, 2159-2170, doi:10.1056/NEJMoa2302312.
85. Mirza, M.R.; Chase, D.M.; Slomovitz, B.M.; dePont Christensen, R.; Novák, Z.; Black, D.; Gilbert, L.; Sharma, S.; Valabrega, G.; Landrum, L.M.; et al. Dostarlimab for Primary Advanced or Recurrent Endometrial Cancer. *N Engl J Med* **2023**, *388*, 2145-2158, doi:10.1056/NEJMoa2216334.
86. Pignata, S.; Scambia, G.; Schettino, C.; Arenare, L.; Pisano, C.; Lombardi, D.; De Giorgi, U.; Andreetta, C.; Cinieri, S.; De Angelis, C.; et al. Carboplatin and paclitaxel plus avelumab compared with carboplatin and paclitaxel in advanced or recurrent endometrial cancer (MITO END-3): a multicentre, open-label, randomised, controlled, phase 2 trial. *Lancet Oncol* **2023**, *24*, 286-296, doi:10.1016/s1470-2045(23)00016-5.
87. Westin, S.N.; Moore, K.; Chon, H.S.; Lee, J.Y.; Thomes Pepin, J.; Sundborg, M.; Shai, A.; de la Garza, J.; Nishio, S.; Gold, M.A.; et al. Durvalumab Plus Carboplatin/Paclitaxel Followed by Maintenance Durvalumab With or Without Olaparib as First-Line Treatment for Advanced Endometrial Cancer: The Phase III DUO-E Trial. *J Clin Oncol* **2024**, *42*, 283-299, doi:10.1200/jco.23.02132.
88. Colombo, N.; Biagioli, E.; Harano, K.; Galli, F.; Hudson, E.; Antill, Y.; Choi, C.H.; Rabaglio, M.; Marmé, F.; Marth, C.; et al. Atezolizumab and chemotherapy for advanced or recurrent

endometrial cancer (AtTEnd): a randomised, double-blind, placebo-controlled, phase 3 trial. *Lancet Oncol* **2024**, *25*, 1135-1146, doi:10.1016/s1470-2045(24)00334-6.
89. Fleming, G.F.; Sill, M.W.; Darcy, K.M.; McMeekin, D.S.; Thigpen, J.T.; Adler, L.M.; Berek, J.S.; Chapman, J.A.; DiSilvestro, P.A.; Horowitz, I.R.; et al. Phase II trial of trastuzumab in women with advanced or recurrent, HER2-positive endometrial carcinoma: a Gynecologic Oncology Group study. *Gynecol Oncol* **2010**, *116*, 15-20, doi:10.1016/j.ygyno.2009.09.025.
90. Ahn, E.R.; Rothe, M.; Mangat, P.K.; Garrett-Mayer, E.; Ali-Ahmad, H.M.; Chan, J.; Maitland, M.L.; Patel, S.R.; Reese, Z.; Balmanoukian, A.S.; et al. Pertuzumab Plus Trastuzumab in Patients With Endometrial Cancer With ERBB2/3 Amplification, Overexpression, or Mutation: Results From the TAPUR Study. *JCO Precis Oncol* **2023**, *7*, e2200609, doi:10.1200/po.22.00609.
91. Arend, R.; Dholakia, J.; Castro, C.; Matulonis, U.; Hamilton, E.; Jackson, C.G.; LyBarger, K.; Goodman, H.M.; Duska, L.R.; Mahdi, H.; et al. DKK1 is a predictive biomarker for response to DKN-01: Results of a phase 2 basket study in women with recurrent endometrial carcinoma. *Gynecol Oncol* **2023**, *172*, 82-91, doi:10.1016/j.ygyno.2023.03.013.
92. Bender, D.; Sill, M.W.; Lankes, H.A.; Reyes, H.D.; Darus, C.J.; Delmore, J.E.; Rotmensch, J.; Gray, H.J.; Mannel, R.S.; Schilder, J.M.; et al. A phase II evaluation of cediranib in the treatment of recurrent or persistent endometrial cancer: An NRG Oncology/Gynecologic Oncology Group study. *Gynecol Oncol* **2015**, *138*, 507-512, doi:10.1016/j.ygyno.2015.07.018.
93. Coleman, R.L.; Sill, M.W.; Lankes, H.A.; Fader, A.N.; Finkler, N.J.; Hoffman, J.S.; Rose, P.G.; Sutton, G.P.; Drescher, C.W.; McMeekin, D.S.; et al. A phase II evaluation of aflibercept in the treatment of recurrent or persistent endometrial cancer: a Gynecologic Oncology Group study. *Gynecol Oncol* **2012**, *127*, 538-543, doi:10.1016/j.ygyno.2012.08.020.
94. Dizon, D.S.; Sill, M.W.; Schilder, J.M.; McGonigle, K.F.; Rahman, Z.; Miller, D.S.; Mutch, D.G.; Leslie, K.K. A phase II evaluation of nintedanib (BIBF-1120) in the treatment of recurrent or persistent endometrial cancer: an NRG Oncology/Gynecologic Oncology Group Study. *Gynecol Oncol* **2014**, *135*, 441-445, doi:10.1016/j.ygyno.2014.10.001.
95. Castonguay, V.; Lheureux, S.; Welch, S.; Mackay, H.J.; Hirte, H.; Fleming, G.; Morgan, R.; Wang, L.; Blattler, C.; Ivy, P.S.; et al. A phase II trial of sunitinib in women with metastatic or recurrent endometrial carcinoma: a study of the Princess Margaret, Chicago and California Consortia. *Gynecol Oncol* **2014**, *134*, 274-280, doi:10.1016/j.ygyno.2014.05.016.
96. Dhani, N.C.; Hirte, H.W.; Wang, L.; Burnier, J.V.; Jain, A.; Butler, M.O.; Welch, S.; Fleming, G.F.; Hurteau, J.; Matsuo, K.; et al. Phase II Trial of Cabozantinib in Recurrent/Metastatic Endometrial Cancer: A Study of the Princess Margaret, Chicago, and California Consortia (NCI9322/PHL86). *Clin Cancer Res* **2020**, *26*, 2477-2486, doi:10.1158/1078-0432.Ccr-19-2576.
97. Powell, M.A.; Sill, M.W.; Goodfellow, P.J.; Benbrook, D.M.; Lankes, H.A.; Leslie, K.K.; Jeske, Y.; Mannel, R.S.; Spillman, M.A.; Lee, P.S.; et al. A phase II trial of brivanib in recurrent or persistent endometrial cancer: an NRG Oncology/Gynecologic Oncology Group Study. *Gynecol Oncol* **2014**, *135*, 38-43, doi:10.1016/j.ygyno.2014.07.083.
98. McMeekin, D.S.; Sill, M.W.; Benbrook, D.; Darcy, K.M.; Stearns-Kurosawa, D.J.; Eaton, L.; Yamada, S.D. A phase II trial of thalidomide in patients with refractory endometrial cancer and correlation with angiogenesis biomarkers: a Gynecologic Oncology Group study. *Gynecol Oncol* **2007**, *105*, 508-516, doi:10.1016/j.ygyno.2007.01.019.
99. Ren, Y.; Wang, T.; Cheng, X.; Ke, G.; Huang, Y.; Yang, H.; Huang, X.; Tian, W.; Wang, H. Efficacy and safety of apatinib in patients with recurrent uterine malignancy: a prospective, single-center, single-arm, phase 2 study. *Ann Transl Med* **2023**, *11*, 106, doi:10.21037/atm-22-6463.
100. Vergote, I.; Powell, M.A.; Teneriello, M.G.; Miller, D.S.; Garcia, A.A.; Mikheeva, O.N.; Bidzinski, M.; Cebotaru, C.L.; Dutcus, C.E.; Ren, M.; et al. Second-line lenvatinib in patients with recurrent endometrial cancer. *Gynecol Oncol* **2020**, *156*, 575-582, doi:10.1016/j.ygyno.2019.12.039.
101. Moore, K.N.; Sill, M.W.; Tenney, M.E.; Darus, C.J.; Griffin, D.; Werner, T.L.; Rose, P.G.; Behrens, R. A phase II trial of trebananib (AMG 386; IND#111071), a selective angiopoietin

1/2 neutralizing peptibody, in patients with persistent/recurrent carcinoma of the endometrium: An NRG/Gynecologic Oncology Group trial. *Gynecol Oncol* **2015**, *138*, 513-518, doi:10.1016/j.ygyno.2015.07.006.
102. Konecny, G.E.; Finkler, N.; Garcia, A.A.; Lorusso, D.; Lee, P.S.; Rocconi, R.P.; Fong, P.C.; Squires, M.; Mishra, K.; Upalawanna, A.; et al. Second-line dovitinib (TKI258) in patients with FGFR2-mutated or FGFR2-non-mutated advanced or metastatic endometrial cancer: a non-randomised, open-label, two-group, two-stage, phase 2 study. *Lancet Oncol* **2015**, *16*, 686-694, doi:10.1016/s1470-2045(15)70159-2.
103. Westermann, A.; Ottevanger, P.; Reyners, A.; Kroep, J.R.; Van Oijen, M.G.H.; Lalisang, R.; Witteveen, P.O. PAZEC: a Dutch Gynaecological Oncology Group open-label, multicenter, phase II study of pazopanib in metastatic and locally advanced hormone-resistant endometrial cancer. *Int J Gynecol Cancer* **2024**, *34*, 239-243, doi:10.1136/ijgc-2023-004781.
104. Backes, F.J.; Wei, L.; Chen, M.; Hill, K.; Dzwigalski, K.; Poi, M.; Phelps, M.; Salani, R.; Copeland, L.J.; Fowler, J.M.; et al. Phase I evaluation of lenvatinib and weekly paclitaxel in patients with recurrent endometrial, ovarian, fallopian tube, or primary peritoneal Cancer. *Gynecol Oncol* **2021**, *162*, 619-625, doi:10.1016/j.ygyno.2021.06.032.
105. Leslie, K.K.; Sill, M.W.; Lankes, H.A.; Fischer, E.G.; Godwin, A.K.; Gray, H.; Schilder, R.J.; Walker, J.L.; Tewari, K.; Hanjani, P.; et al. Lapatinib and potential prognostic value of EGFR mutations in a Gynecologic Oncology Group phase II trial of persistent or recurrent endometrial cancer. *Gynecol Oncol* **2012**, *127*, 345-350, doi:10.1016/j.ygyno.2012.07.127.
106. Leslie, K.K.; Sill, M.W.; Fischer, E.; Darcy, K.M.; Mannel, R.S.; Tewari, K.S.; Hanjani, P.; Wilken, J.A.; Baron, A.T.; Godwin, A.K.; et al. A phase II evaluation of gefitinib in the treatment of persistent or recurrent endometrial cancer: a Gynecologic Oncology Group study. *Gynecol Oncol* **2013**, *129*, 486-494, doi:10.1016/j.ygyno.2013.02.019.
107. Coleman, R.L.; Sill, M.W.; Thaker, P.H.; Bender, D.P.; Street, D.; McGuire, W.P.; Johnston, C.M.; Rotmensch, J. A phase II evaluation of selumetinib (AZD6244, ARRY-142886), a selective MEK-1/2 inhibitor in the treatment of recurrent or persistent endometrial cancer: an NRG Oncology/Gynecologic Oncology Group study. *Gynecol Oncol* **2015**, *138*, 30-35, doi:10.1016/j.ygyno.2015.04.005.
108. Dizon, D.S.; Blessing, J.A.; McMeekin, D.S.; Sharma, S.K.; Disilvestro, P.; Alvarez, R.D. Phase II trial of ixabepilone as second-line treatment in advanced endometrial cancer: gynecologic oncology group trial 129-P. *J Clin Oncol* **2009**, *27*, 3104-3108, doi:10.1200/jco.2008.20.6995.
109. McMeekin, S.; Dizon, D.; Barter, J.; Scambia, G.; Manzyuk, L.; Lisyanskaya, A.; Oaknin, A.; Ringuette, S.; Mukhopadhyay, P.; Rosenberg, J.; et al. Phase III randomized trial of second-line ixabepilone versus paclitaxel or doxorubicin in women with advanced endometrial cancer. *Gynecol Oncol* **2015**, *138*, 18-23, doi:10.1016/j.ygyno.2015.04.026.
110. Liu, J.F.; Xiong, N.; Campos, S.M.; Wright, A.A.; Krasner, C.; Schumer, S.; Horowitz, N.; Veneris, J.; Tayob, N.; Morrissey, S.; et al. Phase II Study of the WEE1 Inhibitor Adavosertib in Recurrent Uterine Serous Carcinoma. *J Clin Oncol* **2021**, *39*, 1531-1539, doi:10.1200/jco.20.03167.
111. Atkins, S.L.P.; Greer, Y.E.; Jenkins, S.; Gatti-Mays, M.E.; Houston, N.; Lee, S.; Lee, M.J.; Rastogi, S.; Sato, N.; Burks, C.; et al. A Single-Arm, Open-Label Phase II Study of ONC201 in Recurrent/Refractory Metastatic Breast Cancer and Advanced Endometrial Carcinoma. *Oncologist* **2023**, *28*, 919-e972, doi:10.1093/oncolo/oyad164.
112. Rimel, B.J.; Enserro, D.; Bender, D.P.; Jackson, C.G.; Tan, A.; Alluri, N.; Borowsky, M.; Moroney, J.; Hendrickson, A.W.; Backes, F.; et al. NRG-GY012: Randomized phase 2 study comparing olaparib, cediranib, and the combination of cediranib/olaparib in women with recurrent, persistent, or metastatic endometrial cancer. *Cancer* **2024**, *130*, 1234-1245, doi:10.1002/cncr.35151.
